# Supplementary material for: Social and structural barriers and facilitators to HIV healthcare and harm reduction services for people experiencing syndemics in Manitoba: study protocol
Source: BMJ Open. 2023 Aug 2;13(8):e067813. doi: 10.1136/bmjopen-2022-067813 (PMC10401247; doi:10.1136/bmjopen-2022-067813)
Supplement: Supplementary data [file bmjopen-2022-067813supp001.pdf]

# Terms of Reference for the Research Advisory Committee

## Background:

In Manitoba, injection drug use is the most common mode of HIV transmission since 2018. Those who inject drugs are more likely to test positive for HIV, syphilis and Hepatitis C, often at the same time. This is called a "syndemic" (many epidemics at the same time). For the last three years, syphilis infections have become rampant in Manitoba. If not found and treated, syphilis can cause devastating infection, including transmission to newborns. In addition to HIV and syphilis there are 11 other infections transmitted by sexual activity or blood products (called STBBI).
For some people, a convergence of socio-economic factors, mental health concerns, violence and exploitation, is driving a syndemic of new HIV and other STBBI cases, with disproportionate representation of women who inject drugs. Methamphetamine use is growing in Manitoba and is known to affect access to care and treatment. These determinants of health and disparities are exacerbated by COVID-19 related public health measures, as resources for testing and contact tracing for Covid-19 have resulted in decreased capacity for service providers to respond to STBBI and distribute harm reduction services. It is unknown how both biological sex differences and gender intersect with living conditions including experiences of violence and injection drug use.

This study seeks to know:

1. How many people in Manitoba (by sex, gender and other identifiers) living with HIV have other sexually transmitted and blood-borne infections (STBBI), before and during Covid-19?
2. Of those living with HIV, whom are most at risk of acquiring other STBBI, and why?
3. What are the barriers and gaps that put people at risk for infection and prevent them from getting appropriate care and treatment before and during Covid-19?
4. What are the resilience factors and promising practices that are associated with successful engagement of people living with HIV and who inject methamphetamine that endured during the Covid-19 pandemic?

Gaining an in-depth understanding of the experiences of women, men and non-binary persons living with HIV who use and don't use drugs can help us identify unique gaps as well as resilience factors that can inform and improve the Manitoba HIV Program, and result in tailored testing and appropriate clinical and program responses.

## Principal Investigators:

Dr. Zulma Rueda. University of Manitoba. Email: zulma.rueda@umanitoba.ca
Dr. Yoav Keynan University of Manitoba. Email: yoav.keynan@umanitoba.ca
Ms. Margaret Haworth-Brockman, University of Manitoba. Email: Margaret.Haworth-Brockman@umanitoba.ca

## Advisory Committee's Role and Purpose

*The Advisory Committee will advise the research team on the following aspects of the project:*

- ✓ Research approach and community-based methods
- ✓ Guidance the implementation of research.
- ✓ Promotion of the research study.

- ✓ Advice on community engagement plans.
- ✓ Interpretation of findings.
- ✓ Help to identify and strategize any potential solutions to community issues that may arise.
- ✓ At all times, strictly maintain confidentiality for all aspects of the research project as outlined in the University of Manitoba *Access and Privacy policy* and *Access and Privacy Procedure.*

- ✓ **Input and advice from the Advisory Committee will help to ensure that**:
  - ✓ The project/study is culturally appropriate for the diverse communities involved.
  - ✓ The research team conducts the project in the communities' best interests.
  - ✓ The research results and knowledge will be shared appropriately with communities and stakeholders.
  - ✓ Action plans associated with the research receive community input and are appropriate to community needs and concerns.

All Advisory Committee members will be eligible for compensation for their time involved. We expect there to be about 5-7 meetings over 12 months, with one or two meetings being in the early stages of the project development.

**Responsibilities from the research team:**

1. Ensure the ethical conduct of the research study.
2. Provide accurate information related to the project.
3. Investigate and answer the suggestions of the research advisory committee.

**Disclosures:**

- ✓ All member will declare any real or perceived conflict of interest at the outset of any meeting for management in accordance with the University of Manitoba *Conflicts of Interest Policy and Conflict of Interest Procedures.*
- ✓ All research project conducted within the University of Manitoba is governed by the *Responsible Conduct of Research Policy, Responsible Conduct of Research – Code of Research Ethics Policy, Responsible Conduct of Research – Investigation Procedure.*
- ✓ This research is funded by the Canadian Institute of Health Research; therefore, we follow and adhere to the *Tri-Agency Framework: Responsible Conduct of Research, including the framework and policies of Chapter 9: Research Involving the First Nations, Inuit and Métis Peoples of Canada.*

**Term**

This research is initially funded for one year.

**Memberships**

The Committee will be composed by the following members, in addition to the 3 principal investigators. Our meetings will be opened in a Good Way with the help of an Elder.

The number and composition of members comprises of:

1. Five or six community members – we are actively recruiting advisors now. We are inviting members who include:

a. Person who lives with HIV
b. Person who has current or former experience with injection drug use
c. Women or female who have experienced sexual assault, sexual violence, experiencing violence or sexual exploitation
d. Person who is experiencing homelessness or who are unsheltered
e. Person who identify themselves as 2SLGBTQQIA+
f. Person who belong to Indigenous population or racialized communities

2. Elder Margaret Lavallee, Elder in Residence, Ongomiizwin
3. Julianne Sanguins. Adjunct Scientist at the Manitoba Centre for Health Policy, and Assistant Professor at the Department of Community Health Sciences, University of Manitoba.
4. Tara Faye Myran, Metis Community member.
5. Ann Favel, Person with lived experience. Member of Sisters of Fire
6. Jaqueline Flett, Person with lived experience. Member of Sisters of Fire
7. Marj Schenkels, Person with lived experience. Member of Sisters of Fire
8. Nikki Daniels. Community member with lived experience.
9. Jody Jollimore. Executive Director at CATIE
10. Margaret Bryan. Manitoba Harm Reduction Network
11. Jacqueline Gahagan. Associate Vice-President, Research, Mount Saint Vincent University. Halifax, Nova Scotia.
12. Adrienne FA Meyers, Associate Director of the JC Wilt Infectious Diseases Research Centre
13. Neora Pick, Clinical Professor in the Infectious Diseases division of the Department of Medicine at the University of British Columbia, and Medical Director at the Oak Tree Clinic.
14. Veda Koncan, Projects Manager, Manitoba Harm Reduction Network
15. Chantal Daniels, Elder Support, Indigenous Institute of Health & Healing, Ongomiizwin

**Meeting frequency**
Meetings will be called by the Project Manager.
The committee will meet for five or seven 2-hour meetings either in person in Winnipeg or via teleconference/web-based technology for members residing outside Winnipeg.

**Sitting fees**
Research team will provide $20 CAD/per hour honorariums for people who are eligible for honorarium
Research team will cover costs for transportation for people residing in Winnipeg

**Contact:**
Dr. Zulma Rueda. University of Manitoba. Email: zulma.rueda@umanitoba.ca
Dr. Yoav Keynan University of Manitoba. Email: yoav.keynan@umanitoba.ca
Ms. Margaret Haworth-Brockman, University of Manitoba. Email: Margaret.Haworth-Brockman@umanitoba.ca

Cheryl Sobie. Research project coordinator. University of Manitoba. Email: Cheryl.Sobie@umanitoba.ca
