# Supplementary material for: Social and structural barriers and facilitators to HIV healthcare and harm reduction services for people experiencing syndemics in Manitoba: study protocol
Source: BMJ Open. 2023 Aug 2;13(8):e067813. doi: 10.1136/bmjopen-2022-067813 (PMC10401247; doi:10.1136/bmjopen-2022-067813)
Supplement: Supplementary data [file bmjopen-2022-067813supp002.pdf]

Peer Terms of Reference

Purpose:

We want to engage people with lived experience as peers in our project and work collaboratively to ensure all aspects of the research are shaped and guided by the perspectives of peers with lived experience. Peer engagement will inform the entire research process, from conducting research interviews, to skill sharing, workshops, and developing educational materials to share the results of our research. We recognize the unique knowledge and expertise of community members with lived experience, and we seek your guidance and input for our research study.

Background Information:

We are peers, clinicians and researchers who want to understand infections transmitted by sex or blood in people living with HIV, who may use drugs, as well as why people living with HIV are not getting the care they need. We are interested in knowing which factors, barriers and gaps prevent people from accessing care. We will also look for resilience factors that keep people linked to care.

We are interested in better understanding the impact of the COVID-19 pandemic and how it may have affected people newly diagnosed with HIV (between 2018-2021) in accessing health care and other resources. We want to know things such as:

- What was it like navigating the health care system during the pandemic?
- What are ways we can make things better for people to be linked to health care?
- What things are working well for people that keep them going to get health support and resources before and during the COVID-19 pandemic?

Project Time Period:

July 2022 – March 2023

Peer Roles available:

1. Peer Research Advisory Committee Member
2. Peer Support Worker
3. Peer Group Team Member

<u>Role (1) Peer Research Advisory Committee Member</u> (see Research Advisory Committee Terms of Reference Document). Key items to note:

How does this work?
You will join our committee which includes peers like you and some researchers. Together you will advise the research team about the best ways to:

- involve other people in the research study (using posters, or word of mouth, or in certain places);

- pay or compensate them for their time (for example, taxi coupons, bus passes, cash, or pre-paid VISAs);
- questions that are appropriate in interviews;
- how to understand what we find from the results;
- how to share the results of the study with the community;
- other education that the community or health care providers need to know. There may be other things you provide advice on.

Meeting frequency:

The Research Advisory Committee will meet for five or seven 2-hour meetings either in person in Winnipeg or via teleconference/web-based technology for members residing outside Winnipeg.

Compensation:

The research team will provide $20 CAD/per hour honorarium for peer research advisory committee members. Research team will cover costs for transportation (bus fare, taxi) for people residing in Winnipeg to attend the meeting.

Role (2) Peer Support Worker:

How does this work?

- Provide peer support for participants (people living with HIV) during research interview
- You will sit in the interview with participant and research team member to provide support as needed
- When we are scheduling interviews, we will ask participants if they would like a peer support worker at their interview
- Must sign confidentiality agreement and maintain confidentiality at all times
- We plan to interview 20-30 people; we are unsure at this point how many will require support
- The interview will take place in community settings such as Nine Circles, HSC HIV Clinic or another community location as determined by the participant and research team member

Time Required:

- 2-3 hours per interview session
- We anticipate interviews will take place between the end of August - October 2022

Role (3) Peer Research Team Member

How does this work?

- The purpose of the peer research team is to bring together community members with lived experience to work together to conduct research (interviews, data analysis, review findings), develop ideas for how we will share the results of our research, review comments from the advisory committee, and provide guidance and direction on the research for recruitment, community engagement, etc.
- The peer team is separate from the advisory board; however, a peer group member may also sit on the advisory board.
- The work of the peer team will include items such as:
    - Develop research skills and conduct research interviews, support data analysis and review findings of research
    - Discuss issues raised by the advisory board
    - Develop ideas and collaborate to prepare educational material for the community based on the results and findings of the research
    - To learn from each other either by skill sharing (i.e., doing computer courses provided by university, learning new skills related to grant writing/proposal development, learning technologies such as Zoom, etc.); we want to hear from YOU what you want to learn about
    - To advocate and share with the public what it is that is needed in Manitoba to break the stigma of HIV and drug use
- The peer team will consist of ten community members with lived experience
- Members will be selected by members that want to participate. If too many people apply, we will narrow out the group by selecting someone from each of the criteria in line with the research

Meetings:

Meeting Frequency & Location

The peer team will meet every two to three weeks for two hours. We will meet in person wherever possible at the University of Manitoba Bannatyne campus, or outside at a park. Should things change depending on the COVID-19 situation and safety guidelines we may have to meet online using Zoom or Teams.

Comfort & Safety

We will create a safe space for all group members to participate in, which includes psychological safety and cultural safety. Members can participate to the maximum comfortable amount.

We reject any form of discrimination or racism. We are committed for a safe and inclusive environment for everyone to participate. We do not judge each other and will respect each other at all times. We are committed to making your participation as accessible as possible. If there is anything you need from us to support you, please let us know.

Activities

Meetings will focus on relationship building, skill sharing, and project discussions. Once the data collection is completed, we will then turn our attention to creating educational materials to share the results of the research.

In addition to the research and skill sharing activities we want to incorporate fun activities or cultural activities such as beading, drum circles, bringing an Elder, playing games, sharing food together, music, etc.

Supplies
At each meeting:

- We will provide light lunches or snacks and drinks
- Cover any training and education costs
- Provide supplies for making educational materials
- Bring materials required for any activities as discussed

Compensation:

The peer group role will be compensated with an honorarium in the amount of $20/hour cash and bus tickets for transportation will be provided. Childcare will be compensated as needed $10 per hour. No money other than honorarium's will be received by peer members.

- ✓ **Confidentiality**- all team members will respect the confidentiality of other team members and any items discussed within group meetings.
- ✓ All members will declare any real or perceived conflict of interest at the outset of any meeting for management in accordance with the University of Manitoba *Conflicts of Interest Policy and Conflict of Interest Procedures.*
- ✓ All research project conducted within the University of Manitoba is governed by the *Responsible Conduct of Research Policy, Responsible Conduct of Research – Code of Research Ethics Policy, Responsible Conduct of Research – Investigation Procedure.*
- ✓ This research is funded by the Canadian Institute of Health Research; therefore, we follow and adhere to the *Tri-Agency Framework: Responsible Conduct of Research, including the framework and policies of Chapter 9: Research Involving the First Nations, Inuit, and Métis Peoples of Canada.*
