# Supplementary material for: Social and structural barriers and facilitators to HIV healthcare and harm reduction services for people experiencing syndemics in Manitoba: study protocol
Source: BMJ Open. 2023 Aug 2;13(8):e067813. doi: 10.1136/bmjopen-2022-067813 (PMC10401247; doi:10.1136/bmjopen-2022-067813)
Supplement: Supplementary data [file bmjopen-2022-067813supp003.pdf]

BMJ Publishing Group Limited (BMJ) disclaims all liability and responsibility arising from any reliance placed on this supplemental material which has been supplied by the author(s)


***Covid-19 Pandemic Concealing a Syndemic of Concern: Sex, Gender, Methamphetamine and Sexually Transmitted and Blood Borne Infections in People Living with HIV in Manitoba***

## Participant Interview Question Guide

| ***To be completed by facilitator*** | *Facilitator Name* | | |
|---|---|---|---|
| Participant ID: | | | |
| Participating Organization: | | | |
| Location: | | | |
| Interview date: | | | |
| Interview time: | Start | End | Duration |
| Confirmation of consent signed: | | | |

*Thank you for agreeing to speak with me today. My name is [INSERT] and my pronouns are INSERT] and this is my colleague [introduce themselves]. My role with this project is as a [Research Assistant, Research Associate, Research Coordinator]. Is there anything you need to do to prepare yourself before we get started? We anticipate the interview and short surveys will take between 1-2 hours to complete. We can take a break during the question portion of this interview if you need to, and we will take a short break after the interview before we begin the surveys. Please let me know at any time if you need to step away for a few minutes.*

*[NOTE: This next section only applies to people who will not have booked an interview appointment and are a walk-up participant]*

*Before I explain the purpose and what we will do today I want to check in with you about support we can offer you today and I have three questions I would like to ask you:*

1. ***Would you be interested in having a peer support to sit with you for your interview? The purpose of a peer support is only to provide emotional support and they will maintain confidentiality of anything we discuss today.***
2. ***Would you be interested in having cultural support? There is an Elder available who you can speak with at any time if you need a break during the interview, at the break between the interview and surveys or at the end of the interview.***



Rueda ZV, *et al. BMJ Open* 2023; 13:e067813. doi: 10.1136/bmjopen-2022-067813

3. ***Are you interested in speaking with a psychological support person at the break or after your interview today? They are available anytime if you need to stop the interview to debrief or check in with them.***

*Before we start the formal interview questions, I would like to remind you that we are here to speak about your experiences in finding and receiving HIV and STBBI-related health care and services. Our aim is to improve HIV and other services for everyone. We are interviewing people with many stories and, perhaps, some questions may not relate to your life experiences. We appreciate your responses since many of these questions are personal. There are some questions about substance use that may or may not apply to you. The reason we are asking these questions is because our research has shown that in Manitoba, the main way people get HIV is through injection drug use but that is by no means the only way people get HIV. We understand that some people may feel shame or stigma about having HIV or using drugs, which is why we have tried to create a safe and comfortable space to listen to your experiences. It is completely up to you if you want to answer the questions or not.*

*You have been asked to participate in this interview as you:*

1) *Are 18 years or older*
2) *Have received a diagnosis for HIV between 2018-2021*
3) *Are a resident of Manitoba*

*Do all of these statements apply to you?* ***Y / N***

*[If no, relay that they are not eligible to participate in the study. Thank them for their time, and end interview].*

*For the purpose of our discussion, STBBI services may include testing, care and counselling for HIV, Hepatitis C, syphilis, other sexually transmitted infections like Chlamydia, or gonorrhoea.*

*We take your information seriously which is why we also want to remind you that everything you share today will be kept confidential. This means that we will not disclose any information you share with us about drug use, engaging in sex work or anything else that could be perceived as "criminal" with authorities. The only reason we would ever have to break confidentiality is if there is a direct risk/danger to your health and safety or that of someone who is under 18. We would like to record this interview to make sure the information is accurate. This recording will be kept in a secure location and destroyed after this study is over. This recording will not be shared.*

*For your participation today, we will give you a $50 honorarium in the form of cash and bus fare or money to cover a taxi ride to attend the interview. Additionally, we have several items that you can choose from as a gift and a form of appreciation for the valuable insight you have shared with us today.*

***Covid-19 Pandemic Concealing a Syndemic of Concern: Sex, Gender, Methamphetamine and Sexually Transmitted and Blood Borne Infections in People Living with HIV in Manitoba***

*I would like to start with some questions about how things generally are for you, but before we start are there any questions you want to ask?*

1. **Could you please tell me about yourself?**
   a. PROBES
      i. How are things going for you right now?
      ii. How are you feeling coming in today?

2. **What does a typical week (day) look like for you?**
   a. PROBES
      i. Daily routine
      ii. Work/volunteer
      iii. Medical programs/services
      iv. Time with friends/family
      v. Time with caring for friends/family

3. **How are things going with your health right now?**
   a. PROBES- ask about:
      i. Physical health
      ii. Mental health
      iii. Emotional health
      iv. Spiritual health
      v. Financial health

*Now I would like for us to talk about the HIV care and other health services that you receive.*

4. **What was your experience when you were first diagnosed with HIV?**
   a. PROBES
      i. Any available support or counselling?
      ii. Were you given enough information about your options? (i.e. explained about your rights, how to access to care, follow up, linkage to doctor, treatment options?)
      iii. Did you learn about how HIV is transmitted? And how to prevent transmission?
      iv. Did you receive information about importance of treatment?



Rueda ZV, *et al. BMJ Open* 2023; 13:e067813. doi: 10.1136/bmjopen-2022-067813

5. **What health services do you currently receive for HIV health care?**
    a. PROBES,
        i. Attend clinic appointments, see a NP, take medication, regular blood testing, etc. (Linked to Care)
        ii. Alternative medicine such as traditional or cultural medicine
        iii. Anything else?
    b. **If not linked to care move to question 7**

6. **What do you like about the HIV health care services you receive?**
    a. PROBES
        i. Relationship with doctor/ nurse/ social worker
        ii. Accessibility of location to receive services
        iii. Welcoming environment at service provider location
        iv. Any other reason?

7. **What problems have you encountered when accessing HIV care?**
    a. PROBES
        I. Stigma, discrimination from service providers
            i. Drug use, unhoused, gender identity
            ii. If yes, please tell me more about that. Where does this discrimination occur?
        II. Transportation, accessibility challenges
        III. Lack of childcare to get to attend appointments or children are not able to accompany to appointment
        IV. Income, lack of money to pay for medication
        V. Other challenges are more important to deal with
        VI. Lack of access to appointments, lengthy wait times to receive care, etc.
        VII. Mental health challenges
        VIII. Any other things you don't like?

8. **Could you please walk me through a time when you needed HIV care but you could not get it?**
    a. PROBES
        i. Ask participant to share a specific experience related to a barrier
        ii. If you were diagnosed before the pandemic (2018-2019), did you experience similar or different challenges before or during the COVID-19 pandemic (2020-current)?

***Covid-19 Pandemic Concealing a Syndemic of Concern: Sex, Gender, Methamphetamine and Sexually Transmitted and Blood Borne Infections in People Living with HIV in Manitoba***

**9. Has the COVID-19 pandemic impacted the HIV health services you use? If yes, in what ways?**
   a. PROBES
      i. Availability of appointments, shortage of staff at clinics, services not accessible, staff changes, etc.
      ii. Increased fear/risk due to COVID-19 in health care settings
      iii. Transportation issues
      iv. Money, financial challenges
      v. Use of substances
      vi. Mental health challenges/and or lack of support for mental health

**10. How safe do you feel in or around health care settings?**
   a. PROBES
      i. In the neighborhood around the clinic/hospital
      ii. Around security personnel such as police, hospital security guards
      iii. Fear of Child & Family Services, removal of children, or fear of other government agencies/actors
      iv. Lack of privacy to discuss health status (i.e., ER visit)
      v. Not safe due to perceived gender identity, race/ethnicity
      vi. Getting to your appointment- location of clinic, transportation

**11. What are things that help you to get the health care you need?**
   a. PROBES
      i. Positive relationship with doctor/health professionals
      ii. Welcoming and supportive environment at health centres, health centres specific to my needs (i.e. gender specific, LGBTQ+)
      iii. Availability/hours
      iv. Location within the city
      v. Support (personal or peer) to attend health appointments

**12. What changes do you think would make it easier for you (and other people living with HIV) to access care?**
   a. PROBES
      i. Transportation support, financial support
      ii. Peer support (i.e., someone to accompany you to appointments)
      iii. Better accessibility (e.g., different sites for treatments, availability of appointments, options for care)
      iv. Access to counselling and/or mental health support
      v. Addressing stigma
      vi. Different medication with less side effects
      vii. Access to material support (housing, food, etc.)
      viii. Bundling more services in one appointment- seeing multiple professional, etc. primary care physician, gynaecologist, mental health professional,



Rueda ZV, *et al. BMJ Open* 2023; 13:e067813. doi: 10.1136/bmjopen-2022-067813

ix. Addressing multiple health concerns at each appointment- either related to HIV or other health issue

*I have a few more questions before we end the interview, they are related to substances you may use. These questions may not apply to you, and we want to assure you that there is no judgment based on your use of substances. These questions will help us find ways to better support people who are living with HIV who also may use substances. I also want to remind you that you do not have to answer any questions you do not feel comfortable with, and that all of your answers are confidential.*

**13. Do you have any experience with using substance?**
**IF YES, ask the following questions. If no, move to QUESTION 18**

**14. How old were you when you started using substances?**
- a. PROBE:
  - i. What substance(s) did you start with?

**15. How would you describe your substance use when you started?**
- a. PROBES:
  - i. Heavy use (one or more times per day)
  - ii. Moderate use (few times per week)
  - iii. Light use (once a week or a few times a month)
  - iv. Very light (less than once a month)

**16. What type of substances do you currently use?**
- a. PROBES
  - i. If crystal meth- do you inject or smoke or both?
  - ii. If opioids- inject or smoke, or pill?
- b. **If YES to injection ask question 18, if NO skip question 18**

**17. How would you describe your current substance use?**
- a. PROBES
  - i. Several times per day (one or more)
  - ii. Few times a week
  - iii. Once a week or a few times a month
  - i. Less than once a month

**18. How do you access the substances you use?**
- a. PROBE
  - i. Do you get it yourself or does someone else get them for you?)

*This next series of questions we are interested in better understanding the practices you use to inject drugs.*

***Covid-19 Pandemic Concealing a Syndemic of Concern: Sex, Gender, Methamphetamine and Sexually Transmitted and Blood Borne Infections in People Living with HIV in Manitoba***

19. **If you use substances that you inject, how often do you:**
    a. **Use a new needle, spoon, sterile water or filter?**
        i. All of the time, some of the time, rarely or never?
        ii. If only some of the time or never- what are the barriers to accessing safe injection supplies?

    b. **Inject with a needle or syringe used by somebody else?**
        i. All of the time, some of the time, rarely, or never?

    c. **Clean and disinfect the injection site?**
        i. All of the time, some of the time, rarely, or never?

    d. **Prepare using a spoon, water, or filter used by somebody else?**
        i. All of the time, some of the time, rarely, or never?

    e. **Have a safe place to use?**
        i. All of the time, some of the time, rarely, or never?
        ii. If yes, what makes this place safe?
        iii. If no, what makes this place unsafe?

20. **Do you know where to find harm reductions supplies?**
    a. PROBE
        i. If yes, where do you get them from?
            1. What supplies do you get?
            2. How often do you get harm reduction supplies?

21. **Have you experienced any violence related to your substance use?**
    a. PROBE
        i. If yes, and you feel comfortable, can you please tell me what happened?

22. **What would make using substances safer for you?**
    a. PROBE
        i. Better access to harm reduction supplies
        ii. Safe injection site
        iii. Decriminalization of drug use
        iv. Safe person to use drugs with

23. **Does using substances affect your ability to access HIV care & services?**
    a. PROBES
        i. If yes, in what ways?



Rueda ZV, *et al. BMJ Open* 2023; 13:e067813. doi: 10.1136/bmjopen-2022-067813

***Covid-19 Pandemic Concealing a Syndemic of Concern: Sex, Gender, Methamphetamine and Sexually Transmitted and Blood Borne Infections in People Living with HIV in Manitoba***

**24. How did the COVID-19 pandemic impact your substance use?**
   a. PROBE
      i. What changes in your pattern of use- availability, safety of substance, harm reduction supplies?
      ii. Changes to type of substance used or ways of using- did you use more of one substance or start injecting instead of smoking?

**25. What do you think is the best way [how do you want] to learn about prevention and treatment of STBBI's and harm reduction practices?**

**26. Is there anything else you want to tell us today?**

*That is the end of the interview portion of our time together today.*

*<u>Thank you very much for speaking with me.</u> I really appreciate you taking the time today to do this. I know some of these questions were very personal and might have brought up some emotions for you. If you need support after our chat, I can provide you a list of resources* ***[give participant mental health support resource document].*** *[Insert name of counsellor or Elder] is available to speak with you now if you would like that support. You can also speak to [Participating Organization] and they can direct you to any other supports you can access.*

*Now that we have completed the interview portion of the interview, we will take a short break* ***[discuss with the participant what they need to do- offer Elder support, food, break to walk around, smudge ceremony, stretch/move their body, etc.].*** *When we come back together, I will ask you to please fill out three short surveys. This should take another 30-45 minutes to complete. After that if there are things, you want to add to this discussion, we will make sure you have enough time to do that.*



Rueda ZV, *et al. BMJ Open* 2023; 13:e067813. doi: 10.1136/bmjopen-2022-067813
