# Supplementary material for: Social and structural barriers and facilitators to HIV healthcare and harm reduction services for people experiencing syndemics in Manitoba: study protocol
Source: BMJ Open. 2023 Aug 2;13(8):e067813. doi: 10.1136/bmjopen-2022-067813 (PMC10401247; doi:10.1136/bmjopen-2022-067813)
Supplement: Supplementary data [file bmjopen-2022-067813supp004.pdf]

Participant Survey: Demographics & Life Circumstances

The following short questionnaire asks questions about you, such as your age, identity, where you live, your experiences with substance use, your sexual practices, and other life circumstances you may have encountered. This information is very helpful as it provides a better understanding of your experiences as a person living with HIV. We understand that these questions are very personal and intimate, and we appreciate your responses. **There is no judgement about anything you share with us**. Every person has different life experiences and situations that affect their health and well-being. Some of these questions may not apply to you, **please only answer what is relevant to your life**.

Please **circle the answer(s) that applies to you, or write your answer in the space provided. If there are questions you do not feel comfortable answering, please select "Prefer not to say".** Please ask your interviewer if you would like them to better explain any of the questions.

Thank you for taking part in our survey!

PART A: Demographics

1. What is your date of birth? Day: ______/Month: ______/Year: _______

2. How old are you?

____

3. What gender identity best describes you?
   a. Woman
   b. Man
   c. Transgender woman
   d. Transgender man
   e. Non-binary (agender, genderfluid, pangender, genderqueer, gender nonconforming, etc.)
   f. Two-Spirit
   g. Other
      Which? ______________________
   h. Prefer not to say

Rueda ZV, *et al. BMJ Open* 2023; 13:e067813. doi: 10.1136/bmjopen-2022-067813

4. What sex were you assigned at birth? (as shown on your original birth certificate)
   a. Female
   b. Male
   c. Intersex
   d. Prefer not to say

5. What sexual orientation best describes you?
(Please select all that apply to you)

   a. Lesbian
   b. Gay
   c. Bisexual
   d. Asexual
   e. Heterosexual/Straight
   f. Pansexual
   g. Other
      Which?______________________
Prefer not to say

6. What cultural background best describes you?
(Please select all that apply to you)
   a. Indigenous
      i. First Nations
      ii. Métis
      iii. Inuk
      iv. Other ______________________
      v. Unspecified ______________________
   b. African
   c. White/European
   d. East/Southeast Asian
   e. South Asian
   f. South East Asian
   g. Hispanic or Latinx
   h. Middle Eastern
   i. Other (please specify) ________________________
   j. Prefer not to say

Rueda ZV, *et al. BMJ Open* 2023; 13:e067813. doi: 10.1136/bmjopen-2022-067813

7. What is your marital status?

a. Single
b. Married
c. Divorced
d. Common-law
e. Widowed
f. Other
   Which_______________________________
g. Prefer not to say

8. What languages do you speak? **(Please select all that apply to you)**

Languages	Yes	No	Which (please specify) *
English			
French			
Cree			
Dakota			
Dene			
Inuktitut			
Michif			
Ojibway			
Oji-Cree			
Filipino			
Spanish			
German			
Ukrainian			
Punjabi			
Other (please specify)			
Prefer not to say			

*It applies just for "Other" option answers

Rueda ZV, *et al. BMJ Open* 2023; 13:e067813. doi: 10.1136/bmjopen-2022-067813

9. What religion best describes you? **(Please select all that apply to you)**

a. Traditional Indigenous
b. Christianity
    i. Catholicism
    ii. Witness of Jehovah
    iii. Evangelical
    iv. Protestant
    v. Orthodox
c. Buddhism
d. Hindu
e. Jewish
f. Muslim
g. Sikh
h. Other Religion___________________________
i. No religious affiliation
j. Prefer not to say

10. What is the highest level of education you have completed?
a. K-12
    Which was the last grade? _______________
b. Certificate, diploma, vocational course from an educational institution
c. Bachelor's degree
d. Master's degree
e. Doctorate
f. Other, which ___________________________
g. Prefer not to say

11. Where do you reside? (City/Town/Rural Community/Reserve)

a. ____

Rueda ZV, *et al. BMJ Open* 2023; 13:e067813. doi: 10.1136/bmjopen-2022-067813

12. The following question are about **difficulties you may have doing certain activities**. Please tell us only about problems that **have lasted or are expected to last for six months or more**. Mark your response with an **X** for each difficulty.

Difficulties	No	Sometimes	Often	Always	Prefer not to say
Seeing					
Hearing					
Walking, using stairs, using hands or finger or doing other physical activities					
Learning, remembering, or concentrating					
Any emotional, psychological, or mental health conditions? (Anxiety, bipolar disorder, substance use, anorexia, etc)					

PART B. Income and Housing

Now we will ask you some questions related to your income and housing. These answers will allow us to better understand the resources that people living with HIV may need to support their health and well-being.

13. Are you currently employed?
   a. Yes
   b. No
   c. Prefer not to say

14. What is your main source or source(s) of income?

____

15. Which of the following is your approximate yearly income?
   a. less than 10,000 CAD/year
   b. 10,000 – 19,999CAD/year
   c. 20,000 - 29,999 CAD/year
   d. 30,000 - 39,999 CAD/year
   e. 40,000 - 49,999 CAD/year
   f. More than 50,000/year
   g. Prefer not to say

Rueda ZV, *et al. BMJ Open* 2023; 13:e067813. doi: 10.1136/bmjopen-2022-067813

16. Please mark with an **X** any items that you currently **have or do not** have enough income to cover. **(Please select all that apply to you)**

Items	Have Enough	Do not have Enough	Prefer not to say	Does not apply
Housing (rent, hydro, water)				
Transportation				
Food				
Clothing				
Childcare				
Pet Care				
Healthcare				
Other (please specify)________________				

17. What is your current living situation? **(Please select all that apply to you)**

Living situation	Yes	No	Which (please specify) *
Living alone (house/apartment)			
Living with partner (married, common-law or relationship)			
Living with children			
Living with roommates			
Living with extended family members			
Experiencing housing instability (insecure housing, shelter, transitional housing, houseless)			
Other (please specify)			
Prefer not to say			

*It applies just for "Other" option answers

Questions 17, 18, and 19 only apply to people who are experiencing housing instability.

18. How long have you experienced housing instability?

________ ____________ ___________

Days Months Years

19. **If you are experiencing housing instability,** which of the following are you experiencing? **(Please select all that apply to you)**

Living situation	Yes	No	Which (please specify) *
Insecure housing (month to month, threats by landlord to leave, unsure how you will pay rent next month)			
Couch surfing			
Sleeping rough (sleeping in the streets)			
Staying at a shelter			
Short-term housing program			
24/7 space			
Other (please specify)			
Prefer not to say			

*It applies just for "Other" option answers

20. **If you are experiencing housing instability,** did you experience housing instability before or after being diagnosed with HIV?
    a. Before I was diagnosed
    b. After I was diagnosed
    c. Before and after I was diagnosed
    d. Other
       Which?____________________
    e. Prefer not to say

PART C: Criminal Justice System

The next few questions we want to ask you are related to your experiences with the criminal justice system. We are asking these questions as recent HIV infections have shown a number of infections in people who were incarcerated. These questions will help us to also understand how policing and surveillance may be affecting your life and health. We do not judge any of your answers and we are not affiliated in any way with police or the justice system.

Rueda ZV, *et al. BMJ Open* 2023; 13:e067813. doi: 10.1136/bmjopen-2022-067813

21. Have you ever been incarcerated? (prison, jail, correctional facility)

	Initial date (from)	Final date (to)
Yes	Month: ________/Year: ________	Month: ________/Year:________
	Month: ________/Year: ________	Month: ________/Year:________
	Month: ________/Year: ________	Month: ________/Year:________
No	(Skip to Part D)	
Prefer not to say		

22. Did you receive an <u>HIV positive diagnosis while incarcerated</u>?
  a. Yes
  b. No **(Skip to question 23)**
  c. Prefer not to say

23. **If you did receive a positive diagnosis while incarcerated**, who told you about your diagnosis? **(Please select all that apply to you)**
  a. Physician
  b. Nurse
  c. Administration staff
  d. Prison Guard
  e. Other___________________
  f. Prefer not to say

24. Did you experience <u>discrimination</u> while incarcerated <u>because of your HIV diagnosis?</u>
  a. Yes
  b. No **(Skip to question 25)**
  c. Prefer not to say

Rueda ZV, *et al. BMJ Open* 2023; 13:e067813. doi: 10.1136/bmjopen-2022-067813

25. If you did experience discrimination because of your HIV diagnosis, who did you experience discrimination from? **(Please select all that apply to you)**

	Yes	No	Who (please specify) *
Other incarcerated people			
Prison guards			
Healthcare staff			
Administration staff			
Child and Family Services (CFS)			
Other (please specify)			
Prefer not to say			

*It applies just for "Other" option answers

26. Have your experiences with the criminal justice system affected your ability to access HIV care and other health care services after you were released?

a. Yes
b. No
c. Prefer not to say

27. Please select with an **X** the services you had access to while incarcerated

Services	Had Access	Did not have access
Primary health care services (regular physicals, doctor appointments)		
HIV related care (medication, regular blood testing, specialist appointments)		
STBBI testing		
Harm Reduction Supplies (condoms, syringes, pipes)		
Child and Family Services (CFS)		
Mental health services and counselling		
Ceremony		

*Sexually Transmitted and Blood Borne Infections

28. Are you currently under correctional supervision (e.g., bail conditions, probation, or parole)?

	Start date (from)	Final date (to)
Yes	Month: ________/Year: ________	Month: ________/Year:________
No	**(Skip to Part D)**	
Prefer not to say		

PART D: Sexual Practices

Now we would like to ask you some questions about your sexual practices. Please answer what you are comfortable with. We are asking these questions as people can acquire HIV through sexual activity but that is not the only way. The reason we are asking these questions is to help us better understand the sexual practices of people living with HIV which can be useful when informing safe sex education.

29. Are you currently sexually active?

a. Yes
b. No
c. Prefer not to say

30. In the 12 months have you had sex with **(Please select all that apply to you)**

	Yes	No	Which (please specify) *
Women			
Men and women			
Men			
Non-binary person			
Two-spirit person			
Other (please specify)			
Prefer not to say			

*It applies just for "Other" option answers

31. In the past 12 months how many sexual partners have you had?

a. ________________
b. Prefer not to say

32. When engaging in sexual activity, do you use any of the following safe sex protections?

Protections	All of the time	Some of the time	Never
Condoms			
Dental dams			
Pre-exposure prophylaxis (Prep)			
Post exposure prophylaxis (Pep)			
Regular STBBI testing			
Other (please specify)________________			

33. How easy was it to get safe-sex protections before and during the COVID-19 pandemic?

a. Easier before COVID-19 pandemic
b. No difference before and during COVID-19 pandemic
c. Easier during COVID-19 pandemic
d. Prefer not to say

34. Do you use a form of contraception? (Birth control, condoms)

a. Yes
b. No **(Skip to questions 36)**
c. Prefer not to say

35. Mark with an **X** your use of the following forms of contraception:

Contraception	Yes	No	Which (please specify)
Short-acting hormonal methods (oral birth control)			
Long-acting reversable contraceptive (IUD)			
Barrier Methods (condom, sponge, cervical cap)			
Natural rhythm methods			
Sterilization (tubal ligation or vasectomy)			
Other (please specify)			
Prefer not to say			

36. How often do you get blood tests for sexually transmitted infections and blood born infections (Hep C, Syphilis, Gonorrhea)?

a. Before engaging in sexual activity with a new partner
b. Once a month
c. Every few months
d. Once a year
e. When my doctor/nurse practitioner recommends
f. Never
g. Other
When (please specify)?________________________________
h. Prefer not to say

PART E: Substance Use

We would now like to ask you about your substance use. These questions may or may not apply to you, and we want to assure you that there is no judgment based on your use of substances. We are asking these questions to understand how to better support people who are living with HIV who also may use substances

37. Do you currently use substances? (alcohol, marijuana, crystal meth, crack, cocaine)

a. Yes
b. No
c. Prefer not to say

38. Please fill in this chart. For every substance please select with an (x) the quantity and length of consumption

Substance	Consumption			Quantity Consumed					Time of consumption			
	Never	Past	Present	Quantity	Daily	Weekly	Monthly	Yearly	Length	Days	Months	Years
Tobacco				# of cigarettes								
Alcohol				# of drinks								
Inhaled Substances				# of inhalations								
Smoked Substances				# of times smoking								

Injected Substances				# of injections								

39. How old were you when you started using substances?

____
Age

40. What substance did you start with?

____
Name(s) of substance

41. Please describe your current and past substance use by using an **X** in every line

	Heavy use (one or more times per day)	Moderate use (few times per week)	Light use	Very light use (once a week or a few times a month)	Not using
Current substance use					
Substance use when you started					

42. Does substance use impact your sexual practices in any of the following ways, select with an **X** if yes or no:

**Impacts**	**Yes**	**No**	**Prefer not to say**
I am **more likely** to engage in sex with multiple partners when using substances			
I am **less likely** to engage in sex with multiple partners when using substances			
I am **less likely** to use protection when engaging in sexual activity when using substances			
I am **more likely** to use protection when engaging in sexual activity when using substances			
Other, which			

Rueda ZV, *et al. BMJ Open* 2023; 13:e067813. doi: 10.1136/bmjopen-2022-067813

43. If you use substances that you inject, **how often do you**:

Practices	All of the time	Some of the time	Not very often	Never
Use a new needle				
inject with a needle or syringe used by somebody else				
Bleach or clean needles used by somebody else				
Prepare using a spoon, water, or filter used by somebody else				
Have someone else inject your substances				
Have a safe place to inject				
Know where to find harm reduction supplies				

PART F: Experiences with Violence

For the last portion of this survey, we want to better understand your experiences with violence. This section may or may not apply to you. The reason we are asking these questions is because during the COVID-19 pandemic there was a reported increase in experiences of violence, in particular intimate partner violence and domestic violence. There is also a higher rate of women who have been diagnosed with HIV in Manitoba over the past few years – and women, transgender, non-binary people, as well as those who identify as Indigenous and 2SLGBTQQIA+ are more likely to experience all forms of violence.

Rueda ZV, *et al. BMJ Open* 2023; 13:e067813. doi: 10.1136/bmjopen-2022-067813

44. Throughout your life, have you ever experienced any of the following types of violence? **(Please select all that apply to you)**

Types of Violence	Any point in your life	Past 3 years	Currently
Physical violence			
Sexual violence			
Stalking, harassment			
Emotional abuse			
Intimate Partner Violence (violence perpetrated by a partner in a romantic or dating relationship)			
Domestic Violence (violence among people in a domestic situation- can include any type of family, or roommate)			
Cyberstalking/cyberbullying			
Child abuse			
Psychological / mental violence			
Other, which			
Prefer not to say			

45. If you are currently experiencing Intimate Partner Violence, mark with an **X** the ways it impacts you:

**Impacts**	**Yes**	**No**	**Prefer not to say**
I am not able to attend HIV clinic appointments or HIV related care because of my partner (Partner controls my time and schedule, money for health care costs, etc.)			
I am afraid of my partner finding out about when I am going to doctor appointments or taking medication			
My health care is not impacted, and I am able to attend all my appointments			
Other, which			

46. Have you received any support or health care services to support you with healing from experiences of violence (trauma counselling, psychological support, traditional healing)?

a. Yes
b. No **(Skip to question 48)**
c. Prefer not to say

47. If you have received support following your experiences with violence, which of the following have you received? **(Please select all that apply to you)**

Supports	Yes	No	Which (please specify) *
Counselling (Trauma counselling, Domestic Violence counselling)			
Psychological Support			
Support groups			
Traditional healing			
Domestic Violence shelter			
Women's centre			
Other (please specify)			
Prefer not to say			

*It applies just for "Other" option answers

48. Has anyone shared your HIV status without your consent?
a. Yes
b. No
c. Prefer not to say

That brings us to the end of the survey. Thank you again for taking the time to fill out this survey. We really appreciate your time and willingness to share your experiences with us. If you have any questions or would like to talk more with the interviewer, or support person, that can happen now. We have also created a resource sheet to access support or counselling if you would like a copy.
