# Supplementary material for: Social and structural barriers and facilitators to HIV healthcare and harm reduction services for people experiencing syndemics in Manitoba: study protocol
Source: BMJ Open. 2023 Aug 2;13(8):e067813. doi: 10.1136/bmjopen-2022-067813 (PMC10401247; doi:10.1136/bmjopen-2022-067813)
Supplement: Author's manuscript [file bmjopen-2022-067813.draft_revisions.pdf]

BMJ Open is committed to open peer review. As part of this commitment we make the peer review history of every article we publish publicly available.

When an article is published we post the peer reviewers' comments and the authors' responses online. We also post the versions of the paper that were used during peer review. These are the versions that the peer review comments apply to.

The versions of the paper that follow are the versions that were submitted during the peer review process. They are not the versions of record or the final published versions. They should not be cited or distributed as the published version of this manuscript.

BMJ Open is an open access journal and the full, final, typeset and author-corrected version of record of the manuscript is available on our site with no access controls, subscription charges or pay-per-view fees (http://bmjopen.bmj.com).

If you have any questions on BMJ Open's open peer review process please email info.bmjopen@bmj.com

BMJ Open

Gendered syndemics of HIV during Covid-19: protocol for a mixed methods study.

Journal:	*BMJ Open*
Manuscript ID	bmjopen-2022-067813
Article Type:	Protocol
Date Submitted by the Author:	30-Aug-2022
Complete List of Authors:	Rueda, Zulma; University of Manitoba Faculty of Health Sciences, Department of Medical Microbiology and Infectious Diseases<br>Haworth-Brockman, Margaret; National Collaborating Centre for Infectious Diseases; University of Manitoba, Department of Community Health Sciences<br>Sobie, Cheryl; University of Manitoba Faculty of Health Sciences, Department of Medical Microbiology and Infectious Diseases<br>Villacis, Enrique; University of Manitoba Faculty of Health Sciences, Department of Medical Microbiology and Infectious Diseases<br>Larcombe, Linda; University of Manitoba Faculty of Health Sciences, Department of Internal Medicine<br>Maier, Katharina; The University of Winnipeg, Criminal Justice<br>Deering, Kathleen; The University of British Columbia, Department of Medicine; Centre for Gender & Sexual Health Equity<br>Sanguins, Julianne; Manitoba Metis Federation; University of Manitoba, Department of Community Health Sciences<br>Templeton, Kimberly; Manitoba HIV Program; Nine Circles Community Health Centre<br>MacKenzie, Lauren; Manitoba HIV Program; University of Manitoba Faculty of Health Sciences, Department of Internal Medicine<br>Ireland, Laurie; Nine Circles Community Health Centre; Manitoba HIV Program<br>Kasper, Ken; University of Manitoba, Internal Medicine; Manitoba HIV Program<br>Payne, Michael; Nine Circles Community Health Centre<br>Bullard, Jared; University of Manitoba, Medical Microbiology and Infectious Diseases; Cadham Provincial Laboratory<br>Krusi, Andrea; The University of British Columbia, Department of Medicine; Centre for Gender & Sexual Health Equity<br>Pick, Neora; Oak Tree Clinic; The University of British Columbia, Division of Infectious Disease<br>Myran, Tara; University of Winnipeg, Indigenous Development<br>Meyers, Adrienne; JC Wilt Infectious Diseases Research Centre, National STBBI Laboratory Division for Underserved Populations<br>Keynan, Yoav; University of Manitoba Faculty of Health Sciences, Internal Medicine; National Collaborating Centre for Infectious Diseases
Keywords:	COVID-19, HIV & AIDS < INFECTIOUS DISEASES, QUALITATIVE RESEARCH

BMJ Open Submission

Title: Gendered syndemics of HIV during Covid-19: protocol for a mixed methods study.

Authors:

Zulma Vanessa Rueda M.D., Ph.D.[1*ǂ], Margaret Haworth-Brockman M.Sc.[2,3ǂ], Cheryl Sobie M.A.[1], Enrique Villacis M.A.[1], Linda Larcombe Ph.D.[4], Katharina Maier Ph.D.[5], Kathleen Deering Ph.D.[6,7], Julianne Sanguins Ph.D.[3,8], Kimberly Templeton M.Sc.[9,10], Lauren MacKenzie M.D., MPH. [4,9], Laurie Ireland M.D.[9,10], Ken Kasper M.D.[4,9], Michael Payne B.A.[9,10], Jared Bullard M.D.[1,11], Andrea Krusi Ph.D.[6,7], Neora Pick M.D.[12,13], Tara Myran B.A.[14], Adrienne Meyers M.Sc. Ph.D.[15], Yoav Keynan M.D., P.h.D.[1,2,3,4,9ǂ]

[1] Department of Medical Microbiology and Infectious Diseases, University of Manitoba Rady Faculty of Health Sciences, Winnipeg, MB, R3E 0J9, Canada

[2] National Collaborating Centre for Infectious Diseases, Rady Faculty of Health Sciences, University of Manitoba, Winnipeg, MB, R3E 0T5, Canada

[3] Department of Community Health Sciences, University of Manitoba, Rady Faculty of Health Sciences, Winnipeg, MB, R3E 0J9, Canada

[4] Department of Internal Medicine, University of Manitoba Rady Faculty of Health Sciences, Winnipeg, MB, R3E 0J9, Canada

[5]Criminal Justice, The University of Winnipeg, Winnipeg, Canada

[6]Division of Social Medicine, Department of Medicine, Faculty of Medicine, The University of British Columbia, Vancouver, Canada

[7]Centre for Gender & Sexual Health Equity, Vancouver, Canada

[8] Manitoba Metis Federation, Winnipeg, Canada

[9]The Manitoba HIV Program, Winnipeg, Canada

For peer review only - http://bmjopen.bmj.com/site/about/guidelines.xhtml

[10] Nine Circles Community Health Centre, Winnipeg, Canada

[11] Cadham Provincial Laboratory, Winnipeg, Canada

[12] Oak Tree Clinic, BC Women's Hospital, Vancouver, Canada

[13] Division of Infectious Disease, University of British Columbia, Vancouver, Canada

[14] Indigenous Development, University of Winnipeg, Winnipeg, Canada

[15] National STBBI Laboratory Division for Underserved Populations, Public Health Agency of Canada, JC Wilt Infectious Diseases Research Centre, Winnipeg, Manitoba

[Ł] Principal investigators

***Correspondence**: Zulma Vanessa Rueda. Email: zulma.rueda@umanitoba.ca

For peer review only

For peer review only - http://bmjopen.bmj.com/site/about/guidelines.xhtml

Abstract

Introduction:

In Manitoba, Canada, injection drug use has become the most common mode of HIV transmission since 2018. Among persons living with HIV (PLHIV), half are women. Across Canada, public health measures during the Covid-19 pandemic have increased sex and gender disparities, decreased capacity to distribute harm reduction services, and reduced access to primary care and general health care. The project's goal is to explore the sex- and gender-related programmatic and structural barriers in harm reduction and health care services, as well as individual facilitators associated with access, and linkage to care for women, men and non-binary persons living with HIV (PLHIV), before and during Covid-19, in both those who do and do not inject drugs. This paper describes the novel protocols that will be used for this study.

Methods and analysis:

Our framework of inquiry is intersectional and uses participatory action research methodology and mixed methods, including open-ended interviews and surveys. Semi-structured face-to-face interviews and questionnaires will be conducted with approximately 40-60 individuals from two groups: (1) Participants who were diagnosed with HIV between 2018 and 2021 and; (2) Clinic program providers and social service employees who work with PLHIV. Guiding the methodological approach are the first-hand experiences and knowledge of PLHIV. To analyze our data, we will employ a sex- and gender-based analysis and utilize a socio-ecological framework to describe facilitators and barriers to care for PLHIV in Manitoba.

Ethics and dissemination:

For peer review only - http://bmjopen.bmj.com/site/about/guidelines.xhtml

This project was approved by the University of Manitoba Health Ethics Research Board (HS25572; H2022:218). Findings will be disseminated employing community-focused knowledge translation strategies identified by participants, peers, and community members and organizations. Results will be presented to the Manitoba HIV Program, the study's Research Advisory Committee and Peer Research Team, and provincial and national committees, in conferences, peer-reviewed journal articles, social media, workshops, and a dedicated webpage.

Keywords: HIV care, HIV barriers and facilitators, injection drug use, participatory action research, Covid-19.

Strengths and Limitations

- This study employs a replicable model of community-engaged Participatory Action Research. Community organizations, community members, and people with lived experience/peers have shared their knowledge and expertise to inform the project design. A diverse research advisory committee and a peer-led research team have been convened to ensure that this research accurately captures people's experiences and produces tangible outcomes that benefit the community.
- First Nations and Métis academic and community experts are actively participating in the research process to ensure that the study is culturally safe and aligned with the needs of Indigenous communities and people with lived experience.
- The use of in-depth semi-structured interviews and questionnaires will provide qualitative and quantitative insights on barriers of access and retention to health care in PLHIV who inject drugs, compared to those who do not inject drugs, before and during the Covid-19 pandemic. In addition, these methods will ensure replicability and provide a space for a deep and enriched understanding of people's experiences while reaching data saturation.

For peer review only - http://bmjopen.bmj.com/site/about/guidelines.xhtml

- The sample may be biased since interviews will primarily occur in two metropolitan areas, and with individuals who can dedicate time for the research process. While efforts will be made to reach participants who are not currently linked to care, there are individuals who, due to challenging life circumstances and structural barriers, may not be included in the sample. However, this prospective study will illustrate where more work is needed to dismantle the barriers related to reach people who most need HIV services and care, as well as identify the successful approaches that can be replicated among HIV care providers in Canada and beyond.

Word Count: 3953

For peer review only

For peer review only - http://bmjopen.bmj.com/site/about/guidelines.xhtml

Introduction

Approximately 0.5% (36.9 million) of the world's population live with Human Immunodeficiency Virus (HIV) [1,2]. Despite the proliferation of global and local initiatives to address HIV infections [2–5], recent findings suggest a resurgence of cases among North and South American and European countries [1]. Mechanisms of HIV transmission include blood transfusion, vertical transmission, sexual intercourse, and drug-related exposures [6]. In Canada, HIV transmission has steadily increased among women and people who inject drugs (PWID) [7,8]. Among PWID, methamphetamines in particular account for new infections and community transmissions [9,10].

The Covid-19 pandemic has dramatically impacted the health of many individuals, but people living with HIV (PLHIV) are especially vulnerable [11]. In particular, the reduction or complete closure of HIV treatment centres and services in some jurisdictions put the cascade of care for PLHIV at risk of breaking down [11]. The Public Health Agency of Canada conducted an online survey among people over 18 who identified themselves as PWID in the past six months to understand how Sexually Transmitted or Blood Borne Infections (STBBIs) and harm reduction services have changed during the Covid-19 pandemic [12]. The survey results showed that 52% of respondents increased their personal use of methamphetamine, during the pandemic, and more than half increased their use of alcohol and other drugs [12]. The survey found that 50% of PLHIV had challenges getting to a provider or clinic since the pandemic started (e.g., difficulty getting appointments, reduced hours of operation) [12]. Similarly, more than half of the participants had difficulties getting STBBI-related services, needle and syringe distribution programs, and naloxone training [12]. Data on this survey were not reported by sex or gender, which hinders a sex- and gender-based analysis. Likewise, the study's online modality may not have reached PWID who are unstably housed or without internet access, thus likely

For peer review only - http://bmjopen.bmj.com/site/about/guidelines.xhtml

underestimating the disruption of services. This survey provided initial exploratory findings on how the Covid-19 pandemic has impacted the lives of PLHIV and PWID. However, more research is needed to understand specific service limitations to develop strategies to address them.

In Manitoba, Canada, injection drug use is the most common mode of HIV transmission since 2018 [9]. The main drug injected in females and males is methamphetamines [9], a stimulant with a long half-life, resulting in significant behavioral impacts and for which replacement therapy is unavailable [13]. There is an overrepresentation of women who inject drugs among people newly diagnosed with HIV in Manitoba [9]. A convergence of socio-economic factors, mental health concerns, violence and exploitation, is driving a syndemic (i.e., concurrence of biological, social, behavioral, structural and other adverse factors that promote the clustering of multiple diseases [14]) of new HIV and other STBBI cases, with disproportionate representation of women who inject drugs [9]. Increased methamphetamines use and the repercussions of the Covid-19 pandemic threaten to undo decades of advances in preventing and treating HIV and other STBBIs in Manitoba.

The care continuum for PLHIV is comprised of several interconnected components (e.g., diagnosis, linkage to care) [15,16]. Linkage to care has been associated with positive health outcomes and reduced HIV mortality [16,17]. In a systematic qualitative review, Tso and colleagues [16] found that interventions focused on increasing community participation and literacy, creating community outreach mobile teams, integrating HIV-specific services in primary care, and providing substance use cessation services effectively improved linkage to care [16]. On the other hand, lack of information, anxiety, fear, and stigma after an HIV diagnosis were barriers to linkage to care [16]. Moreover, Hall and researchers [18] found the

For peer review only - http://bmjopen.bmj.com/site/about/guidelines.xhtml

use of community health workers and lay health workers, incorporating technology with health (e.g., medication reminders through phone applications), support from friend and family, access to intensive case management, and positive interpersonal relationships with service providers among leading reasons for retaining people in HIV care [18]. Personal and community stigma and discrimination, fear of HIV disclosure, and service provider shortages were among the leading barriers to PLHIV staying in care [18]. These studies suggest possible facilitators of and barriers to linking and retaining people to HIV care. Nevertheless, more research is needed to understand how these structural factors have been affected due to the Covid-19 pandemic, especially for underserved groups.

There is also an emerging pattern of persons with newly diagnosed HIV not routinely returning for regular care and treatment, reversing a 20-year trend of continuous follow-up care, which leads to considerable loss of follow-up appointments [9,12]. This emerging trend suggests a decline in progress towards the UNAIDS 95-95-95 goal (i.e., 95% of all PLHIV know their status, 95% of those diagnosed receive antiretroviral treatment, and 95% of those on treatment achieve viral suppression) [19].

Similarly, previous findings have highlighted how HIV experiences are shaped by biological sex and gender identities [10,20–22]. For instance, Ostrach and Singer [22] emphasize the importance of biological, social, and political factors that place women at increased risk of HIV infection and suggest integrating sex- and gender-based analyses in HIV research to capture the impact of sex and gender on PLHIV accurately. Similarly, a study based in Vancouver sought to understand violence and trauma among women (i.e., cis and transwomen) who participate in sex work, live with HIV and may or may not inject methamphetamines [10]. The authors found that a majority of women (61%) had experienced physical or sexual abuse during

For peer review only - http://bmjopen.bmj.com/site/about/guidelines.xhtml

childhood and adolescence, and those who initiated methamphetamines use were more likely to have experienced childhood abuse [10]. These findings suggest that gender, sex, and violence affect the experiences of PLHIV. However, more research is needed to understand how gender and other factors (e.g., unstable housing, substance use, violence) intersect to affect the HIV services of PLHIV during the Covid-19 pandemic.

This paper provides an overview of the protocol for a mixed methods study. Semi-structured interviews and surveys will be conducted to provide an in-depth understanding of how the Covid-19 pandemic has impacted access and retention to HIV and other STBBI-related care for persons living with HIV in Manitoba who may or may not use drugs.

Study Objectives and Outcomes

This study explores the sex- and gender-related, programmatic, structural barriers, and service gaps, as well as individual-level resilience factors associated with access to care and non-linkage to care in women, men, and non-binary persons living with HIV, before and during Covid-19, in both those who do and who do not inject drugs. PLHIV and staff that support and provide services to PLHIV (e.g., clinicians, nurses, social workers, program organizers) will be interviewed for this study.

It is important to note that this study is one component of a more extensive study aimed at understanding the epidemiology, risk, barriers and facilitators associated with HIV acquisition and care in Manitoba.

Outcomes

1) To gain an in-depth understanding about the gendered social, structural and programmatic barriers and gaps in services that prevent women, men and non-binary

For peer review only - http://bmjopen.bmj.com/site/about/guidelines.xhtml

persons from linkage and retention in HIV care, as well as, to understand both structural and individual facilitators that keep people engaged in care.

2) To understand how the Covid-19 pandemic has affected engagement and retention in HIV care for PLHIV.

Methods and Analysis

This study is grounded in principles of social justice, equity, and the understanding that PLHIV are experts whose voices should be at the center of the research design and process. Accurate findings are only achievable, robust, and meaningful if driven by people with lived experiences. The methodology of the study is interdisciplinary and intersectional, and utilizes participatory methods of engagement, drawing in part from Participatory Action Research (PAR) (See Figure 1).

Figure 1. Participatory Engagement Process
The participatory engagement process and how participatory action research will be utilized to achieve the outcomes related to objective 2 of this study [23–25].
*Research Objective: To explore the sex, gender-related, programmatic, structural barriers, and service gaps, as well as individual success factors associated with access to care and non-linkage to care in women, men, and non-binary persons living with HIV, before and during Covid-19, in both those who do and who do not inject drugs.

Patient and Public Involvement

This project is a collaborative multi-disciplinary study comprising peers, community-based researchers, clinicians, and academic researchers across Manitoba and Canada. To ensure this project remains grounded in the lived experience of PLHIV and PWID, we partnered with several community-based organizations, convened a diverse Research Advisory Committee (RAC), and created a peer research team consisting of people with lived experience.

Prior to the project commencement, the principal investigators held more than ten consultations with community members and peers, including PWID, PLHIV, and Indigenous

For peer review only - http://bmjopen.bmj.com/site/about/guidelines.xhtml

peers impacted by the “60s scoop” (i.e., children were taken from their homes and placed in residential schools or non-Indigenous homes; [26]). The community members compromised past and present people who use substances (including people who use methamphetamines) and come from various backgrounds. Many self-identified as Indigenous, and their personal stories illustrated the complex intersections of sex, gender, stigma, substance use, colonial and structural violence, and structural health access impediments. These consultations provided insight into the needs and experiences of the communities impacted by this research and first-hand knowledge of the circumstances that have led to increased substance use, particularly the injection of methamphetamine during the Covid-19 pandemic.

Several of the study co-investigators are the lead facilitators of HIV care and prevention in Manitoba, including The Manitoba HIV Program, which provides information, specialized care, and support to approximately 1500 PLHIV in Manitoba. Nine Circles Community Health Centre, The Manitoba Harm Reduction Network, Main Street Project, Meeting the Moment, and Sisters of Fire compromise the major partners guiding the research process.

Our research team has assembled a RAC (Supplementary material 1) that includes Indigenous Elders and Indigenous leaders, community members with lived experience, academic and scientific researchers with experience in the care of HIV, STBBIs, and sex and gender-based analysis, including experts from jurisdictions. The RAC guides the research team in research design and community-based engagement methods, data collection tools, promotion of the research study, the design of support for participants during and after the interview process, review of preliminary findings, and advice on optimal knowledge translation strategies. The RAC's continued involvement ensures that the project is culturally safe for the diverse

For peer review only - http://bmjopen.bmj.com/site/about/guidelines.xhtml

communities involved, the communities' best interests are considered, and the results and findings are shared appropriately with stakeholders.

To execute this study in collaboration with community experts, we have also convened a Peer Research Team of community members with lived experience (Supplementary material 2). The Peer Research Team's purpose is to actively design the research including the recruitment strategy and data collection tools, and to carry out research activities such as data collection, data analysis, development of findings, and knowledge translation. Our approach to peer engagement focuses on building reciprocal relationships and sharing skills with peer members for each step in the research process in a respectful and safe way. Peer Research Team meetings are held 2-3 times monthly for two and a half hours. Peers are paid for their time ($20 honorarium per hour) and provided with bus tickets and childcare support, if necessary, in addition to food, and activities (e.g., colour books).

A critical part of the research process will centre around reciprocity. Reciprocity is a critical component of Indigenous culture that should be applied as an overarching stance rather than a particular interaction (e.g., payment of honorarium) [27]. The commitment to PAR, collaboration with peers, and sharing skills and co-learning are components of a commitment to reciprocity. Another area where reciprocity will be incorporated is during the interview process (with PLHIV), connecting participants with care (if they consent), and offering participants other supports described in data collection, in addition to an honorarium and transportation support. Indigenous peoples make up 18% of the total population of the province of Manitoba (compared to 4.9% of the total population in Canada) [28]. While not all participants will identify as Indigenous, some will. The Indigenous community, peers, and Indigenous RAC members, as

For peer review only - http://bmjopen.bmj.com/site/about/guidelines.xhtml

well as experts in community-based research, have advised that an approach of reciprocity is therefore critical to ensuring our work is safe, meaningful, and collaborative.

Participants and Settings

Inclusion criteria for PLHIV include (Group 1):

1. Resident of Manitoba
2. Over the age of 18
3. Diagnosed with HIV between 2018-2021

Inclusion criteria for service providers (people who work with PLHIV) (Group 2):

1. Resident of Manitoba.
2. Provide medical, social, or other services to PLHIV

The target sample size for this prospective study will be 20-30 PLHIV and 20-30 service providers who work with PLHIV.

Recruitment Process

Purposive sampling will be used due to the distinct eligibility requirements of the participant groups.

Group 1 Recruitment Process

Service providers from the Manitoba HIV Program locations (e.g., Health Sciences Centre HIV Program, Nine Circles Community Health Centre, and 7th Street Health Access) will be asked to support the recruitment of PLHIV who are linked to care. Clinicians and health professionals in these locations provide HIV-related support services and have established and trusted relationships with PLHIV. Recruitment materials will be shared with clinicians and

For peer review only - http://bmjopen.bmj.com/site/about/guidelines.xhtml

service providers to discuss the study with potential participants. If participants are interested, they can contact the research team to discuss the study in more detail and confirm participation.

To support the recruitment of participants not linked to care, the research team will engage with community organizations supporting PLHIV who may use substances and may be experiencing unstable housing and mental health challenges. There has been a history of poor research relationships with PLHIV that have created experiences of mistreatment, exploitation, and disconnect between researchers and communities [29]. Therefore, it is critical to spend time in community locations where potential participants frequent to enable meetings, gain trust, and build rapport. Site observations at the Meeting the Moment program will create opportunities for connections since this program serves potential participants. Similarly, these observations will enable to engage in informal conversations with potential participants about the research, answer their questions, and then arrange for conducting interviews and questionnaires if people express interest. Purposive sample in group 1 will ensure that at least half of the participants self-identify as women, and that the experiences of Black, Indigenous, and People of Colour (BIPOC) are actively sought in our sample.

Group 2 Recruitment Process

In addition, a list of organizations that support and provide services to PLHIV will be compiled to recruit service providers. The project's advisory committee and community partners will suggest organizations and people who will be essential to consider for recruiting service providers. As with Group 1, a purposive sample will be employed to identify participants who provide different kinds of service and who identify as women, men, or non-binary. An aim of the recruitment strategy is to interview a representative sample of women, men, and non-binary persons who are providers. This selection process will enable the analysis of any gender-related

For peer review only - http://bmjopen.bmj.com/site/about/guidelines.xhtml

effects among service providers. Likewise, the perspectives of BIPOC service providers will be taken into consideration for sampling.

Data Collection

Potential participants who contact the research team by email or phone will be greeted by the research team. Research members will follow a phone or email script to discuss scheduling, accessibility, psychological, and emotional support that could be incorporated into the interview. This study's available psychological or emotional support will include mental health counselling, Indigenous Elder support, support persons, and peer support from people with lived experience. Participants will be offered information and access to health care related to HIV, other STBBIs, counselling, or to address other primary health concerns through referrals to project partners after completing data collection materials. All the field research teams have training in trauma-informed care, harm reduction, mental health first aid, and non-violent crisis intervention. This safety process ensures that participants have access to immediate support as some of the themes explored in the interviews and surveys may elicit emotional responses.

For PLHIV, data will be collected using a combination of face-to-face semi-structured interviews involving 26 open-ended questions (Supplementary Material 3). The interview guide used for this study has been revised and edited several times by members of the RAC, the Peer Research team, and key stakeholders (e.g., service providers). The interviews will be conducted by the research coordinator or the research associate, accompanied by one peer research team member. Peer research team members will be trained to conduct interviews and crisis intervention. However, many peer researchers already have experience in community-based research so they will be familiar with qualitative interview techniques, especially as it involves research engagement with marginalized groups. After the interviews, community advisors have

For peer review only - http://bmjopen.bmj.com/site/about/guidelines.xhtml

suggested to incorporate a scheduled break to offer various activities (e.g., bingo, meditation, stretching exercises) and food options (e.g., chocolate, bannock, berries) that create a welcoming and comfortable environment for participants.

Three surveys will be conducted after the break, which includes a Participant Demographic and Life Circumstances Survey (Supplementary Material 4), Childhood Trauma Questionnaire (Short-Form) [30], and the Empowr- Making Decisions Survey [31]. The Participant Demographics and Life Circumstances survey was developed by the research team in consultation with key stakeholders and it enquires about a participant's sex at birth, gender identity, sexual relationships, harm reduction practices, substance use, housing situation, and income. The second questionnaire, the Childhood Trauma Questionnaire- Short [30], is a self-reported inventory that employs Likert scale responses to assess different types of childhood mistreatment and understand the role of historical personal trauma on injection drug use and STBBI acquisition. Finally, the Empowr- Making Decisions Survey [31] (revised shortened version), employs a Likert scale to understand factors related to resilience and decision-making.

All interviews will take place in a private room at Nine Circles Community Health Centre or another agreed-upon private location to ensure participant confidentiality and researchers' safety. Participants will receive 50 CAD cash for their time at the end of the interview. Participants will be asked to sign an honorarium receipt form to maintain payment records. Also, participants will be provided with support to cover transportation costs fare to and from the interview, and childcare services if required.

For service providers, semi-structured interviews will involve 19 open-ended questions (Supplementary Material 5). The interview guide used for service providers has been revised and

For peer review only - http://bmjopen.bmj.com/site/about/guidelines.xhtml

edited several times by members of the RAC, and key stakeholders (e.g., service providers). All interviews will take place over Zoom or another online videoconferencing platform.

The research team members will explain the study and obtain consent from all participants before the interview. If written consent is not possible, the research team members will obtain verbal consent. The research team member will aim to email, text, or deliver a signed copy of the consent form to the participant following the interview for their records.

Each participant will be interviewed only once. Interviews will be recorded and are expected to last one to two and a half hours. In addition to recordings, research team members may take notes directly on the interview guide or in notebooks. All interviews will be conducted in English and fully transcribed for future analysis. After the completion of each interview, research assistants will participate in debriefing sessions with other research team members to increase study rigour.

Data Analysis

All interview data will be transcribed and entered in NVivo© software. Initial coding and verification will be done by the research associate and the research coordinator using a constant-comparison approach, and preliminary results will be shared with the peer research team members and RAC members for feedback and validation. Interview and survey results will be analyzed by sex at birth and gender.

Several strategies will be used to establish rigour and increase the trustworthiness of the data and data analysis process. The first strategy includes validating codes between three research team members to reduce reactivity, researcher bias, and respondent bias. Peer debriefing will be used for the peer research team members to share thoughts about the interviews (e.g., processes, questions, participant engagement) and ideas about codes and themes during data

For peer review only - http://bmjopen.bmj.com/site/about/guidelines.xhtml

analysis since it has a positive effect on reducing the threat of researcher bias [32]. Negative case analysis will also be used to reduce researcher bias throughout the data analysis process. Finally, an audit trail of decisions throughout the data collection and analysis process will be kept, increasing reproducibility. All raw data, memos, versions of the codebook, and generations of categories and themes will be kept and stored confidentially.

Additionally, quantitative data from the three questionnaires will be reported using descriptive statistics. These data will also be helpful toward developing a questionnaire for the next phase of a quantitative study with a larger sample size.

Ethics and Dissemination

This project received approval from the University of Manitoba Health Ethics Research Board (Ref: HS25572; H2022:218). As some participants will self-identify as First Nations, Métis or Indigenous, the project will follow Indigenous data sovereignty principles. Among these are the First Nation principles of OCAP®, Manitoba Métis principles of OCAS, the Principles of Ethical Métis Research, and CARE Principles for Indigenous Data Governance, under the leadership of the Advisory Committee. All data will be stored electronically by the Research Team. All folders and excel sheets will be password protected and stored on password-protected computers. In addition, all data from participants will be anonymized, and, if permission is granted, non-identifiable quotes will be used in future publications.

Findings will be disseminated through community-led knowledge translation strategies identified by participants, peers, community members, and community organizations; conference presentations, peer-reviewed journal articles, social media, workshops, and a dedicated webpage (https://nccid.ca/project-stream/hiv-STBBI/).

For peer review only

Discussion

Staggering inequities affect the lives of many disadvantaged groups across Canada [33]. In order to understand health inequities in the context of Manitoba it is crucial to contextualize the historical, political, social, and economic conditions that have influenced several groups. Since the 1980s there has been a growing trend in Manitoba where people with lower socioeconomic status experience worse health outcomes [34]. In Manitoba, injectable drug use has been established as the most common mode of HIV transmission [9]. Additionally, those who inject drugs are most at risk of receiving a diagnosis of other STBBIs and being affected by many social and biological factors, which is referred to as a syndemic. Preliminary data suggest that some groups are experiencing higher rates of HIV transmission than others. Similarly, the Covid-19 pandemic, accentuated pre-existing socioeconomic and structural factors, mental health concerns, violence, stigma, and discrimination, has increased sex and gender disparities and reduced the resources for STBBI and harm reduction services. Qualitative interviews with PLHIV and service providers can provide an in-depth understanding of people's barriers to accessing and maintaining HIV and STBBI treatment across Manitoba. The use of in-depth semi-structured interviews and questionnaires could also provide further insights into the sex- and gender-related, programmatic, and structural gaps of health associated with non-linkage to care in PLHIV who inject drugs compared to those who do not inject drugs before and during the Covid-19 pandemic.

This project is driven by participatory engagement processes that centre on the knowledge and expertise of community members, organizations, and people with lived experiences to improve the lives, health, and well-being of PLHIV in Manitoba and beyond. Our sample is restricted to people who live in Manitoba and those living in metropolitan areas (i.e.,

For peer review only - http://bmjopen.bmj.com/site/about/guidelines.xhtml

Winnipeg and Brandon, the two largest cities in Manitoba) and to individuals who can dedicate the time necessary to the interview process. However, our recruitment strategy to interview people linked to care and people not linked to care will ensure we have a more representative sample and gain a more comprehensive understanding of the factors that facilitate as well as inhibit access to care.

The findings will be used to propose changes to the delivery of testing, treatment, and support and to assist service providers and administrators in adapting STBBI- related health services to serve the population better. Our project will shed light on the growing segment of people, especially women living with HIV, who are missing from the 95-95-95 UNAIDS goal [19] during the Covid-19 pandemic and work with communities to improve health care and treatment. Contributing to the complex syndemics of HIV and other STBBIs, there are contextual factors in Manitoba, such as the ongoing impact of colonialism, experiences of violence, racial and gender discrimination, and underfunded health and social support systems that have failed many people in need of help and care. The results of this study will reveal a complex picture of the convergence of unjust structural and socioeconomic factors. Thus, these results will help to advocate for policy and systemic changes to improve the health and well-being of PLHIV in Manitoba.

Acknowledgments: We thank our partners Sisters of Fire, the Manitoba Harm Reduction Network, Main Street Project, Meeting the Moment, Nine Circles Community Health Centre, and Canadian AIDS Treatment Information Exchange (CATIE) for their ongoing guidance in this project. We are also thankful to members of the Research Advisory committee including Dr. Jacqueline Gahagan, Elder Margaret Lavallee, Veda Koncan, Jamil Mahmood, Margaret Bryan,

For peer review only - http://bmjopen.bmj.com/site/about/guidelines.xhtml

Jody Jollimore, and Peer Research Team members Ann Favel, Nadia Mancheese, Clara Dan, Joel Baliddawa, Lisa Patrick, Nikki Daniels, Marj Schenkels, and Rebecca Murdock for their invaluable insights.

Author Contributions: The study concept and design were conceived by ZV, MHB, and YK with input from LL, KM, KD, KT, LM, LI, KK, MP, AK, NP, JS, TM, JJ, and MB. EV and CS will schedule, collect consent, interview, and complete surveys with participants. EV, CS, and ZR prepared the first draft of the protocol manuscript. MHB, YK, LL, KM, KD, KT, LM, LI, KK, MP, AK, NP, JS, TM, JJ, MB, members of the Research Advisory Committee and the Peer Research Team provided critiques and suggestions for the manuscript.

Competing Interest Statement: There are no competing interests to report.

Funding: This work was supported by the Canadian Institute for Health Research (Grant number EG5-179453), The Manitoba Medical Service Foundation (grant number: 2021-13), The James Farley Memorial Fund. This research was also supported, in part, by the Canada Research Chairs Program for Z.V.R. Production of this manuscript has been made possible in part through a financial contribution of the Public Health Agency of Canada to the National Collaborating Centre for Infectious Diseases. The views expressed herein do not necessarily represent the views of the Agency.

Data Availability Statement: Data for this study is not available yet since this is a protocol paper. The study interview guides and questionnaires are available in the supplementary materials section.

For peer review only

References

1 Govender RD, Hashim MJ, Khan MA, *et al.* Global Epidemiology of HIV/AIDS: A Resurgence in North America and Europe. *J Epidemiol Glob Health* 2021;**11**:296–301. doi:10.2991/jegh.k.210621.001

2 Coovadia HM, Hadingham J. HIV/AIDS: global trends, global funds and delivery bottlenecks. *Glob Health* 2005;**1**:13. doi:10.1186/1744-8603-1-13

3 Biesma RG, Brugha R, Harmer A, *et al.* The effects of global health initiatives on country health systems: a review of the evidence from HIV/AIDS control. *Health Policy Plan* 2009;**24**:239–52. doi:10.1093/heapol/czp025

4 Hanefeld J. How have Global Health Initiatives impacted on health equity? *Promot Educ* 2008;**15**:19–23. doi:10.1177/1025382307088094

5 Shannon K, Crago A-L, Baral SD, *et al.* The global response and unmet actions for HIV and sex workers. *Lancet Lond Engl* 2018;**392**:698–710. doi:10.1016/S0140-6736(18)31439-9

6 Patel P, Borkowf CB, Brooks JT, *et al.* Estimating per-act HIV transmission risk: a systematic review. *AIDS Lond Engl* 2014;**28**:1509–19. doi:10.1097/QAD.0000000000000298

7 Haddad N, Robert A, Weeks A, *et al.* HIV in Canada—Surveillance Report, 2018. *Can Commun Dis Rep* 2019;**45**:304–12. doi:10.14745/ccdr.v45i12a01

8 Duncan KC, Reading C, Borwein AM, *et al.* HIV Incidence and Prevalence Among Aboriginal Peoples in Canada. *AIDS Behav* 2011;**15**:214–27. doi:10.1007/s10461-010-9792-y

9 Rueda Z, Sorokopud-Jones M, Sharp A, *et al.* Syndemics in people newly diagnosed with HIV in Manitoba, Canada. Importance of coinfections and comorbidities. Montreal, Canada: 2022.

10 Argento E, Strathdee SA, Goldenberg S, *et al.* Violence, trauma and living with HIV: Longitudinal predictors of initiating crystal methamphetamine injection among sex workers. *Drug Alcohol Depend* 2017;**175**:198–204. doi:10.1016/j.drugalcdep.2017.02.014

11 Chenneville T, Gabbidon K, Hanson P, *et al.* The Impact of COVID-19 on HIV Treatment and Research: A Call to Action. *Int J Environ Res Public Health* 2020;**17**:E4548. doi:10.3390/ijerph17124548

12 Public Health Agency of Canada. Survey of the impact of COVID-19 on access to STBBI-related health services including harm reduction services among people who use drugs or alcohol in Canada. 2021.https://health-infobase.canada.ca/datalab/covid-19-stbbi-services.html (accessed 22 Jul 2022).

13 Galloway G, Buscemi R, Coyle J, *et al.* A Randomized, Placebo-Controlled Trial of Sustained-Release Dextroamphetamine for Treatment of Methamphetamine Addiction. *Clin Pharmacol Ther* 2011;**89**:276–82. doi:10.1038/clpt.2010.307

For peer review only - http://bmjopen.bmj.com/site/about/guidelines.xhtml

14 Singer M, Bulled N, Ostrach B, *et al.* Syndemics and the biosocial conception of health. *Lancet Lond Engl* 2017;**389**:941–50. doi:10.1016/S0140-6736(17)30003-X

15 Center for Disease Control and Prevention (CDC). Understanding the HIV Care Continuum. 2017.https://www.cdc.gov/hiv/pdf/library/factsheets/cdc-hiv-care-continuum.pdf (accessed 9 Aug 2022).

16 Tso LS, Best J, Beanland R, *et al.* Facilitators and barriers in HIV linkage to care interventions: a qualitative evidence review. *AIDS Lond Engl* 2016;**30**:1639–53. doi:10.1097/QAD.0000000000001101

17 Dombrowski J. Linkage to Care. Natl. HIV Curric. 2020.https://www.hiv.uw.edu/go/screening-diagnosis/linkage-care/core-concept/all (accessed 10 Aug 2022).

18 Hall BJ, Sou K-L, Beanland R, *et al.* Barriers and Facilitators to Interventions Improving Retention in HIV Care: A Qualitative Evidence Meta-Synthesis. *AIDS Behav* 2017;**21**:1755–67. doi:10.1007/s10461-016-1537-0

19 UNAIDS. Understanding Fast-Track Accelerating Action To End the Aids Epidemic by 2030. 2015.https://www.unaids.org/sites/default/files/media_asset/201506_JC2743_Understanding_FastTrack_en.pdf (accessed 16 Aug 2022).

20 Scully EP. Sex Differences in HIV Infection. *Curr HIV/AIDS Rep* 2018;**15**:136–46. doi:10.1007/s11904-018-0383-2

21 Everett BG. Sexual Orientation Disparities in Sexually Transmitted Infections: Examining the Intersection Between Sexual Identity and Sexual Behavior. *Arch Sex Behav* 2013;**42**:225–36. doi:10.1007/s10508-012-9902-1

22 Ostrach B, Singer M. At special risk: Biopolitical vulnerability and HIV/STI syndemics among women. *Health Sociol Rev* 2012;**21**:258–71. doi:10.5172/hesr.2012.21.3.258

23 Reason P, Bradbury H, editors. *The Sage handbook of action research: participative inquiry and practice*. 2nd ed. London ; Thousand Oaks, Calif: : SAGE Publications 2008.

24 Allen W. Participatory action research provides for multiple benefits - Learning for Sustainability. Learn. Sustain. 2016.https://learningforsustainability.net/post/par/ (accessed 25 Jul 2022).

25 Organizing engagement. Participatory Action Research and Evaluation. Organ. Engagem. 2019.https://organizingengagement.org/models/participatory-action-research-and-evaluation/ (accessed 10 Aug 2022).

26 National Collaborating Centre for Aboriginal Health. Indigenous Children and the Child Welfare System in Canada. 2017.https://www.nccih.ca/docs/health/FS-ChildWelfareCanada-EN.pdf

For peer review only - http://bmjopen.bmj.com/site/about/guidelines.xhtml

27 McGregor HE, Marker M. Reciprocity in Indigenous Educational Research: Beyond Compensation, Towards Decolonizing. *Anthropol Educ Q* 2018;**49**:318–28. doi:10.1111/aeq.12249

28 Statistics Canada. Focus on Geography Series, 2016 Census. 2017.https://www12.statcan.gc.ca/census-recensement/2016/as-sa/fogs-spg/Facts-PR-Eng.cfm?TOPIC=9&LANG=Eng&GK=PR&GC=46 (accessed 27 Jul 2022).

29 Logie C. *Working with Excluded Populations in HIV: Hard to Reach or Out of Sight?* Cham: : Springer International Publishing 2021. doi:10.1007/978-3-030-77048-8

30 Bernstein D, Stein J, Newcomb M, *et al.* Development and validation of a brief screening version of the Childhood Trauma Questionnaire. 2003;**27**:169–90. doi://doi.org/10.1016/S0145-2134(02)00541-0

31 Rogers ES, Ralph RO, Salzer MS. Validating the empowerment scale with a multisite sample of consumers of mental health services. *Psychiatr Serv Wash DC* 2010;**61**:933–6. doi:10.1176/ps.2010.61.9.933

32 Heilman J, Figg C, Wenrick M, *et al.* Implications and Benefits of a Long-Term Peer Debriefing Experience on Teacher Researchers. *Brock Educ J Educ Res Pract* 2010;**19**:20–35. doi:10.26522/brocked.v19i1.118

33 Public Health Agency of Canada. Key health inequalities in Canada: a national portrait. 2018.http://epe.lac-bac.gc.ca/100/201/301/weekly_acquisitions_list-ef/2019/19-06/publications.gc.ca/collections/collection_2019/aspc-phac/HP35-109-2018-1-eng.pdf (accessed 17 Aug 2022).

34 Martens P, Brownell M, Au W, *et al.* Health Inequities in Manitoba: Is the Socioeconomic Gap Widening or Narrowing Over Time? 2010.http://mchp-appserv.cpe.umanitoba.ca/reference/MCHP-Health_Ineq_summary_final_WEB.pdf (accessed 17 Aug 2022).

For peer review only

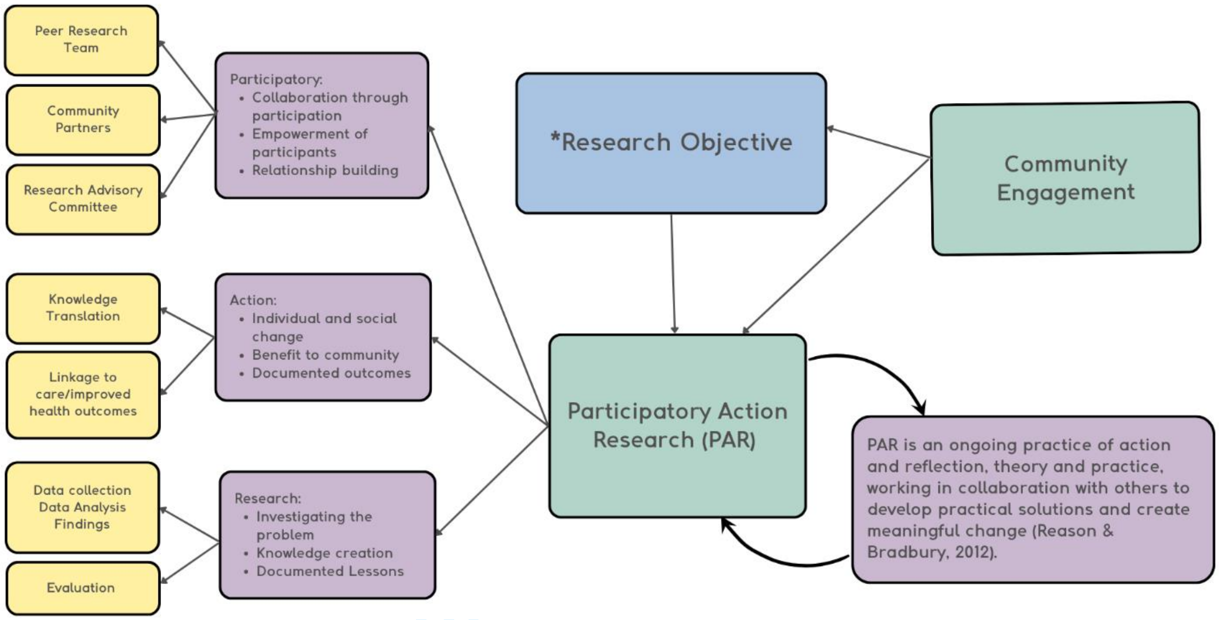

Figure 1. Participatory Engagement Process

The participatory engagement process and how participatory action research will be utilized to achieve the outcomes related to objective 2 of this study [23–25].

*Research Objective: To explore the sex, gender-related, programmatic, structural barriers, and service gaps, as well as individual success factors associated with access to care and non-linkage to care in women, men, and non-binary persons living with HIV, before and during Covid-19, in both those who do and who do not inject drugs.

Terms of Reference for the Research Advisory Committee

Background:

In Manitoba, injection drug use is the most common mode of HIV transmission since 2018. Those who inject drugs are more likely to test positive for HIV, syphilis and Hepatitis C, often at the same time. This is called a "syndemic" (many epidemics at the same time). For the last three years, syphilis infections have become rampant in Manitoba. If not found and treated, syphilis can cause devastating infection, including transmission to newborns. In addition to HIV and syphilis there are 11 other infections transmitted by sexual activity or blood products (called STBBI).
For some people, a convergence of socio-economic factors, mental health concerns, violence and exploitation, is driving a syndemic of new HIV and other STBBI cases, with disproportionate representation of women who inject drugs. Methamphetamine use is growing in Manitoba and is known to affect access to care and treatment. These determinants of health and disparities are exacerbated by COVID-19 related public health measures, as resources for testing and contact tracing for Covid-19 have resulted in decreased capacity for service providers to respond to STBBI and distribute harm reduction services. It is unknown how both biological sex differences and gender intersect with living conditions including experiences of violence and injection drug use.

This study seeks to know:

1. How many people in Manitoba (by sex, gender and other identifiers) living with HIV have other sexually transmitted and blood-borne infections (STBBI), before and during Covid-19?
2. Of those living with HIV, whom are most at risk of acquiring other STBBI, and why?
3. What are the barriers and gaps that put people at risk for infection and prevent them from getting appropriate care and treatment before and during Covid-19?
4. What are the resilience factors and promising practices that are associated with successful engagement of people living with HIV and who inject methamphetamine that endured during the Covid-19 pandemic?

Gaining an in-depth understanding of the experiences of women, men and non-binary persons living with HIV who use and don't use drugs can help us identify unique gaps as well as resilience factors that can inform and improve the Manitoba HIV Program, and result in tailored testing and appropriate clinical and program responses.

Principal Investigators:

Dr. Zulma Rueda. University of Manitoba. Email: zulma.rueda@umanitoba.ca
Dr. Yoav Keynan University of Manitoba. Email: yoav.keynan@umanitoba.ca
Ms. Margaret Haworth-Brockman, University of Manitoba. Email: Margaret.Haworth-Brockman@umanitoba.ca

Advisory Committee's Role and Purpose

The Advisory Committee will advise the research team on the following aspects of the project:

- ✓ Research approach and community-based methods
- ✓ Guidance the implementation of research.
- ✓ Promotion of the research study.

For peer review only

- ✓ Advice on community engagement plans.
- ✓ Interpretation of findings.
- ✓ Help to identify and strategize any potential solutions to community issues that may arise.
- ✓ At all times, strictly maintain confidentiality for all aspects of the research project as outlined in the University of Manitoba *Access and Privacy policy* and *Access and Privacy Procedure.*

- ✓ **Input and advice from the Advisory Committee will help to ensure that**:
  - ✓ The project/study is culturally appropriate for the diverse communities involved.
  - ✓ The research team conducts the project in the communities' best interests.
  - ✓ The research results and knowledge will be shared appropriately with communities and stakeholders.
  - ✓ Action plans associated with the research receive community input and are appropriate to community needs and concerns.

All Advisory Committee members will be eligible for compensation for their time involved. We expect there to be about 5-7 meetings over 12 months, with one or two meetings being in the early stages of the project development.

Responsibilities from the research team:

1. Ensure the ethical conduct of the research study.
2. Provide accurate information related to the project.
3. Investigate and answer the suggestions of the research advisory committee.

Disclosures:

- ✓ All member will declare any real or perceived conflict of interest at the outset of any meeting for management in accordance with the University of Manitoba *Conflicts of Interest Policy and Conflict of Interest Procedures.*
- ✓ All research project conducted within the University of Manitoba is governed by the *Responsible Conduct of Research Policy, Responsible Conduct of Research – Code of Research Ethics Policy, Responsible Conduct of Research – Investigation Procedure.*
- ✓ This research is funded by the Canadian Institute of Health Research; therefore, we follow and adhere to the *Tri-Agency Framework: Responsible Conduct of Research, including the framework and policies of Chapter 9: Research Involving the First Nations, Inuit and Métis Peoples of Canada.*

Term

This research is initially funded for one year.

Memberships

The Committee will be composed by the following members, in addition to the 3 principal investigators. Our meetings will be opened in a Good Way with the help of an Elder.

The number and composition of members comprises of:

1. Five or six community members – we are actively recruiting advisors now. We are inviting members who include:

For peer review only

a. Person who lives with HIV
b. Person who has current or former experience with injection drug use
c. Women or female who have experienced sexual assault, sexual violence, experiencing violence or sexual exploitation
d. Person who is experiencing homelessness or who are unsheltered
e. Person who identify themselves as 2SLGBTQQIA+
f. Person who belong to Indigenous population or racialized communities

2. Elder Margaret Lavallee, Elder in Residence, Ongomiizwin
3. Julianne Sanguins. Adjunct Scientist at the Manitoba Centre for Health Policy, and Assistant Professor at the Department of Community Health Sciences, University of Manitoba.
4. Tara Faye Myran, Metis Community member.
5. Ann Favel, Person with lived experience. Member of Sisters of Fire
6. Jaqueline Flett, Person with lived experience. Member of Sisters of Fire
7. Marj Schenkels, Person with lived experience. Member of Sisters of Fire
8. Nikki Daniels. Community member with lived experience.
9. Jody Jollimore. Executive Director at CATIE
10. Margaret Bryan. Manitoba Harm Reduction Network
11. Jacqueline Gahagan. Associate Vice-President, Research, Mount Saint Vincent University. Halifax, Nova Scotia.
12. Adrienne FA Meyers, Associate Director of the JC Wilt Infectious Diseases Research Centre
13. Neora Pick, Clinical Professor in the Infectious Diseases division of the Department of Medicine at the University of British Columbia, and Medical Director at the Oak Tree Clinic.
14. Veda Koncan, Projects Manager, Manitoba Harm Reduction Network
15. Chantal Daniels, Elder Support, Indigenous Institute of Health & Healing, Ongomiizwin

Meeting frequency

Meetings will be called by the Project Manager.
The committee will meet for five or seven 2-hour meetings either in person in Winnipeg or via teleconference/web-based technology for members residing outside Winnipeg.

Sitting fees

Research team will provide $20 CAD/per hour honorariums for people who are eligible for honorarium
Research team will cover costs for transportation for people residing in Winnipeg

Contact:

Dr. Zulma Rueda. University of Manitoba. Email: zulma.rueda@umanitoba.ca
Dr. Yoav Keynan University of Manitoba. Email: yoav.keynan@umanitoba.ca
Ms. Margaret Haworth-Brockman, University of Manitoba. Email: Margaret.Haworth-Brockman@umanitoba.ca

For peer review only

Cheryl Sobie. Research project coordinator. University of Manitoba. Email: Cheryl.Sobie@umanitoba.ca

For peer review only

Peer Terms of Reference

Purpose:

We want to engage people with lived experience as peers in our project and work collaboratively to ensure all aspects of the research are shaped and guided by the perspectives of peers with lived experience. Peer engagement will inform the entire research process, from conducting research interviews, to skill sharing, workshops, and developing educational materials to share the results of our research. We recognize the unique knowledge and expertise of community members with lived experience, and we seek your guidance and input for our research study.

Background Information:

We are peers, clinicians and researchers who want to understand infections transmitted by sex or blood in people living with HIV, who may use drugs, as well as why people living with HIV are not getting the care they need. We are interested in knowing which factors, barriers and gaps prevent people from accessing care. We will also look for resilience factors that keep people linked to care.

We are interested in better understanding the impact of the COVID-19 pandemic and how it may have affected people newly diagnosed with HIV (between 2018-2021) in accessing health care and other resources. We want to know things such as:

- What was it like navigating the health care system during the pandemic?
- What are ways we can make things better for people to be linked to health care?
- What things are working well for people that keep them going to get health support and resources before and during the COVID-19 pandemic?

Project Time Period:

July 2022 – March 2023

Peer Roles available:

1. Peer Research Advisory Committee Member
2. Peer Support Worker
3. Peer Group Team Member

Role (1) Peer Research Advisory Committee Member (see Research Advisory Committee Terms of Reference Document). Key items to note:

How does this work?
You will join our committee which includes peers like you and some researchers. Together you will advise the research team about the best ways to:

- involve other people in the research study (using posters, or word of mouth, or in certain places);

For peer review only

- pay or compensate them for their time (for example, taxi coupons, bus passes, cash, or pre-paid VISAs);
- questions that are appropriate in interviews;
- how to understand what we find from the results;
- how to share the results of the study with the community;
- other education that the community or health care providers need to know. There may be other things you provide advice on.

Meeting frequency:

The Research Advisory Committee will meet for five or seven 2-hour meetings either in person in Winnipeg or via teleconference/web-based technology for members residing outside Winnipeg.

Compensation:

The research team will provide $20 CAD/per hour honorarium for peer research advisory committee members. Research team will cover costs for transportation (bus fare, taxi) for people residing in Winnipeg to attend the meeting.

<u>Role (2) Peer Support Worker:</u>

How does this work?

- Provide peer support for participants (people living with HIV) during research interview
- You will sit in the interview with participant and research team member to provide support as needed
- When we are scheduling interviews, we will ask participants if they would like a peer support worker at their interview
- Must sign confidentiality agreement and maintain confidentiality at all times
- We plan to interview 20-30 people; we are unsure at this point how many will require support
- The interview will take place in community settings such as Nine Circles, HSC HIV Clinic or another community location as determined by the participant and research team member

Time Required:

- 2-3 hours per interview session
- We anticipate interviews will take place between the end of August - October 2022

<u>Role (3) Peer Research Team Member</u>

How does this work?

For peer review only

- The purpose of the peer research team is to bring together community members with lived experience to work together to conduct research (interviews, data analysis, review findings), develop ideas for how we will share the results of our research, review comments from the advisory committee, and provide guidance and direction on the research for recruitment, community engagement, etc.
- The peer team is separate from the advisory board; however, a peer group member may also sit on the advisory board.
- The work of the peer team will include items such as:
  - Develop research skills and conduct research interviews, support data analysis and review findings of research
  - Discuss issues raised by the advisory board
  - Develop ideas and collaborate to prepare educational material for the community based on the results and findings of the research
  - To learn from each other either by skill sharing (i.e., doing computer courses provided by university, learning new skills related to grant writing/proposal development, learning technologies such as Zoom, etc.); we want to hear from YOU what you want to learn about
  - To advocate and share with the public what it is that is needed in Manitoba to break the stigma of HIV and drug use
- The peer team will consist of ten community members with lived experience
- Members will be selected by members that want to participate. If too many people apply, we will narrow out the group by selecting someone from each of the criteria in line with the research

Meetings:

<u>Meeting Frequency & Location</u>

The peer team will meet every two to three weeks for two hours. We will meet in person wherever possible at the University of Manitoba Bannatyne campus, or outside at a park. Should things change depending on the COVID-19 situation and safety guidelines we may have to meet online using Zoom or Teams.

<u>Comfort & Safety</u>

We will create a safe space for all group members to participate in, which includes psychological safety and cultural safety. Members can participate to the maximum comfortable amount.

We reject any form of discrimination or racism. We are committed for a safe and inclusive environment for everyone to participate. We do not judge each other and will respect each other at all times. We are committed to making your participation as accessible as possible. If there is anything you need from us to support you, please let us know.

<u>Activities</u>

For peer review only

Meetings will focus on relationship building, skill sharing, and project discussions. Once the data collection is completed, we will then turn our attention to creating educational materials to share the results of the research.

In addition to the research and skill sharing activities we want to incorporate fun activities or cultural activities such as beading, drum circles, bringing an Elder, playing games, sharing food together, music, etc.

Supplies

At each meeting:

- We will provide light lunches or snacks and drinks
- Cover any training and education costs
- Provide supplies for making educational materials
- Bring materials required for any activities as discussed

Compensation:

The peer group role will be compensated with an honorarium in the amount of $20/hour cash and bus tickets for transportation will be provided. Childcare will be compensated as needed $10 per hour. No money other than honorarium's will be received by peer members.

- ✓ **Confidentiality**- all team members will respect the confidentiality of other team members and any items discussed within group meetings.
- ✓ All members will declare any real or perceived conflict of interest at the outset of any meeting for management in accordance with the University of Manitoba *Conflicts of Interest Policy and Conflict of Interest Procedures.*
- ✓ All research project conducted within the University of Manitoba is governed by the *Responsible Conduct of Research Policy, Responsible Conduct of Research – Code of Research Ethics Policy, Responsible Conduct of Research – Investigation Procedure.*
- ✓ This research is funded by the Canadian Institute of Health Research; therefore, we follow and adhere to the *Tri-Agency Framework: Responsible Conduct of Research, including the framework and policies of Chapter 9: Research Involving the First Nations, Inuit, and Métis Peoples of Canada.*

For peer review only

Covid-19 Pandemic Concealing a Syndemic of Concern: Sex, Gender, Methamphetamine and Sexually Transmitted and Blood Borne Infections in People Living with HIV in Manitoba

Participant Interview Question Guide

To be completed by facilitator	*Facilitator Name*		
Participant ID:			
Participating Organization:			
Location:			
Interview date:			
Interview time:	Start	End	Duration
Confirmation of consent signed:			

Thank you for agreeing to speak with me today. My name is [INSERT] and my pronouns are INSERT] and this is my colleague [introduce themselves]. My role with this project is as a [Research Assistant, Research Associate, Research Coordinator]. Is there anything you need to do to prepare yourself before we get started? We anticipate the interview and short surveys will take between 1-2 hours to complete. We can take a break during the question portion of this interview if you need to, and we will take a short break after the interview before we begin the surveys. Please let me know at any time if you need to step away for a few minutes.

[NOTE: This next section only applies to people who will not have booked an interview appointment and are a walk-up participant]

Before I explain the purpose and what we will do today I want to check in with you about support we can offer you today and I have three questions I would like to ask you:

1. ***Would you be interested in having a peer support to sit with you for your interview? The purpose of a peer support is only to provide emotional support and they will maintain confidentiality of anything we discuss today.***
2. ***Would you be interested in having cultural support? There is an Elder available who you can speak with at any time if you need a break during the interview, at the break between the interview and surveys or at the end of the interview.***
3. ***Are you interested in speaking with a psychological support person at the break or after your interview today? They are available anytime if you need to stop the interview to debrief or check in with them.***

For peer review only - http://bmjopen.bmj.com/site/about/guidelines.xhtml

Covid-19 Pandemic Concealing a Syndemic of Concern: Sex, Gender, Methamphetamine and Sexually Transmitted and Blood Borne Infections in People Living with HIV in Manitoba

Before we start the formal interview questions, I would like to remind you that we are here to speak about your experiences in finding and receiving HIV and STBBI-related health care and services. Our aim is to improve HIV and other services for everyone. We are interviewing people with many stories and, perhaps, some questions may not relate to your life experiences. We appreciate your responses since many of these questions are personal. There are some questions about substance use that may or may not apply to you. The reason we are asking these questions is because our research has shown that in Manitoba, the main way people get HIV is through injection drug use but that is by no means the only way people get HIV. We understand that some people may feel shame or stigma about having HIV or using drugs, which is why we have tried to create a safe and comfortable space to listen to your experiences. It is completely up to you if you want to answer the questions or not.

You have been asked to participate in this interview as you:

1) *Are 18 years or older*
2) *Have received a diagnosis for HIV between 2018-2021*
3) *Are a resident of Manitoba*

Do all of these statements apply to you? ***Y / N***

[If no, relay that they are not eligible to participate in the study. Thank them for their time, and end interview].

For the purpose of our discussion, STBBI services may include testing, care and counselling for HIV, Hepatitis C, syphilis, other sexually transmitted infections like Chlamydia, or gonorrhoea.

We take your information seriously which is why we also want to remind you that everything you share today will be kept confidential. This means that we will not disclose any information you share with us about drug use, engaging in sex work or anything else that could be perceived as "criminal" with authorities. The only reason we would ever have to break confidentiality is if there is a direct risk/danger to your health and safety or that of someone who is under 18. We would like to record this interview to make sure the information is accurate. This recording will be kept in a secure location and destroyed after this study is over. This recording will not be shared.

For your participation today, we will give you a $50 honorarium in the form of cash and bus fare or money to cover a taxi ride to attend the interview. Additionally, we have several items that you can choose from as a gift and a form of appreciation for the valuable insight you have shared with us today.

I would like to start with some questions about how things generally are for you, but before we start are there any questions you want to ask?

For peer review only - http://bmjopen.bmj.com/site/about/guidelines.xhtml

Covid-19 Pandemic Concealing a Syndemic of Concern: Sex, Gender, Methamphetamine and Sexually Transmitted and Blood Borne Infections in People Living with HIV in Manitoba

1. I'd like to get to know you a little better, could you please tell me about yourself?
   a. PROBES
      i. How are things going for you right now?
      ii. How are you feeling coming in today?

2. What does a typical week (day) look like for you?
   a. PROBES
      i. Daily routine
      ii. Work/volunteer
      iii. Medical programs/services
      iv. Time with friends/family
      v. Time with caring for friends/family

3. How are things going with your health right now?
   a. PROBES- ask about:
      i. Physical health
      ii. Mental health
      iii. Emotional health
      iv. Spiritual health
      v. Financial health

Now I would like for us to talk about the HIV care and other health services that you receive.

4. What was your experience like when you were first diagnosed with HIV?
   a. PROBES
      i. Did you have support or post counselling offered?
      ii. Were you given enough information about health care options? (i.e. explained about your rights, how to access to care, follow up, linkage to doctor, treatment options?)
      iii. Did you learn about how HIV is transmitted? And how to prevent transmission?
      iv. Did you receive information about importance of treatment?

5. What health services do you currently receive for HIV health care?
   a. PROBES,
      i. Attend clinic appointments, see a NP, take medication, regular blood testing, etc. (Linked to Care)
      ii. Alternative medicine such as traditional or cultural medicine
      iii. Anything else?
   b. **If No, not linked to care move to question 7**

Linked to care = attended clinical services more than two times, and/or do you engage in any HIV services regularly, such as taking ART medication, counselling, doctor visits, regular blood tests, etc.

For peer review only - http://bmjopen.bmj.com/site/about/guidelines.xhtml

Covid-19 Pandemic Concealing a Syndemic of Concern: Sex, Gender, Methamphetamine and Sexually Transmitted and Blood Borne Infections in People Living with HIV in Manitoba

6. **What do you like about the HIV health care services you receive?**
   a. PROBES
      i. Relationship with doctor/ nurse/ social worker
      ii. Accessibility of location to receive services
      iii. Welcoming environment at service provider location
      iv. Any other reason?

7. **What barriers do you encounter when accessing HIV care or barriers that prevent you from accessing care (if not currently linked to care)?**
   a. PROBES
      I. Stigma, discrimination from service providers
         i. Drug use, unhoused, gender identity
         ii. If yes, please tell me more about that. Where does this discrimination occur?
      II. Transportation, accessibility challenges
      III. Lack of childcare to get to attend appointments or children are not able to accompany to appointment
      IV. Income, lack of money to pay for medication
      V. Other challenges are more important to deal with
      VI. Lack of access to appointments, lengthy wait times to receive care, etc.
      VII. Mental health challenges
      VIII. Any other barriers?

8. **Could you please walk me through a time when you needed HIV health care services and did not receive them?**
   a. PROBES
      i. Ask participant to share a specific experience related to a barrier
      ii. If you were diagnosed before the pandemic (2018-2019), did you experience similar or different challenges before or during the COVID-19 pandemic (2020-current)?

9. **Has the COVID-19 pandemic impacted the HIV health services you use? If yes, in what ways?**
   a. PROBES
      i. Service-related changes such as availability of appointments, shortage of staff at clinics, services not accessible, staff changes, etc.
      ii. Increased fear/risk due to COVID-19 in health care settings
      iii. Transportation issues
      iv. Money, financial challenges
      v. Use of substances
      vi. Mental health challenges/and or lack of support for mental health

(Note: this question will be adapted depending on when the participant first received a HIV diagnosis)

For peer review only - http://bmjopen.bmj.com/site/about/guidelines.xhtml

Covid-19 Pandemic Concealing a Syndemic of Concern: Sex, Gender, Methamphetamine and Sexually Transmitted and Blood Borne Infections in People Living with HIV in Manitoba

10. How safe do you feel in or around health care settings?

a. PROBES
   i. In the neighborhood around the clinic/hospital
   ii. Around security personnel such as police, hospital security guards
   iii. Fear of Child & Family Services, removal of children, or fear of other government agencies/actors
   iv. Lack of privacy to discuss health status (i.e., ER visit)
   v. Not safe due to perceived gender identity, race/ethnicity
   vi. Getting to your appointment- location of clinic, transportation

11. What are the things that help you to get the health care you need?

a. PROBES
   i. Positive relationship with doctor/health professionals
   ii. Welcoming and supportive environment at health centres, health centres specific to my needs (i.e. gender specific, LGBTQ+)
   iii. Availability/hours
   iv. Location within the city
   v. Support (personal or peer) to attend health appointments

12. What changes do you think would make it easier for you (and other people living with HIV) to access care?

a. PROBES
   i. Transportation support, financial support
   ii. Peer support (i.e., someone to accompany you to appointments)
   iii. Better accessibility (e.g., different sites for treatments, availability of appointments, options for care)
   iv. Access to counselling and/or mental health support
   v. Addressing stigma
   vi. Different medication with less side effects
   vii. Access to material support (housing, food, etc.)
   viii. Bundling more services in one appointment- seeing multiple professional, etc. primary care physician, gynaecologist, mental health professional,
   ix. Addressing multiple health concerns at each appointment- either related to HIV or other health issue

I have a few more questions before we end the interview, they are related to substances you may use. These questions may not apply to you, and we want to assure you that there is no judgment based on your use of substances. These questions will help us find ways to better support people who are living with HIV who also may use substances. I also want to remind you that you do not have to answer any questions you do not feel comfortable with, and that all of your answers are confidential.

13. Do you have any experience with using substance?
IF YES, ask the following questions. If no, move to QUESTION 18

For peer review only - http://bmjopen.bmj.com/site/about/guidelines.xhtml

Covid-19 Pandemic Concealing a Syndemic of Concern: Sex, Gender, Methamphetamine and Sexually Transmitted and Blood Borne Infections in People Living with HIV in Manitoba

14. How old were you when you started using substances?
- a. PROBE:
  - i. What substance(s) did you start with?

15. How would you describe your substance use when you started?
- a. PROBES:
  - i. Heavy use (one or more times per day)
  - ii. Moderate use (few times per week)
  - iii. Light use (once a week or a few times a month)
  - iv. Very light (less than once a month)

16. What type of substances do you currently use?
- a. PROBES
  - i. If crystal meth- do you inject or smoke or both?
  - ii. If opioids- inject or smoke, or pill?
- b. **If YES to injection ask question 17, if NO skip question 18**

17. How would you describe your current substance use?
- a. Probes
  - i. Several times per day (one or more)
  - ii. Few times a week
  - iii. Once a week or a few times a month
  - iv. Less than once a month

18. How do you access the substances you use?
- a. PROBE
  - i. Do you get it yourself or does someone else get them for you?)

This next series of questions we are interested in better understanding the practices you use to inject drugs.

19. If you use substances that you inject, how often do you:
- a. **Use a new needle, spoon, sterile water or filter?**
  - i. All of the time, some of the time, rarely or never?
  - ii. If only some of the time or never- what are the barriers to accessing safe injection supplies?

- b. **Inject with a needle or syringe used by somebody else?**
  - i. All of the time, some of the time, rarely, or never?

- c. **Clean and disinfect the injection site?**
  - i. All of the time, some of the time, rarely, or never?

- d. **Prepare using a spoon, water, or filter used by somebody else?**

For peer review only - http://bmjopen.bmj.com/site/about/guidelines.xhtml

i. All of the time, some of the time, rarely, or never?

e. **Have a safe place to use?**
   i. All of the time, some of the time, rarely, or never?
   ii. If yes, what makes this place safe?
   iii. If no, what makes this place unsafe?

20. Do you know where to find harm reduction supplies?
   a. Probe
      i. If yes, where do you get them from?
      ii. What supplies do you get?
      iii. How often do you get harm reduction supplies?
      iv. Know where to find harm reduction supplies

21. Have you experienced any violence related to your substance use?
   a. PROBE
      i. If yes, and you feel comfortable, can you please tell me what happened?

22. What would make using substances safer for you?
   a. PROBE
      i. Better access to harm reduction supplies
      ii. Safe injection site
      iii. Decriminalization of drug use
      iv. Safe person to use drugs with

23. Does using substances affect your ability to access HIV care & services?
   a. PROBES
      i. If yes, in what ways?

24. How did the COVID-19 pandemic impact your substance use?
   a. PROBE
      i. What changes in your pattern of use- availability, safety of substance, harm reduction supplies?
      ii. Changes to type of substance used or ways of using- did you use more of one substance or start injecting instead of smoking?

25. What do you think is the best way [how do you want] to learn about prevention and treatment of STBBI's and harm reduction practices?

26. Is there anything else you want to tell us today?

That is the end of the interview portion of our time together today.

Thank you very much for speaking with me. I really appreciate you taking the time today to do this. I know some of these questions were very personal and might have brought up some

For peer review only - http://bmjopen.bmj.com/site/about/guidelines.xhtml

emotions for you. If you need support after our chat, I can provide you a list of resources ***[give participant mental health support resource document].*** *[Insert name of counsellor or Elder] is available to speak with you now if you would like that support. You can also speak to [Participating Organization] and they can direct you to any other supports you can access.*

Now that we have completed the interview portion of the interview, we will take a short break ***[discuss with the participant what they need to do- offer Elder support, food, break to walk around, smudge ceremony, stretch/move their body, etc.].*** *When we come back together, I will ask you to please fill out three short surveys. This should take another 30-45 minutes to complete. After that if there are things, you want to add to this discussion, we will make sure you have enough time to do that.*

For peer review only

For peer review only - http://bmjopen.bmj.com/site/about/guidelines.xhtml

Service Provider Interview Question Guide

To be completed by facilitator	*Facilitator Name*		
Participant ID:			
Participating Organization:			
Location:			
Interview date:			
Interview time:	Start	End	Duration
Confirmation of consent signed:			

Thank you for agreeing to speak with me today. My name is [INSERT] and my pronouns are INSERT]. My role with this project is as a [Research Assistant, Research Associate].

Before we start the formal interview questions, I would like to remind you that we are here to speak about your experiences as a service provider [clinic program providers and/or social service employee] to provide additional perspectives on institutions, structures and policies related to HIV Care in Manitoba. The interview should take approximately 45 to 60 minutes.

Everything you share with me will be kept confidential. I also want to remind you that that our conversation will be recorded so your words are captured as accurately as possible, but the recording will be kept securely and destroyed once the study is over.

You have been asked to participate in this interview as you:

1) *Are a clinic service provider or social service employee who works with and/or provides services to people living with HIV or other STBBI's in Manitoba [and/or people who inject drugs]*

Does this statement apply to you?
Yes
No

For peer review only - http://bmjopen.bmj.com/site/about/guidelines.xhtml

[If no, relay that they are not eligible to participate in the study. Thank them for their time, and end interview].

For the purpose of our discussion, STBBI Services may include:

- *HIV testing / care*
- *Hepatitis C testing / care*
- *Syphilis testing / care*
- *Other sexually transmitted infection (STI) testing /care*
- *Obtaining safe sex products (condoms, dental dams, pre/post exposure prophylaxis*
- *Information about STBBI testing/care*
- *Counselling related to STBBIs*
- *Community services or supports related to STBBIs*

Do you have any questions for me before we begin?

1. **Could you please describe your role?**
    a. PROBES
        i. Type of organization (Clinic, CHC, Community Organization)
        ii. Position Title
        iii. Main job duties
        iv. Schedule

2. **What services does your organization provide?**
    a. PROBES
        i. Women's health, mental health, addictions?

3. **Please tell me about what services are working well for your clients/patients living with HIV?**
    a. PROBES
        i. Specific supports or programs
        ii. Accessibility
        iii. Campaigns
        iv. New services to deal with COVID-19

4. **What barriers to accessing or remaining in care have you identified for people living with HIV?**
    a. PROBES
        i. Attitudes to some clients
        ii. Limited hours or sites
        iii. Shortages of supplies or staff
        iv. Person's gender identity
        v. Stigma towards infectious diseases, substance use, race, etc.

For peer review only - http://bmjopen.bmj.com/site/about/guidelines.xhtml

5. **What barriers have you identified for PLHIV who are actively using substances that may prevent them from accessing care?**
   a. PROBES
      i. Attitudes of staff
      ii. Administrative regulations
      iii. Stigma associated with substance use, race, gender
      iv. Behaviour challenges related to substance use

6. **What major changes have you noticed in your clients/patients due to the COVID-19 pandemic?**
   a. PROBES
      i. Increased drug use
      ii. Increased experiences of violence
      iii. Mental health challenges
      iv. Housing instability
      v. Income instability
      vi. Incarceration
      vii. Increase in sex work
      viii. Suicidal ideation
      ix. Anything else?

7. **What are some examples of success factors that you have seen clients employ during the COVID-19 pandemic to ensure they continue to get the care they need?**

8. **How has the COVID-19 pandemic affected the services in your organization?**
   a. PROBES
      i. Changes of hours
      ii. Not enough staff to meet client needs
      iii. Virtual platform challenges
      iv. Increased needs of clients related to drug use
      v. Strain on health care system & referral services
      vi. Impact on staff to deliver services
         1. Burnout
         2. Getting sick themselves
         3. Not enough training for new staff

9. **How has the COVID-19 pandemic affected you personally as a service provider?**
   a. PROBES
      I. Mental health challenges
      II. Burnout
      III. Secondary trauma
      IV. Increased work load
      V. Covid-19 reinfections

10. **How do you support people whose needs fall outside of the services you offer?**
   a. PROBE

For peer review only - http://bmjopen.bmj.com/site/about/guidelines.xhtml

i. Collaborate with other health providers or community agencies to connect people to these services
ii. Relationships with other providers in the community

11. What strategies do you currently use to support people living with HIV who use substances?

a. PROBES
 i. What additional training, resources, education do you need?

12. What is your understanding of harm reduction and how do you apply this in your work?

a. PROBES
 i. Harm reduction- knowledge, attitudes
 ii. What else do you want to learn about harm reduction?

13. What strategies do you use to support your clients with prevention of STBBI's?

a. PROBES:
 i. PrEP, STBBI testing, counselling/mental health support
 ii. Education/sharing knowledge about STBBI's- how they are transmitted, how are the treated?

14. What do you personally do or what does your organization do to create a safe space/environment for clients?

a. PROBES
 i. Environment- accessibility, meeting clients where they are at, safety
 ii. Training- anti-racism, anti-oppression
 iii. Policies- sexual harassment, violence, covid safety, harm reduction
 iv. Practices- cultural safety, trauma informed care

15. Can you tell me what you think about the policies in Manitoba related to HIV?

a. PROBES
 i. Specific policies working against ability to get, receive or remain engaged in care
 ii. Policies currently working well to support PLHIV
 iii. Policies you would create to support you in your work as a service provider

16. What are your suggestions for policy change or program enhancements- that would both support PLHIV and service providers delivering care?

a. PROBES
 i. Additional options for care, additional services
 ii. Referrals to other services
 iii. Bundling services in one appointment or location (wrap around care)

17. What additional resources are needed to better support people living with HIV in Manitoba?

a. PROBES

For peer review only - http://bmjopen.bmj.com/site/about/guidelines.xhtml

For peer review only

i. Prevention- i.e. PREP
ii. Additional options for care
iii. Knowledge about STBBI's- how they are transmitted, how are the treated?

18. **What do you think is the best way [how do you want] to learn about prevention and treatment of STBBI's and harm reduction practices?**

19. We are asking everyone involved in this study to share their gender. If you are comfortable, can you please tell us how you identify?

That is the end of our interview today. *Before we go, I wanted to know if you wanted to share anything else with me? Or if you think we should have asked you about something else?*

Thank you very much for speaking with me. We really appreciate your time and efforts and the valuable information you have shared with us today.

For peer review only

For peer review only - http://bmjopen.bmj.com/site/about/guidelines.xhtml

Participant Survey: Demographics & Life Circumstances

The following short questionnaire asks questions about you, such as your age, identity, where you live, your experiences with substance use, your sexual practices, and other life circumstances you may have encountered. This information is very helpful as it provides a better understanding of your experiences as a person living with HIV. We understand that these questions are very personal and intimate and we appreciate your responses. **There is no judgement about anything you share with us**. Every person has different life experiences and situations that affect their health and well-being. Some of these questions may not apply to you, **please only answer what is relevant to your life**.

Please **circle the answer(s) that applies to you or write your answer in the space provided. If there are questions you do not feel comfortable answering, please skip them.** Please ask your interviewer if you would like them to better explain any of the questions.

Thank you for completing this survey.

PART A: Demographics

1. How old are you? ______________________

2. What is your gender identity?
   a. Woman
   b. Man
   c. Non-binary
   d. Two-Spirit
   e. Other ______________________
   f. Prefer not to say

3. What sex were you assigned at birth? (as shown on your original birth certificate)
   a. Female
   b. Male
   c. Intersex
   d. Prefer not to say

For peer review only - http://bmjopen.bmj.com/site/about/guidelines.xhtml

For peer review only

4. What is your cultural background? (**Please circle any answer that applies to you)**
   a. First Nations or Indigenous
   b. African
   c. European
   d. East Asian
   e. South Asian
   f. South East Asian
   g. Hispanic or Latinx
   h. Middle Eastern
   i. Other (please specify) ________________________
   j. Prefer not to answer

5. What race would you identify yourself as? (**Please circle any answer that applies to you)**
   a. Asian
   b. Black
   c. European/White
   d. Hispanic
   e. Indigenous
   f. Pacific Islander
   g. Don't know
   h. Prefer not to answer

6. If you Identify as Indigenous, do you identify as:
   a. First Nations
   b. Métis
   c. Inuk
   d. Other ______________________

7. What is your sexual orientation?
   a. Lesbian
   b. Gay
   c. Bisexual
   d. Asexual
   e. Heterosexual/Straight
   f. Pansexual
   g. Queer
   h. Other ______________________
   i. Prefer not to say

For peer review only - http://bmjopen.bmj.com/site/about/guidelines.xhtml

PART B. Income and Housing

Now we will ask you some questions related to your income and housing. These answers will allow us to better understand the resources that people living with HIV may need to support their health and well-being.

8. What is your main source or source(s) of income? ______________________

9. Do you currently have enough income to cover all your life expenses?
   a. Yes
   b. No

10. **Please circle** any of the following items that you currently **do not** have enough income to cover:
    a. Housing
    b. Transportation
    c. Food
    d. Clothing
    e. Childcare
    f. Pet care
    g. Health care
    h. Other ___________________________

11. What is your current living situation? **(Please circle any answer that applies to you)**
    a. Living alone (house/apartment)
    b. Living with partner (married, common-law or relationship)
    c. Living with children
    d. Living with roommates
    e. Living with extended family members
    f. Living in a homeless shelter, halfway house, substance use treatment centre
    g. Currently experiencing housing instability
       i. If yes, for how long have you been experiencing unstable housing? ______________________________

12. **If experiencing housing instability,** we mean any of the following. **(Please circle any answer that applies to you)**
    a. Insecure housing (For example, month to month, threats by landlord to leave, unsure how you will pay rent next month)
    b. Couch surfing
    c. Sleeping rough
    d. Staying at a shelter or other short-term housing program

For peer review only - http://bmjopen.bmj.com/site/about/guidelines.xhtml

13. Did you experience housing instability prior to being diagnosed with HIV? Or after you were diagnosed with HIV? **(Please circle any answer that applies to you)**
    a. Yes, before I was diagnosed
    b. Yes, after I was diagnosed
    c. Yes, before and after I was diagnosed
    d. Only after I was diagnosed
    e. Only before I was diagnosed
    f. No experiences of housing instability
    g. Other ______________________

PART C: Criminal Justice System

The next few questions we want to ask you are related to your experiences with the criminal justice system. We are asking these questions as recent HIV infections have shown a number of infections in people who were incarcerated. These questions will help us to also understand how policing and surveillance may be affecting your life and health. We do not judge any of your answers and we are not affiliated in any way with police or the justice system.

14. Have you ever been incarcerated?
    a. Yes
        i. If yes, what year (or years) were you incarcerated. From _____________ to _____________
        _____________ to _____________
        _____________ to _____________
    b. No **(Skip to Part D)**

15. Did you receive an HIV positive diagnosis while incarcerated?
    a. Yes
        i. If Yes, who told you about your diagnosis **(Please circle)**
            - Doctor
            - Administrations
            - Prison Guard
    b. No

16. **If Yes,** did you experience discrimination while incarcerated because of your HIV diagnosis
    a. Yes
    b. No

For peer review only - http://bmjopen.bmj.com/site/about/guidelines.xhtml

17. Have your experiences with the criminal justice system affected your ability to access HIV care and other health care services?

a. Yes
b. No

18. While you were incarcerated did you have access to any of the following: **(Please circle any answer that applies to you)**

a. Primary health care services (i.e. regular physicals, doctor appointments)
b. HIV related care (i.e. medication, regular blood testing, doctor appointments)
c. STBBI testing
d. Harm reduction supplies (syringes, condoms, masks, hand sanitizer)
e. Mental health services and counselling

19. Are you currently under correctional supervision (e.g., bail conditions, probation, or parole)?

a. If **yes,** how many months or years will this supervision last? ___________

PART D: Sexual Practices

Now we would like to ask you some questions about your sexual practices. Please answer what you are comfortable with. We are asking these questions as people can acquire HIV through sexual activity but that is not the only way. The reason we are asking these questions is to help us better understand the sexual practices of people living with HIV which can be useful for health care providers when informing safe sex education.

20. Are you currently sexually active?

a. Yes
b. No

21. In the past year have you had sex with: **(Please circle any answer that applies to you)**

a. Women
b. Men and women
c. Men
d. Non-binary persons
e. Two-spirit person
f. Other_____________________
g. Prefer not to answer

22. In the past 12 months how many sexual partners have you had?

a. 0
b. 1-5
c. 5-10

For peer review only - http://bmjopen.bmj.com/site/about/guidelines.xhtml

d. 10-20
e. 20-50
f. 50+
g. Prefer not to answer

23. When engaging in sexual activity, do you use any of the following safe sex protections? **(Please circle any answer that applies to you)**
a. Condom
b. Dental dam
c. Pre/post exposure prophylaxis

24. If yes, how often do you use these protections? **(Please circle any answer that applies to you)**
a. All of the time
b. Some of the time
c. Never
d. I want to use protections but they are too difficult to access (e.g. PrEP)

25. How easy was it to get safe-sex protections before and during the COVID-19 pandemic? **(Please circle any answer that applies to you)**
a. Easier before COVID-19 pandemic
b. No difference prior to and during COVID-19 pandemic
c. Harder to get during COVID-19 pandemic
d. Other_________________________________

26. Do you use other forms of contraception such as: **(Please circle any answer that applies to you)**
a. Short-acting **hormonal** methods (e.g. Oral birth control, patch, ring, injection)
   i. Please list which type ___________________________________
b. Long Acting Reversable Contraceptive (e.g.IUD)
   i. Please list which type _____________________________
c. Barrier Methods (e.g. condom, sponge, diaphragm, cervical cap)
   i. Please list which type _____________________________
d. Natural rhythm methods
e. Sterilization (i.e. tubal ligation or vasectomy)
   i. Please list which type _____________________________
f. Other ___________________________________

This next question only applies to people who use substances. We are asking these questions because the use of substances (crystal meth, crack cocaine) can sometimes cause an increase in desire for sexual activity.

For peer review only - http://bmjopen.bmj.com/site/about/guidelines.xhtml

27. Does substance use impact your sexual practices in any of the following ways: **(Please circle any answer that applies to you)**

a. I am more likely to engage in sex with multiple partners when using substances
b. I am less likely to engage in sex with multiple partners when using substances
c. I am less likely to use protection when engaging in sexual activity when using substances
d. I am more likely to use protection when engaging in sexual activity when using substances
e. Prefer not to say
f. Other ____

28. How often do you get blood tests for sexually transmitted infections and blood born infections (such as HIV, Hep C)? **(Please circle any answer that applies to you)**

a. Before engaging in sexual activity with a new partner
b. Once a month
c. Every few months
d. Once a year
e. When my doctor/nurse practitioner recommends
f. Never
g. Other __________________________________

PART E: Substance Use

We would now like to ask you about your substance use. These questions may or may not apply to you, and we want to assure you that there is no judgment based on your use of substances. We are asking these questions to understand how to better support people who are living with HIV who also may use substances

29. How old were you when you started using substances? ____________________

What substance did you start with? ______________________________

30. How would you describe your substance use when you started? **(Please circle any answer that applies to you)**

a. Heavy use (one or more times per day)
b. Moderate use (few times per week)
c. Light use (once a week or a few times a month)
d. Very light (less than once a month)

For peer review only - http://bmjopen.bmj.com/site/about/guidelines.xhtml

31. How would you describe your current substance use? **(Please circle any answer that applies to you)**

a. Heavy use (one or more times per day)
b. Moderate use (few times per week)
c. Light use (once a week or a few times a month)
d. Very light (less than once a month)

32. Please fill in this chart with the substances you use and how often you use them [1]

**Type**	**How often per day**	**How often per week**	**How often per month**
Alcohol			
Opiates/Opioids (Heroin, fentanyl, oxy, down)			
Crack			
Cocaine			
Crystal meth			
Cannabis			
Tobacco			
Other Substance:			

For peer review only

33. If you use substances that you inject, how often do you: **(Please circle any answer that applies to you)**

a. Use a clean needle
   i. Some of the time
   ii. All of the time
   iii. Not very often
   iv. Never

b. Inject with a needle or syringe used by somebody else
   i. Some of the time
   ii. All of the time
   iii. Not very often
   iv. Never

c. Prepare using a spoon, water, or filter used by somebody else

For peer review only - http://bmjopen.bmj.com/site/about/guidelines.xhtml

i. Some of the time
ii. All of the time
iii. Not very often
iv. Never

d. Have a safe place to use
    i. Some of the time
    ii. All of the time
    iii. Not very often
    iv. Never

e. Know where to find harm reduction supplies
    i. Some of the time
    ii. All of the time
    iii. Not very often
    iv. Never

PART F: Experiences with Violence

For the last portion of this survey, we want to better understand your experiences with violence. This section may or may not apply to you. The reason we are asking these questions is because during the COVID-19 pandemic there was a reported increase in experiences of violence, in particular intimate partner violence and domestic violence. There is also a higher rate of women who have been diagnosed with HIV in Manitoba over the past few years – and women, transgender, non-binary people, as well as those who identify as Indigenous and 2SLGBTQQIA+ are more likely to experience all forms of violence.

34. Throughout your life, have you ever experienced any of the following types of violence? **(Please circle any answer that applies to you)**

a. Physical violence
b. Sexual violence
c. Stalking, harassment
d. Emotional abuse
e. Intimate Partner Violence (i.e violence perpetrated by a partner in a romantic or dating relationship)
f. Domestic Violence (i.e violence among people in a domestic situation- can include any type of family, or roommate)
g. Cyberstalking/cyberbullying
h. Child abuse
i. Psychological / mental violence
j. Other_______________________________
k. Prefer not to say

For peer review only - http://bmjopen.bmj.com/site/about/guidelines.xhtml

35. If yes, what type or types of violence have you experienced in the past 3 years?

____

36. Are you currently experiencing any type of violence?

a. Yes
   i. If yes, which type(s)
   ____
b. No

37. Have you received any support or health care services to support you with healing from experiences of violence (trauma counselling, psychological support, traditional healing, etc.)?

a. Yes. If yes, what forms of support did you access
   ____
b. No

38. If you are currently experiencing Intimate Partner Violence (i.e violence perpetrated by a partner in a romantic or dating relationship) does this impact your ability to access health care in any of the following ways: **(Please circle any answer that applies to you)**

a. I am not able to attend HIV clinic appointments or HIV related care because my partner is controlling (of my time and schedule, money for health care costs, etc.
b. I am afraid of my partner finding out about when I am going to doctor appointments or taking medication
c. My health care is not impacted, and I am able to attend all my appointments
d. Other __________________________

That brings us to the end of the survey. Thank you again for taking the time to fill out this survey. We really appreciate your time and willingness to share your experiences with us. If you have any questions or would like to debrief with the interviewer, support person (Elder, counsellor, etc.) that can happen now. We have also created a resource sheet to access support or counselling if you would like a copy. We will now take a break before the next portion of our time together today.

For peer review only
For peer review only - http://bmjopen.bmj.com/site/about/guidelines.xhtml

BMJ Open

Social and structural barriers and facilitators to HIV health care and harm reduction services for people experiencing syndemics in Manitoba: Study protocol

Journal:	*BMJ Open*
Manuscript ID	bmjopen-2022-067813.R1
Article Type:	Protocol
Date Submitted by the Author:	11-Apr-2023
Complete List of Authors:	Rueda, Zulma; University of Manitoba Faculty of Health Sciences, Department of Medical Microbiology and Infectious Diseases<br>Haworth-Brockman, Margaret; National Collaborating Centre for Infectious Diseases; University of Manitoba, Department of Community Health Sciences<br>Sobie, Cheryl; University of Manitoba Faculty of Health Sciences, Department of Medical Microbiology and Infectious Diseases<br>Villacis, Enrique; University of Manitoba Faculty of Health Sciences, Department of Medical Microbiology and Infectious Diseases<br>Larcombe, Linda; University of Manitoba Faculty of Health Sciences, Department of Internal Medicine<br>Maier, Katharina; The University of Winnipeg, Criminal Justice<br>Deering, Kathleen; The University of British Columbia, Department of Medicine; Centre for Gender & Sexual Health Equity<br>Sanguins, Julianne; Manitoba Metis Federation; University of Manitoba, Department of Community Health Sciences<br>Templeton, Kimberly; Manitoba HIV Program; Nine Circles Community Health Centre<br>MacKenzie, Lauren; Manitoba HIV Program; University of Manitoba Faculty of Health Sciences, Department of Internal Medicine<br>Ireland, Laurie; Nine Circles Community Health Centre; Manitoba HIV Program<br>Kasper, Ken; University of Manitoba, Internal Medicine; Manitoba HIV Program<br>Payne, Michael; Nine Circles Community Health Centre<br>Bullard, Jared; University of Manitoba, Medical Microbiology and Infectious Diseases; Cadham Provincial Laboratory<br>Krusi, Andrea; The University of British Columbia, Department of Medicine; Centre for Gender & Sexual Health Equity<br>Pick, Neora; Oak Tree Clinic; The University of British Columbia, Division of Infectious Disease<br>Myran, Tara; University of Winnipeg, Indigenous Development<br>Meyers, Adrienne; Indigenous Services Canada, Laboratory Integration, Office of Population and Public Health<br>Keynan, Yoav; University of Manitoba Faculty of Health Sciences, Internal Medicine; National Collaborating Centre for Infectious Diseases
**Primary Subject Heading**:	HIV/AIDS

Secondary Subject Heading:	Epidemiology, Qualitative research
Keywords:	COVID-19, HIV & AIDS < INFECTIOUS DISEASES, QUALITATIVE RESEARCH, Sexual and Gender Minorities, Substance misuse < PSYCHIATRY, Health Services Accessibility

SCHOLARONE™
Manuscripts

BMJ Open Submission

Revised Manuscript – Clean version

Title: Social and structural barriers and facilitators to HIV health care and harm reduction services for people experiencing syndemics in Manitoba: Study protocol

Authors:

Zulma Vanessa Rueda M.D., Ph.D.[1*ǂ], Margaret Haworth-Brockman M.Sc.[2,3ǂ], Cheryl Sobie M.A.[1], Enrique Villacis M.A.[1], Linda Larcombe Ph.D.[4], Katharina Maier Ph.D.[5], Kathleen Deering Ph.D.[6,7], Julianne Sanguins Ph.D.[3], Kimberly Templeton M.Sc.[9,10], Lauren MacKenzie M.D., MPH. [4,9], Laurie Ireland M.D.[9,10], Ken Kasper M.D.[4,9], Michael Payne B.A.[9,10], Jared Bullard M.D.[1,11], Andrea Krusi Ph.D.[6,7], Neora Pick M.D.[12,13], Tara Myran B.A.[14], Adrienne Meyers M.Sc. Ph.D.[15], Yoav Keynan M.D., P.h.D.[1,2,3,4,9ǂ]

[1] Department of Medical Microbiology and Infectious Diseases, University of Manitoba Rady Faculty of Health Sciences, Winnipeg, MB, R3E 0J9, Canada

[2] National Collaborating Centre for Infectious Diseases, Rady Faculty of Health Sciences, University of Manitoba, Winnipeg, MB, R3E 0T5, Canada

[3] Department of Community Health Sciences, University of Manitoba, Rady Faculty of Health Sciences, Winnipeg, MB, R3E 0J9, Canada

[4] Department of Internal Medicine, University of Manitoba Rady Faculty of Health Sciences, Winnipeg, MB, R3E 0J9, Canada

[5]Criminal Justice, The University of Winnipeg, Winnipeg, Canada

For peer review only - http://bmjopen.bmj.com/site/about/guidelines.xhtml

[6]Division of Social Medicine, Department of Medicine, Faculty of Medicine, The University of British Columbia, Vancouver, Canada

[7]Centre for Gender & Sexual Health Equity, Vancouver, Canada

[9]The Manitoba HIV Program, Winnipeg, Canada

[10] Nine Circles Community Health Centre, Winnipeg, Canada

[11] Cadham Provincial Laboratory, Winnipeg, Canada

[12] Oak Tree Clinic, BC Women's Hospital, Vancouver, Canada

[13] Division of Infectious Disease, University of British Columbia, Vancouver, Canada

[14] Indigenous Development, University of Winnipeg, Winnipeg, Canada

[15] Laboratory Integration, Office of Population and Public Health, Indigenous Service Canada, Winnipeg, Manitoba

[Ł] Principal investigators

***Correspondence**: Zulma Vanessa Rueda. Email: zulma.rueda@umanitoba.ca

For peer review only

Abstract

Introduction:

In Manitoba, Canada, there has been an increase in the number of people newly diagnosed with HIV and those not returning for regular HIV care. The Covid-19 pandemic resulted in increased sex and gender disparities in disease risk and mortalities, decreased harm reduction services and reduced access to health care. These health crises intersect with increased drug use and drug poisoning deaths, houselessness, and other structural and social factors most acutely among historically underserved groups. We aim to explore the social and structural barriers and facilitators to HIV care and harm reduction services experienced by people living with HIV (PLHIV) in Manitoba.

Methods and analysis:

Our study draws upon participatory action research (PAR) design. Guiding the methodological design are the lived experiences of PLHIV. In-depth semi-structured face-to-face interviews and quantitative questionnaires will be conducted with two groups: (1) Persons ≥18 years old newly diagnosed with HIV between 2018 and 2022, and (2) service providers who work with PLHIV. Data collection will include sex, gender, sociodemographic information, income and housing, experiences with the criminal justice system, sexual practices, substance use practices and harm reduction access, experiences with violence and support, HIV care journey (since diagnosis until present), childhood trauma and a decision-making questionnaire. Data will be analyzed intersectionally, employing grounded theory for thematic analysis, sex- and gender-based analysis, and social determinants of health and syndemic framework to understand the experiences of PLHIV in Manitoba.

For peer review only - http://bmjopen.bmj.com/site/about/guidelines.xhtml

Ethics and dissemination:

We received approval from the University of Manitoba Health Ethics Research Board (HS25572;H2022:218), First Nations Health and Social Secretariat of Manitoba, Nine Circles Community Health Centre, Shared Health Manitoba (SH2022:194), and 7th Street Health Access Centre. Findings will be disseminated using community-focused knowledge translation strategies identified by participants, peers, community members, and organizations, and reported in conferences, peer-reviewed journals, and a website (www.alltogether4ideas.org).

Keywords: HIV care, HIV barriers and facilitators, syndemic, participatory action research, Covid-19, gender.

Strengths and Limitations

- A diverse research advisory and peer research team, and comprehensive data collection tools ensure we accurately capture people's insights into HIV care and harm reduction services.
- The intersectional lens would provide a better understanding of the current social and structural health inequities experienced by people living with HIV.
- The sample may not represent all circumstances since data collection will occur in two metropolitan areas and with individuals who can dedicate time to the study.

For peer review only - http://bmjopen.bmj.com/site/about/guidelines.xhtml

INTRODUCTION

Approximately 36.9 million people live with the human immunodeficiency virus (HIV) [1,2]. Despite global and local initiatives to address HIV [2–5], recent findings suggest a resurgence of diagnoses among North and South American and European countries [1]. In Canada, HIV diagnoses have steadily increased among women and people who inject drugs (PWID) [6,7]. In Manitoba, Canada, there is an overrepresentation of females, PWID, Indigenous peoples, and people experiencing houselessness among people newly diagnosed with HIV [8]. Heterosexual sex and injection drug use have become the most common mode of HIV transmission since 2018, and methamphetamine is the main injected drug [8]. In Manitoba, there is also an emerging pattern of people living with HIV (PLHIV) not returning for regular care and treatment. Manitoba also has not reached any of the UNAIDS' and Canada's 95-95-95 goal (i.e., 95% of all PLHIV know their status, 95% of those diagnosed receive antiretroviral treatment, and 95% of those on treatment achieve viral suppression) [9,10].

The care continuum for PLHIV encompasses several interconnected components (e.g., diagnosis, linkage and engagement to care) [11,12]. Linkage to care has been associated with positive health outcomes and reduced HIV mortality [12,13]. In a systematic qualitative review, Tso and colleagues [12] found that interventions focused on increasing community participation and literacy, creating community outreach mobile teams, integrating HIV-specific services in primary care, and providing substance use services improved linkage to care [12]. On the other hand, lack of information, anxiety, fear, and stigma after an HIV diagnosis were barriers to linkage to care [12]. Moreover, Hall and researchers [14] found that the leading reasons for people continuing HIV care were the use of community health workers and lay health workers, incorporating technology with health, support from friends and family, access to intensive case

For peer review only - http://bmjopen.bmj.com/site/about/guidelines.xhtml

management, and positive interpersonal relationships with service providers [14]. Personal and community stigma and discrimination, fear of HIV disclosure, and service provider shortages were among the leading barriers to PLHIV staying in care [14].

Previous findings have also highlighted how HIV experiences are shaped by biological sex and gender identities [15–18]. Ostrach and Singer [18] emphasize the importance of biological, social, and political factors that place women at an increased risk of acquiring HIV. The authors suggest integrating sex- and gender-based analyses in HIV research to accurately capture the impact of intersectional identities on PLHIV [18]. Argento et al. found that most women (i.e., cis and transwomen) living with HIV, who participate in sex work, and may or may not use methamphetamines had experienced physical or sexual abuse during childhood and adolescence, and those who initiated methamphetamines use were more likely to have experienced childhood abuse [15]. Their study emphasizes the need to consider childhood trauma in understanding health and social circumstances later in life [15]. The Canadian prairie provinces (Alberta, Saskatchewan, and Manitoba) report higher violence against women than the rest of Canada [19]. Findings in these provinces highlight the harm violence has on women's lives [20] and emphasize the importance of considering women's contexts and intersecting identities (e.g., cultural background, gender, disability) when developing person-centred interventions [20–23].

The Covid-19 pandemic has dramatically affected the health of many individuals, especially among people already burdened by social and structural health inequities [24]. The pandemic also exacerbated mental health symptoms in many groups [25]. Data from Manitoba shows that older adolescents and young adults self-reported increased stress/anxiety and depression, alcohol consumption and substance use, and conflict with family members and

For peer review only - http://bmjopen.bmj.com/site/about/guidelines.xhtml

intimate partner[26], with those self-identifying as women experiencing higher mental health burden [27,28], financial hardship, and interpersonal conflicts [26]. For HIV care, the reduction or complete closure of HIV treatment centres and services in some jurisdictions placed the cascade of care for PLHIV at risk of breaking down [24]. Among PWID, Covid-19 restrictions limited safe spaces for substance use, disrupted drug supplies, and restricted the availability of medical, community, and traditional resources [29].

Harm reduction encompasses strategies to reduce the health, social, and economic factors that harm PWID and their networks [30]. The Public Health Agency of Canada conducted an online survey among people self-identifying as PWID in the past six months to understand how sexually transmitted and blood borne infections (STBBIs) and harm reduction services changed during Covid-19 [31]. The survey reported 52% of respondents increased their personal use of methamphetamine, and more than half increased their use of alcohol and other drugs [31]. As well, 50% of PLHIV had challenges getting to a provider or clinic since the onset of Covid-19 [31]. More than half of the respondents had difficulty getting STBBI-related services, needle and syringe distribution programs, and naloxone training [31]. While these results highlight concerning trends, data were not reported by sex and gender, hindering an intersectional analysis. Also, the study's online modality may not have reached PWID who may also be coping with unstable housing, poverty, and other structural factors, likely underestimating the disruption of services. Locally, the province of Manitoba reported record fatalities from toxic drug overdoses [32], and PWID described harm reduction services limitations due to Covid-19 [29]. However, more research is needed to understand specific service limitations and how these limitations interact with other health crises and social inequities.

For peer review only - http://bmjopen.bmj.com/site/about/guidelines.xhtml

Taken together, these trends suggest that an intersectional lens is needed to understand how social, structural and programmatic factors affect HIV care and harm reduction services among PLHIV, including in the context of Covid-19 disruptions. This paper reports a study protocol that aims to address the question: What are the social and structural factors that women, men and non-binary people living with HIV in Manitoba are experiencing that prevent or enable HIV care and harm reduction services, before and during Covid-19 pandemic?

STUDY OBJECTIVES AND OUTCOMES

This study explores the social and structural factors that affect the access, linkage and engagement in HIV health care and harm reduction services among women, men, and non-binary persons living with HIV (linked and non-linked to care), before and during Covid-19.
It is important to note that this study is one component of a more extensive mixed-methods project aimed at understanding the epidemiology of HIV and syndemics experienced by PLHIV in Manitoba.

Outcomes

1) To gain an in-depth understanding of the social and structural barriers and facilitators faced by women, men and non-binary persons living with HIV, affecting access, linkage and engagement in HIV health care and access harm reduction services, before and during Covid-19 pandemic.
2) To understand how the Covid-19 pandemic has affected access, linkage and retention in HIV health care and harm reduction services for women, men and non-binary persons living with HIV.

For peer review only - http://bmjopen.bmj.com/site/about/guidelines.xhtml

METHODS AND ANALYSIS

Conceptual framework

The convergence of simultaneous epidemics (HIV and STBBIs, substance use, trauma and violence, mental health issues), called syndemics [33,34], and social and structural health inequities that disproportionately affect oppressed and underserved populations in Manitoba requires understanding health as a social construct, rather than a biological process. The World Health Organization Conceptual Framework for Action on the Social Determinants of Health (CSDH) [35] describes how social, economic, and political values and policies create a socioeconomic position, for people in societies, influencing opportunities for income, education, depending on race/ethnicity, gender, and other factors [35]. These structural determinants of health inequities interact with intermediary determinants of health such as material circumstances, and psychosocial, biological (including sex) and behavioural factors [35]. The framework helps to push understanding of inequities beyond a focus on individual health and behaviors, framing health as a social construct dependent on the interplay of numerous interacting systems and circumstances.

Similarly, the syndemic framework proposed by Singer [33] focused on understanding the interconnectedness of mutually reinforcing biomedical crises within their socioeconomic contexts. Imperative to syndemic theory is the recognition that disparate social conditions not only enable health crises but maintain and reinforce them [33]. This framework is particularly needed in the context of communicable diseases and Indigenous peoples' health, as it emphasizes the political, social, historical, and economic factors that have negatively affected their health outcomes [36]. These two frameworks guide the theoretical approach for this study (Figure 1).

For peer review only - http://bmjopen.bmj.com/site/about/guidelines.xhtml

Figure 1. World Health Organization Conceptual Framework for Action on the Social Determinants of Health [35] adapted to the main focus of our study.

A comprehensive framework that includes structural and social factors, is needed in Manitoba to understand the experiences of PLHIV that prevent and enable access, linkage and retention in HIV care and harm reduction services. Our methodology is intersectional by considering how sex and gender, cultural background, socioeconomic factors, (including housing), trauma and violence, substance use, empowerment and decision making, and other factors intersect [37] to affect individuals' experiences of HIV care and harm reduction services before and during Covid-19 pandemic.

Patient and Public Involvement

This study is grounded in principles of social justice, equity, and the understanding that PLHIV are experts whose voices should be central to the research design and process. Accurate findings are achievable, robust, and meaningful only if driven by people with lived experiences. Therefore, this study utilizes community engagement grounded in participatory action research (PAR; Figure 2).

Figure 2. Participatory Engagement Process

Legend: The participatory engagement process and how Participatory Action Research (PAR) will be utilized to achieve the outcomes related to this study [38–40]. *Research Objective: This

For peer review only - http://bmjopen.bmj.com/site/about/guidelines.xhtml

study explores the social, structural and programmatic factors that affect the access, linkage and retention to HIV care and harm reduction services among women, men, and non-binary persons living with HIV (linked and non-linked to care), before and during Covid-19.

This project is a collaborative multi-disciplinary study connecting peers, community-based researchers, clinicians, and academic researchers across Canada. To ensure this project remains grounded in the lived experience of PLHIV and PWID, we partnered with several community-based organizations, convened a diverse Research Advisory Committee (RAC), and created a Peer Research Team (PRT) of people with lived experience.

Before the project commencement, the principal investigators held more than ten consultations with community members and peers, including PWID, PLHIV, and Indigenous peers impacted by the "60s scoop" (i.e., children were systematically removed from their homes and placed into the child welfare system; [41,42]). During these consultations, the community members' personal stories illustrated the complex intersections of sex, gender, stigma, substance use, colonial and structural violence, and other factors affecting care. These consultations provided insights into the interconnectedness of ongoing health crises and helped focus our research questions.

Several study co-investigators are the lead facilitators of HIV care and prevention in Manitoba, including The Manitoba HIV Program, which provides information, specialized care, and support to approximately 1500 PLHIV in Manitoba. Nine Circles Community Health Centre, The Manitoba Harm Reduction Network, Main Street Project, and Meeting the Moment are all key organizations and initiatives supporting underserved groups and guiding this research process.

For peer review only - http://bmjopen.bmj.com/site/about/guidelines.xhtml

After initial consultations, we assembled a RAC (Supplementary material 1) which includes an Indigenous Elder and Indigenous leaders; community members with lived experience in substance use, houselessness, and intergenerational trauma; and academic and scientific researchers of HIV, STBBI, and sex and gender-based analyses. The RAC guides the research team in research design and community-based engagement methods, data collection tools, culturally safe approaches to recruitment and data collection, review of preliminary findings, and knowledge translation strategies. The RAC's continued involvement ensures that the project is culturally safe for the diverse communities involved, the community's best interests are considered, and the results and findings are shared appropriately with stakeholders.

Additionally, we convened a PRT of community members with lived experience (Supplementary material 2). The purpose of the PRT's is to involve community members as co-researchers during the design of the recruitment strategy and data collection tools, data collection, data analysis, findings, and knowledge translation. Our approach to peer engagement focuses on building reciprocal relationships and sharing skills with peer members for each step in the research process, respectfully and safely. PRT meetings are held 2-3 times per month for two and a half hours. Peers are paid for their time and provided with transportation, childcare support, and skill-building activities.

Indigenous peoples make up 18% of the population of Manitoba (compared to 4.9% of the total population in Canada) [43], yet are overrepresented in new HIV diagnoses in Manitoba [8]. While not all participants will self-identify as Indigenous, we have received guidance from peers, RAC members and experts in community-based research to ensure our project remains culturally safe for all. Thus, a critical part of our research process centres around reciprocity. Reciprocity is a critical component of Indigenous culture that should be applied as an

For peer review only - http://bmjopen.bmj.com/site/about/guidelines.xhtml

overarching stance rather than a particular interaction [44]. Meaningful collaboration with peers, sharing skills (e.g., workshops, training sessions) and co-learning are components of our commitment to reciprocity. The RAC emphasized the need for reciprocity during data collection (with PLHIV) suggesting connecting participants with care (if they consent), offering participants other supports (e.g., peer and cultural supports, trauma counselling), honorarium, transportation, and childcare support. Beyond the data collection, members of the PRT, community members and leaders of community-based organizations are working with the research team to share the preliminary and final findings, mobilize and advocate for improvements and changes, and to develop actionable strategies from the findings. These strategies may include training courses to build capacity on related subjects (e.g., prevention and treatment of STBBIS and HIV), community-based dissemination strategies, and advocacy strategies for increased resources. Likewise, an Indigenous Cultural Advisor joined the team to provide a culturally safe support to participants, peers, and the research team. Even though the Cultural Advisor will not be able to advise on indigenous cultural safety for each distinct Indigenous group, she has extensive experience working in community organizations and can provide referrals to culturally safe resources. The Cultural Advisor also educates the research team about the history of Indigenous People's in Canada, Indigenous values, traditions, ways of living, and culturally safe research approaches.

Participants and Settings

Inclusion criteria for PLHIV include (Group 1):

1. Resident of Manitoba
2. 18 years or older
3. Diagnosed with HIV between 2018-2022

For peer review only - http://bmjopen.bmj.com/site/about/guidelines.xhtml

Inclusion criteria for service providers (Group 2):

1. Resident of Manitoba
2. Provide medical, social, or other services to PLHIV

The target sample size for this prospective study will be 15-25 PLHIV and 20-30 service providers who work with PLHIV.

Recruitment Process

Purposive sampling will be used due to the distinct eligibility requirements of the participant groups.

Group 1 Recruitment Process

Service providers from the Manitoba HIV Program locations (Health Sciences Centre HIV, Nine Circles Community Health Centre, and 7th Street Health Access) will be asked to support the recruitment of PLHIV. Service providers in these locations provide HIV-related support services and have established and trusted relationships with PLHIV. Recruitment materials will be shared with clinicians and service providers to discuss the study with potential participants. If participants are interested, they can contact the research team to discuss the study in more detail and confirm participation.

To support recruiting participants not linked to care, the research team will engage with community organizations supporting PLHIV who may use substances and may be experiencing unstable housing and mental health challenges. There has been a history of poor research relationships with PLHIV that have created experiences of mistreatment, exploitation, and disconnect between researchers and communities [45]. Therefore, spending time in community locations potential participants frequent is critical to enable meetings, gain trust, and build rapport. Site observations at community organizations will create opportunities for connections

For peer review only

and facilitate informal conversations with potential participants about the research. In line with the latest Manitoba HIV Program Report [8], the purposive sample in group 1 will ensure that at least half of the participants self-identify as women, followed by men and non-binary persons. Similarly, we will aim to include the experiences of Indigenous, PWID, and those with unstable housing experiences to accurately represent the people newly diagnosed in Manitoba [8].

Group 2 Recruitment Process

A list of organizations that support and provide services to PLHIV will be compiled to recruit service providers. The RAC and community partners will also be asked to suggest key informants. As with Group 1, a purposive sample will be used to identify a representative sample of participants who provide various services and identify as women, men, or non-binary. Likewise, the perspectives of Black, Indigenous, and People of Color (BIPOC) service providers will be considered for sampling.

Data Collection

To understand the structural determinants of health and the syndemics affecting PLHIV, we will collect quantitative and qualitative data focused on understanding HIV care and harm reduction services, social, structural and programmatic factors (Table 1).

Table 1. Data collection, sources, tools and variables collected in this study.

Source	Data type	Tool used	Variables
	Qualitative data	Interview Guide (supplementary material 3)	Personal description (Q1-3), experience during HIV diagnoses (Q4), services used for HIV care (Q5), barriers and facilitators to HIV care (Q6-8, Q11), impacts of Covid-19 (Q9), safety in health care settings (Q10), changes to HIV care (Q12), substance use patterns, types of substances, and drug injection practices (Q13-19), harm reduction

For peer review only - http://bmjopen.bmj.com/site/about/guidelines.xhtml

People living with HIV			services (Q20, Q22), substance use and violence (Q21), substance use and HIV care (Q23), Covid-19 and substance use (Q24), best ways to learn about prevention and treatment of STBBI's and harm reduction practices (Q25).
	Quantitative data	Sociodemographic and life circumstances survey (supplementary material 4)	Age (Questions[Q] 1-2), sex at birth, gender identity, sexual orientation, cultural background, marital status, language, religion, education level, place of residence, disabilities, income and housing (Q13-19), experiences with the criminal justice system, including discrimination and stigma because of HIV diagnosis (Q21-28), sexual and testing practices (Q29-36), substance use patterns, quantity, types of substances, and drug injection practices (Q37-43), experiences with violence including supports received for healing, and involuntary disclosing of HIV status (Q44-48).
		Childhood Trauma Questionnaire[46]	Emotional abuse, physical abuse, sexual abuse, emotional neglect, and physical neglect.
		Empowr-[47]	Self-efficacy, perceived power, optimism about and control over the future, and community optimism
Service providers	Qualitative data	Interview Guide (supplementary material 5)	Sex and gender, role, organization, and services provided (Q1-3), facilitators and barriers to HIV care in PLHIV (Q3-4), barriers specifically for PLHIV who use substances (Q5), the impact of Covid-19 on clients, organization, and personal (Q6-9), types of supports provided for clients (Q10-11), understanding of harm reduction (Q12), prevention of sexually transmitted and blood borne infections (Q13, Q18), creating safe environments (Q14), HIV policies in Manitoba (Q15-17).

For peer review only

The research team will greet potential participants who contact the research team by email or phone. Research members will follow a phone or email script to discuss inclusion, scheduling, accessibility, psychological, and emotional support for the data collection process. Psychological or emotional support available for this study will include mental health

For peer review only - http://bmjopen.bmj.com/site/about/guidelines.xhtml

counselling, cultural counselling, and peer support. Resource(s) will only be arranged and offered upon request, to ensure participant confidentiality. Participants will be provided information and access to health care related to HIV, other STBBIs, counselling, or primary health through referrals to community organizations. All the field research teams have training in trauma-informed care, harm reduction, mental health first aid, and non-violent crisis intervention. This safety process ensures that participants can receive support, as some of the themes explored may elicit emotional responses.

For PLHIV, data will be collected by the research associate using a combination of face-to-face in-depth semi-structured qualitative interviews and three surveys. The qualitative component will use an interview guide with 26 open-ended questions (Supplementary Material 3). The interview guide used for this study has been revised several times by the RAC, the PRT, and key stakeholders (e.g., service providers). Questions focus on experiences with acquiring and receiving an HIV diagnosis, connecting and receiving HIV care, discrimination and stigma, substance use, past trauma and violence, changes in life due to Covid-19, and knowledge and experiences with harm reduction services (Table 1). In addition, PLHIV will be invited to suggest changes to HIV care and broader health and social care in Manitoba. During the screening process, participants will be offered a PRT member as a co-interviewer and a cultural advisor accompanying the research associate. PRT members will be trained to conduct interviews and facilitate surveys. Many PRT members already have experience in community-based research, so they will be familiar with the data collection tools. At the suggestion of the RAC, we will schedule a break after the interviews to offer various activities (e.g., meditation and stretching exercises) and food (e.g., granola bars and juice boxes) that create a welcoming and comfortable environment for participants.

For peer review only - http://bmjopen.bmj.com/site/about/guidelines.xhtml

Three surveys will be conducted after the break, including a Participant Demographic and Life Circumstances Survey (Supplementary Material 4), Childhood Trauma Questionnaire (Short-Form) [46], and the Empowr- Making Decisions Survey [47]. The research team developed the Participant Demographics and Life Circumstances survey in consultation with key stakeholders and people with lived/living experiences. It enquires about PROGRESS (place of residence, race/ethnicity/culture/language, occupation, gender/sex, religion, education, socioeconomic status, and social capital) factors. Employing PROGRESS indicators provides an equity lens in highlighting health disparities [48]. These questions will allow describing the structural factors influencing participants' health. This survey also includes questions about substance use, harm reduction knowledge and practices, experiences with the criminal justice system, mental health challenges, and experiences of violence and trauma services (Table 1). The RAC and PRT extensively reviewed this survey to ensure readability and accuracy to capture the various structural factors PLHIV experience in Manitoba. The second questionnaire, the Childhood Trauma Questionnaire- Short [46], is a self-reported inventory that employs Likert scale responses to assess different types of childhood mistreatment and understand the role of historical trauma on life circumstances (Table 1). Finally, the Empowr- Making Decisions Survey [47] (revised shortened version) employs a Likert scale to understand factors related to resilience and decision-making (Table 1). These comprehensive measures will provide a holistic description of the interrelated experiences of HIV care, Covid-19, substance use, PROGRESS indicators, mental health factors, trauma, perceived personal resilience and decision-making of PLHIV in Manitoba. All interviews will take place in a private room to ensure participant confidentiality and researchers' safety. Participants will receive 50 CAD cash for their time at the end of the interview. Any transportation costs and childcare services will be reimbursed.

For peer review only - http://bmjopen.bmj.com/site/about/guidelines.xhtml

For peer review only

Interviews may be broken to several sessions based on participants circumstances and preference.

For service providers, semi-structured interviews will involve 19 open-ended questions (Supplementary Material 5 and Table 1). The interview guide used for service providers has been revised and edited several times by the RAC and key stakeholders. Questions focus on structural and social factors affecting HIV services and the lives of PLHIV. Service providers will also be asked to provide recommendations to improve HIV care. All interviews will occur over Zoom or Microsoft Teams following the University of Manitoba Virtual Platform Guidelines. Confidentiality and security in videoconferencing software will be ensured by only allowing authenticated users to join the meetings and saving meeting recordings in either a University of Manitoba OneDrive or locally to a University of Manitoba device with disk encryption enabled.

The research team will explain the study and obtain consent from all participants before the data collection. If written consent is not possible, research team members will collect verbal consent recorded at the beginning of the interview. Participants will take a copy of the consent form for their records. Sessions are expected to last between one to two and a half hours. Recordings will be encrypted and stored in a password-protected computer. In addition to recordings, research team members may take notes directly on the interview guide or in notebooks. After each interview, research team members will participate in debriefing sessions to increase study rigour.

Data Analysis

All qualitative and quantitative data will be disaggregated by sex and gender.

For peer review only - http://bmjopen.bmj.com/site/about/guidelines.xhtml

All qualitative data will be transcribed verbatim using Otter.ai and checked by the research team to ensure accuracy. Transcripts will be entered into NVivo© (version 12) and analyzed using grounded theory methods and sex- and gender-based analysis. Grounded theory provides an iterative framework in which data is collected and analyzed simultaneously by immersing the research members in the data through open coding, code analysis, and further exploration of new themes [49]. Three independent research team members with experience in qualitative analysis will conduct exploratory coding on an initial subset of the transcripts to establish a codebook for each group. Two independent coders will then establish intercoder reliability to increase rigour. Emerging codes will be grouped into larger categories, and themes will be developed to describe the data within a larger framework. During data analysis, peer debriefing will be used with PRT members to share views (e.g., processes, participant engagement) and ideas about codes and themes since this process reduces researcher bias [50]. Negative case analysis will also reduce researcher bias throughout the data analysis process. Finally, an audit trail of decisions through the data collection and analysis will be kept, increasing reproducibility.

Quantitative data from the three questionnaires will be reported using descriptive statistics. These surveys will complement qualitative data by providing information regarding social and structural inequity factors and provide a broader framework of the experiences of PLHIV in Manitoba. This study is part of a broader research that was describing the epidemiology of HIV in Manitoba (Manitoba HIV Program Report [8] and manuscripts in preparation). We collected from clinical charts sociodemographic and clinical information from 517 people newly diagnosed with HIV between 2018-2021. Data included >90 variables about age, sex, gender, race/ethnicity, rural/urban, substance use (drugs and route), other infectious

For peer review only - http://bmjopen.bmj.com/site/about/guidelines.xhtml

diseases and non-communicable diseases/conditions and treatments, primary care information, HIV- and Covid-19 related information, and follow-ups. Following the syndemic model [33,34], the data collected from clinical charts will provide information about concomitant conditions and how they interact, the quantitative and qualitative data collected prospectively and reported in this paper, will provide information about how people experience their conditions (social, structural and cultural factors unique to each person), and how HIV care and harm reduction services connect to people.

ETHICS AND DISSEMINATION

This project received ethics approval from the University of Manitoba Health Ethics Research Board (HS25572;H2022:218), First Nations Health and Social Secretariat of Manitoba, Nine Circles Community Health Centre, Shared Health Manitoba (SH2022:194), and 7th Street Health Access Centre. As some participants will self-identify as First Nations, Métis or Indigenous, the project will follow Indigenous data sovereignty principles. Among these are the First Nation principles of OCAP®, Manitoba Métis principles of OCAS, the Principles of Ethical Métis Research, and CARE Principles for Indigenous Data Governance, under the leadership of the RAC.

Findings will be disseminated through community-led knowledge translation strategies identified by participants, peers, community members, and community organizations; conference presentations, peer-reviewed journal articles, social media, workshops, and a dedicated webpage (www.alltogether4ideas.org).

DISCUSSION

For peer review only - http://bmjopen.bmj.com/site/about/guidelines.xhtml

Staggering inequities affect the lives of many groups across Canada [51]. To understand health inequities in Manitoba, it is crucial to contextualize the structural conditions that shape health outcomes. This contextualization would benefit from using social determinants of health and a syndemic framework in which health crises such as HIV, Covid-19, and substance use interact with each other and with inequitable social conditions to exacerbate adverse health outcomes in underserved groups [33–35]. In Manitoba, PWID, Indigenous peoples, and people experiencing houselessness are overrepresented in new HIV diagnoses [8]. Similarly, the onset of the Covid-19 pandemic accentuated pre-existing socioeconomic and structural factors, mental health concerns, violence, stigma and discrimination, increased sex and gender disparities, and reduced the resources for STBBI and harm reduction services [29,31]. Complementing qualitative and quantitative data collection with PLHIV and service providers can provide an in-depth understanding of barriers and facilitators to HIV care in Manitoba. Using in-depth semi-structured interviews and questionnaires will enable a broader approach to understanding and addressing Manitoba's various health crises and social inequities. This innovative study uses the World Health Organization Conceptual Framework for Action on the Social Determinants of Health and syndemic frameworks to understand the interrelated experiences of HIV care, Covid-19, substance use and harm reduction, trauma, violence, and other social and structural factors.

This project is driven by participatory engagement processes that centre on the knowledge and expertise of community members, organizations, and people with lived and living experiences to improve the lives, health, and well-being of PLHIV in Manitoba and beyond. Our sample is restricted to people living in metropolitan areas that account for approximately 80% of PLHIV (i.e., Winnipeg and Brandon) in Manitoba and individuals who can dedicate the time necessary for the data collection. However, our purposive recruitment

For peer review only - http://bmjopen.bmj.com/site/about/guidelines.xhtml

strategy to interview people with various backgrounds will ensure we have a representative sample and gain a more comprehensive understanding of the factors that affect the experiences of PLHIV.

Our project will shed light on the growing segment of people missing from the 95-95-95 UNAIDS and Canada's goal [9,10]. Thus, these results will help to advocate for policy and systemic changes to improve the health and well-being of PLHIV in Manitoba.

Acknowledgments: We are mainly thankful of Peer Research Team members Clara Dan, Joel Baliddawa, Lisa Patrick, Nikki Daniels, Robert Russell, Rebecca Murdock, Freda Woodhouse, Ann Favel, Nadia Mancheese, and Marj Schenkels, for their invaluable insights. We are also thankful to members of the Research Advisory committee including Dr. Jacqueline Gahagan, Elder Margaret Lavallee, Veda Koncan, Margaret Bryan, and Jody Jollimore. We thank Heather Pashe (Indigenous Cultural Advisor), some members of Sisters of Fire, Manitoba Harm Reduction Network, Main Street Project, Meeting the Moment, Nine Circles Community Health Centre, Street Connections, 7th Street Health Access Centre and Canadian AIDS Treatment Information Exchange (CATIE) for their ongoing guidance in this project. We are appreciative of all the social workers, nurses, physicians, health education facilitators and service providers in making this study possible. Lastly, thanks to Ms. Mariana Echeverri, communications coordinator of this project, Ms. Echeverri has designed the website, recruitment materials, and all communicational pieces regarding this research.

Author Contributions: The study concept and design were conceived by ZVR, MHB, and YK with input from CS, EV, LL, KM, KD, JS, KT, LM, LI, KK, MP, JB, AK, NP, TM, AM. EV will schedule, collect consent, interview, and complete surveys with participants. CS will

For peer review only - http://bmjopen.bmj.com/site/about/guidelines.xhtml

schedule, collect consent, interview, and complete surveys with service providers. EV and ZVR prepared the first draft of the protocol manuscript. CS, LL, KM, KD, JS, KT, LM, LI, KK, MP, JB, AK, NP, TM, AM, YK, members of the Research Advisory Committee and the Peer Research Team reviewed, edited and revised it critically for important intellectual content.

Competing Interest Statement: There are no competing interests to report.

Funding: This work was supported by the Canadian Institute for Health Research (Grant number EG5-179453), The Manitoba Medical Service Foundation (grant number: 2021-13), The James Farley Memorial Fund. This research was also supported, in part, by the Canada Research Chairs Program for ZVR (Award # 950-232963). Production of this manuscript has been made possible in part through a financial contribution of the Public Health Agency of Canada to the National Collaborating Centre for Infectious Diseases. The views expressed herein do not necessarily represent the views of the Agency.

Data Availability Statement: Data for this study is not available yet since this is a protocol paper. The study interview guides and questionnaires are available in the supplementary materials section.

For peer review only

References

1 Govender RD, Hashim MJ, Khan MA, *et al.* Global Epidemiology of HIV/AIDS: A Resurgence in North America and Europe. *J Epidemiol Glob Health* 2021;**11**:296–301. doi:10.2991/jegh.k.210621.001

2 Coovadia HM, Hadingham J. HIV/AIDS: global trends, global funds and delivery bottlenecks. *Glob Health* 2005;**1**:13. doi:10.1186/1744-8603-1-13

3 Biesma RG, Brugha R, Harmer A, *et al.* The effects of global health initiatives on country health systems: a review of the evidence from HIV/AIDS control. *Health Policy Plan* 2009;**24**:239–52. doi:10.1093/heapol/czp025

4 Hanefeld J. How have Global Health Initiatives impacted on health equity? *Promot Educ* 2008;**15**:19–23. doi:10.1177/1025382307088094

5 Shannon K, Crago A-L, Baral SD, *et al.* The global response and unmet actions for HIV and sex workers. *Lancet Lond Engl* 2018;**392**:698–710. doi:10.1016/S0140-6736(18)31439-9

6 Haddad N, Robert A, Weeks A, *et al.* HIV in Canada—Surveillance Report, 2018. *Can Commun Dis Rep* 2019;**45**:304–12. doi:10.14745/ccdr.v45i12a01

7 Duncan KC, Reading C, Borwein AM, *et al.* HIV Incidence and Prevalence Among Aboriginal Peoples in Canada. *AIDS Behav* 2011;**15**:214–27. doi:10.1007/s10461-010-9792-y

8 Manitoba HIV Program. Manitoba HIV Program Report 2018-2021. 2022.https://mbhiv.ca/wp-content/uploads/2022/11/Report-MB-HIV-Program-FINAL-Nov-30-2022-Reduced-size.pdf

9 UNAIDS. Understanding Fast-Track Accelerating Action To End the Aids Epidemic by 2030. 2015.https://www.unaids.org/sites/default/files/media_asset/201506_JC2743_Understanding_FastTrack_en.pdf (accessed 16 Aug 2022).

10 Public Health Agency of Canada. Canada takes action by endorsing global declaration on Undetectable = Untransmittable (U=U). 2022.https://www.canada.ca/en/public-health/news/2022/07/canada-takes-action-by-endorsing-global-declaration-on-undetectable--untransmittable-uu.html (accessed 9 Mar 2023).

11 Center for Disease Control and Prevention (CDC). Understanding the HIV Care Continuum. 2017.https://www.cdc.gov/hiv/pdf/library/factsheets/cdc-hiv-care-continuum.pdf (accessed 9 Aug 2022).

12 Tso LS, Best J, Beanland R, *et al.* Facilitators and barriers in HIV linkage to care interventions: a qualitative evidence review. *AIDS Lond Engl* 2016;**30**:1639–53. doi:10.1097/QAD.0000000000001101

For peer review only - http://bmjopen.bmj.com/site/about/guidelines.xhtml

13 Dombrowski J. Linkage to Care. Natl. HIV Curric. 2020.https://www.hiv.uw.edu/go/screening-diagnosis/linkage-care/core-concept/all (accessed 10 Aug 2022).

14 Hall BJ, Sou K-L, Beanland R, *et al.* Barriers and Facilitators to Interventions Improving Retention in HIV Care: A Qualitative Evidence Meta-Synthesis. *AIDS Behav* 2017;**21**:1755–67. doi:10.1007/s10461-016-1537-0

15 Argento E, Strathdee SA, Goldenberg S, *et al.* Violence, trauma and living with HIV: Longitudinal predictors of initiating crystal methamphetamine injection among sex workers. *Drug Alcohol Depend* 2017;**175**:198–204. doi:10.1016/j.drugalcdep.2017.02.014

16 Scully EP. Sex Differences in HIV Infection. *Curr HIV/AIDS Rep* 2018;**15**:136–46. doi:10.1007/s11904-018-0383-2

17 Everett BG. Sexual Orientation Disparities in Sexually Transmitted Infections: Examining the Intersection Between Sexual Identity and Sexual Behavior. *Arch Sex Behav* 2013;**42**:225–36. doi:10.1007/s10508-012-9902-1

18 Ostrach B, Singer M. At special risk: Biopolitical vulnerability and HIV/STI syndemics among women. *Health Sociol Rev* 2012;**21**:258–71. doi:10.5172/hesr.2012.21.3.258

19 Statistics Canada. Family Violence in Canada: A Statistical Profile. 2011.https://www150.statcan.gc.ca/n1/pub/85-224-x/85-224-x2010000-eng.pdf

20 Tutty LM, Nixon KL. #MeToo on the Canadian Prairies: Raising Awareness of Sexual Assaults and Mental Health in Women Abused by Intimate Partners. *Violence Women* 2022;**28**:1398–419. doi:10.1177/10778012211032699

21 Tutty LM, Radtke HL, Ateah CA, *et al.* The Complexities of Intimate Partner Violence: Mental Health, Disabilities, and Child Abuse History for White, Indigenous, and Other Visible Minority Canadian Women. *J Interpers Violence* 2021;**36**:1208–32. doi:10.1177/0886260517741210

22 Tutty LM, Radtke HL, Thurston WEB, *et al.* The Mental Health and Well-Being of Canadian Indigenous and Non-Indigenous Women Abused by Intimate Partners. *Violence Women* 2020;**26**:1574–97. doi:10.1177/1077801219884123

23 Nixon KL, Tutty LM, Radtke HL, *et al.* Protective Strategies of Mothers Abused by Intimate Partners: Rethinking the Deficit Model. *Violence Women* 2017;**23**:1271–92. doi:10.1177/1077801216658978

24 Chenneville T, Gabbidon K, Hanson P, *et al.* The Impact of COVID-19 on HIV Treatment and Research: A Call to Action. *Int J Environ Res Public Health* 2020;**17**:E4548. doi:10.3390/ijerph17124548

For peer review only - http://bmjopen.bmj.com/site/about/guidelines.xhtml

25 Tsamakis K, Tsiptsios D, Ouranidis A, *et al.* COVID-19 and its consequences on mental health (Review). *Exp Ther Med* 2021;**21**:244. doi:10.3892/etm.2021.9675

26 Salmon S, Taillieu TL, Fortier J, *et al.* Pandemic-related experiences, mental health symptoms, substance use, and relationship conflict among older adolescents and young adults from Manitoba, Canada. *Psychiatry Res* 2022;**311**:114495. doi:10.1016/j.psychres.2022.114495

27 Almeida M, Shrestha AD, Stojanac D, *et al.* The impact of the COVID-19 pandemic on women's mental health. *Arch Womens Ment Health* 2020;**23**:741–9. doi:10.1007/s00737-020-01092-2

28 Castellini G, Tarchi L, Cassioli E, *et al.* Attachment Style and Childhood Traumatic Experiences Moderate the Impact of Initial and Prolonged COVID-19 Pandemic: Mental Health Longitudinal Trajectories in a Sample of Italian Women. *Int J Ment Health Addict* Published Online First: 17 March 2022. doi:10.1007/s11469-022-00798-x

29 Manitoba Harm Reduction Network, The National Collaborating Centre for Infectious Diseases. Report of an Evaluation of Manitoba Harm Reduction Network Services in the Context of the COVID-19 Pandemic. 2022.https://nccid.ca/wp-content/uploads/sites/2/2022/02/MHRN-report_2022_Final.pdf

30 European Monitoring Centre for Drugs and Drug Addiction, editor. Harm reduction: evidence, impacts and challenges. 2010.https://www.emcdda.europa.eu/system/files/publications/555/EMCDDA-monograph10-harm_reduction_final_205049.pdf

31 Public Health Agency of Canada. Survey of the impact of COVID-19 on access to STBBI-related health services including harm reduction services among people who use drugs or alcohol in Canada. 2021.https://health-infobase.canada.ca/datalab/covid-19-stbbi-services.html (accessed 22 Jul 2022).

32 Epidemiology and Surveillance Unit, Government of Manitoba. Substance Related Harms: Epidemiologist's Summary. Ment. Health Community Wellness. 2022.https://www.gov.mb.ca/mh/index.html (accessed 9 Mar 2023).

33 Singer M. A dose of drugs, a touch of violence, a case of AIDS: Conceptualizing the Sava Syndemic. *Free Inq Creat Sociol* 2000;**28**:13–24.

34 Singer M. *Introduction to Syndemics: A Critical Systems Approach to Public and Community Health | Wiley*. https://www.wiley.com/en-us/Introduction+to+Syndemics%3A+A+Critical+Systems+Approach+to+Public+and+Community+Health-p-9780470472033 (accessed 27 Jul 2022).

For peer review only - http://bmjopen.bmj.com/site/about/guidelines.xhtml

35 World Health Organization. A conceptual framework for action on the social determinants of health. World Health Organization 2010. https://apps.who.int/iris/handle/10665/44489 (accessed 24 Mar 2023).

36 Andermann A. Outbreaks in the age of syndemics: New insights for improving Indigenous health. *Can Commun Dis Rep* 2017;**43**:125–32. doi:10.14745/ccdr.v43i06a02

37 Else-Quest NM, Hyde JS. Intersectionality in Quantitative Psychological Research: II. Methods and Techniques. *Psychol Women Q* 2016;**40**:319–36. doi:10.1177/0361684316647953

38 Reason P, Bradbury H, editors. *The Sage handbook of action research: participative inquiry and practice*. 2nd ed. London ; Thousand Oaks, Calif: : SAGE Publications 2008.

39 Allen W. Participatory action research provides for multiple benefits - Learning for Sustainability. Learn. Sustain. 2016.https://learningforsustainability.net/post/par/ (accessed 25 Jul 2022).

40 Organizing engagement. Participatory Action Research and Evaluation. Organ. Engagem. 2019.https://organizingengagement.org/models/participatory-action-research-and-evaluation/ (accessed 10 Aug 2022).

41 Johnston P. *Native children and the child welfare system*. Toronto [Ont.]: : Canadian Council on Social Development in association with James Lorimer & Co. 1983. https://www.deslibris.ca/ID/413616 (accessed 4 Apr 2023).

42 National Collaborating Centre for Aboriginal Health. Indigenous Children and the Child Welfare System in Canada. 2017.https://www.nccih.ca/docs/health/FS-ChildWelfareCanada-EN.pdf

43 Statistics Canada. Focus on Geography Series, 2016 Census. 2017.https://www12.statcan.gc.ca/census-recensement/2016/as-sa/fogs-spg/Facts-PR-Eng.cfm?TOPIC=9&LANG=Eng&GK=PR&GC=46 (accessed 27 Jul 2022).

44 McGregor HE, Marker M. Reciprocity in Indigenous Educational Research: Beyond Compensation, Towards Decolonizing. *Anthropol Educ Q* 2018;**49**:318–28. doi:10.1111/aeq.12249

45 Logie C. *Working with Excluded Populations in HIV: Hard to Reach or Out of Sight?* Cham: : Springer International Publishing 2021. doi:10.1007/978-3-030-77048-8

46 Bernstein D, Stein J, Newcomb M, *et al.* Development and validation of a brief screening version of the Childhood Trauma Questionnaire. 2003;**27**:169–90. doi://doi.org/10.1016/S0145-2134(02)00541-0

For peer review only - http://bmjopen.bmj.com/site/about/guidelines.xhtml

47 Rogers ES, Ralph RO, Salzer MS. Validating the empowerment scale with a multisite sample of consumers of mental health services. *Psychiatr Serv Wash DC* 2010;**61**:933–6. doi:10.1176/ps.2010.61.9.933

48 O'Neill J, Tabish H, Welch V, *et al.* Applying an equity lens to interventions: using PROGRESS ensures consideration of socially stratifying factors to illuminate inequities in health. *J Clin Epidemiol* 2014;**67**:56–64. doi:10.1016/j.jclinepi.2013.08.005

49 Charmaz K. *Constructing Grounded Theory A Practical Guide Through Qualitative Analysis*. SAGE Publications 2006.

50 Heilman J, Figg C, Wenrick M, *et al.* Implications and Benefits of a Long-Term Peer Debriefing Experience on Teacher Researchers. *Brock Educ J Educ Res Pract* 2010;**19**:20–35. doi:10.26522/brocked.v19i1.118

51 Public Health Agency of Canada. Key health inequalities in Canada: a national portrait. 2018.http://epe.lac-bac.gc.ca/100/201/301/weekly_acquisitions_list-ef/2019/19-06/publications.gc.ca/collections/collection_2019/aspc-phac/HP35-109-2018-1-eng.pdf (accessed 17 Aug 2022).

For peer review only

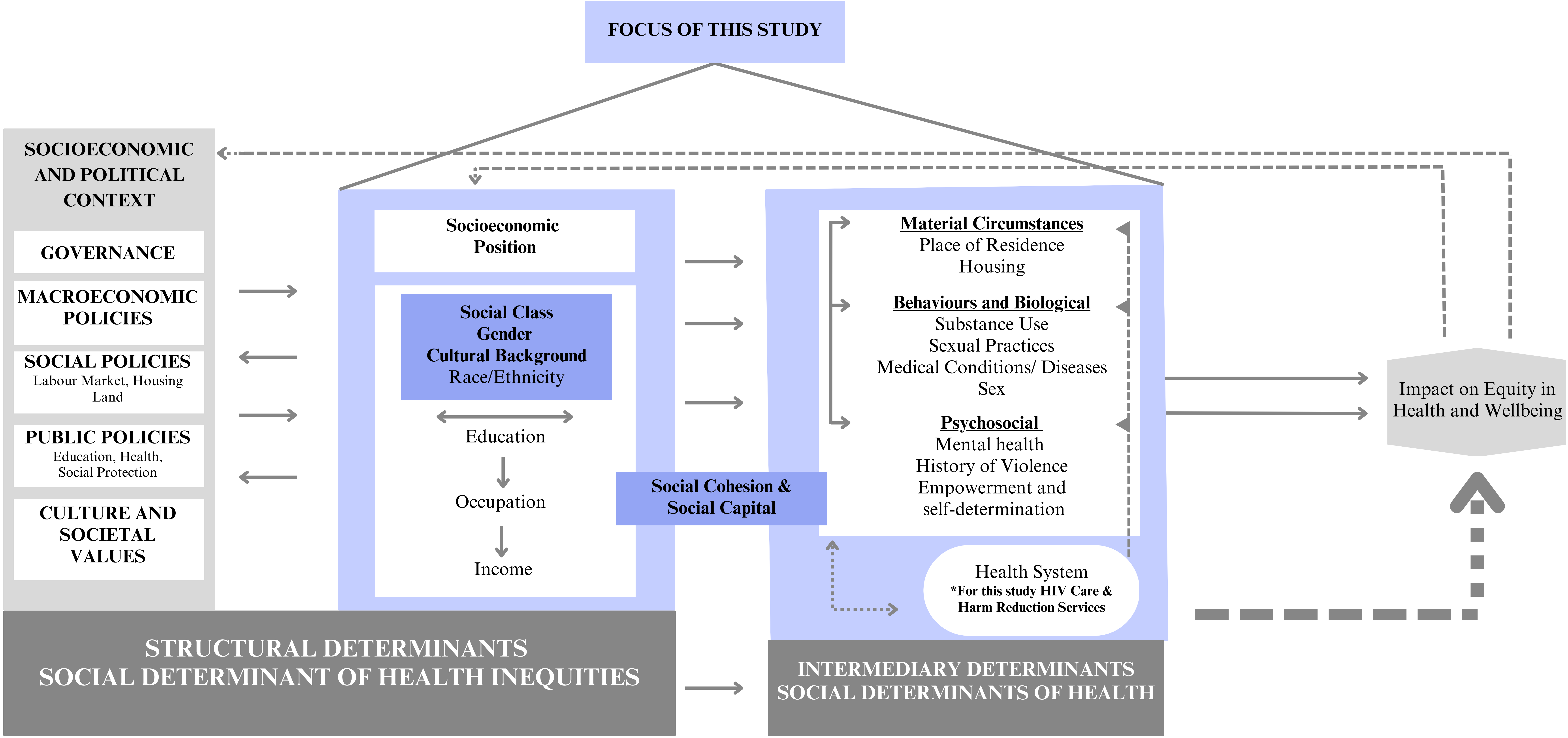
FOCUS OF THIS STUDY
SOCIOECONOMIC AND POLITICAL CONTEXT
GOVERNANCE
MACROECONOMIC POLICIES
SOCIAL POLICIES
Labour Market, Housing Land
PUBLIC POLICIES
Education, Health, Social Protection
CULTURE AND SOCIETAL VALUES
Socioeconomic Position
Social Class
Gender
Cultural Background
Race/Ethnicity
Education
Occupation
Income
Social Cohesion & Social Capital
Material Circumstances
Place of Residence
Housing
Behaviours and Biological
Substance Use
Sexual Practices
Medical Conditions/ Diseases
Sex
Psychosocial
Mental health
History of Violence
Empowerment and self-determination
Health System
*For this study HIV Care & Harm Reduction Services
Impact on Equity in Health and Wellbeing
STRUCTURAL DETERMINANTS
SOCIAL DETERMINANT OF HEALTH INEQUITIES
INTERMEDIARY DETERMINANTS
SOCIAL DETERMINANTS OF HEALTH

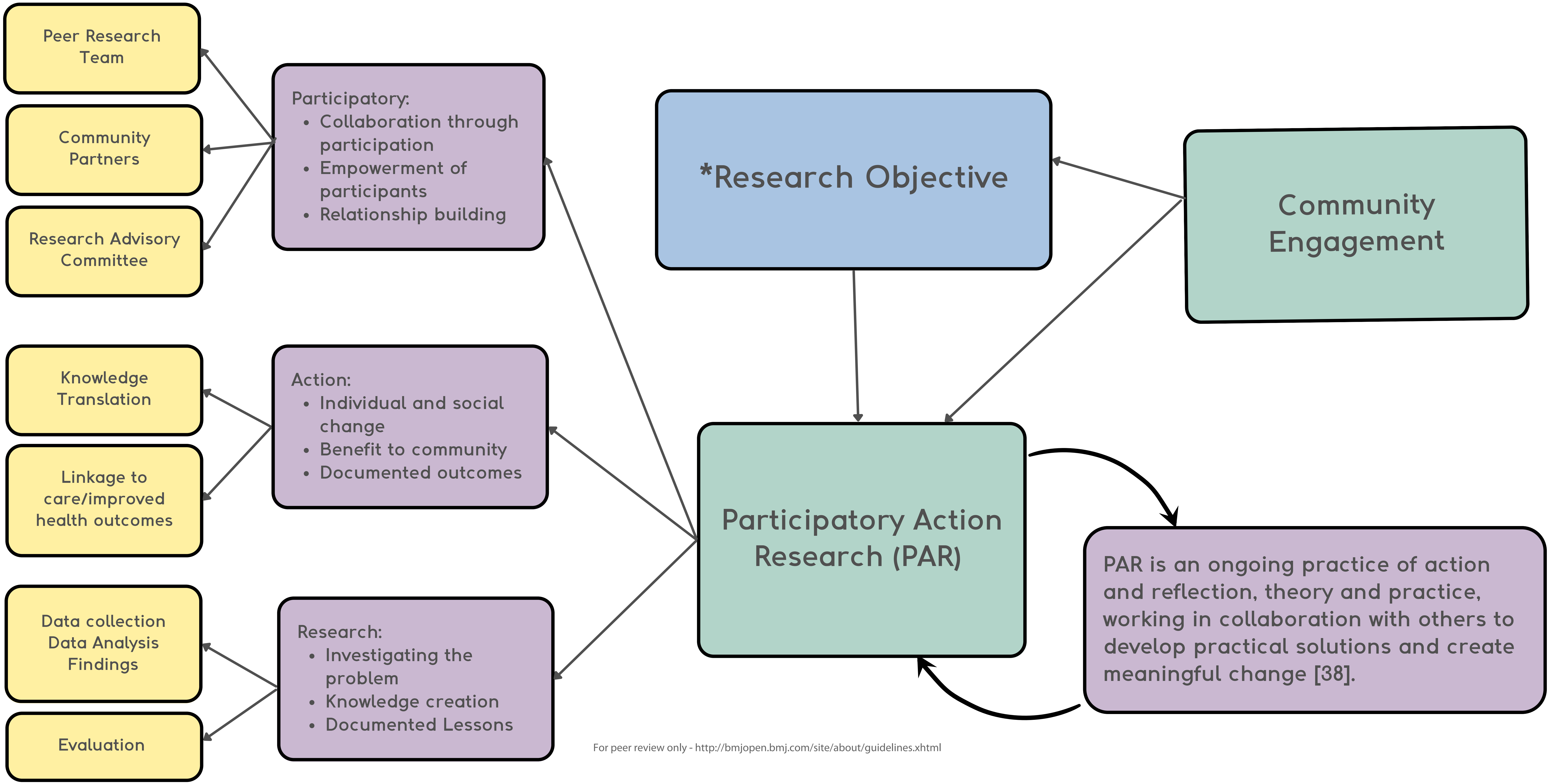
Peer Research Team
Community Partners
Research Advisory Committee
Knowledge Translation
Linkage to care/improved health outcomes
Data collection Data Analysis Findings
Evaluation
Participatory:
• Collaboration through participation
• Empowerment of participants
• Relationship building
Action:
• Individual and social change
• Benefit to community
• Documented outcomes
Research:
• Investigating the problem
• Knowledge creation
• Documented Lessons
*Research Objective
Community Engagement
Participatory Action Research (PAR)
PAR is an ongoing practice of action and reflection, theory and practice, working in collaboration with others to develop practical solutions and create meaningful change [38].

Terms of Reference for the Research Advisory Committee

Background:

In Manitoba, injection drug use is the most common mode of HIV transmission since 2018. Those who inject drugs are more likely to test positive for HIV, syphilis and Hepatitis C, often at the same time. This is called a "syndemic" (many epidemics at the same time). For the last three years, syphilis infections have become rampant in Manitoba. If not found and treated, syphilis can cause devastating infection, including transmission to newborns. In addition to HIV and syphilis there are 11 other infections transmitted by sexual activity or blood products (called STBBI).
For some people, a convergence of socio-economic factors, mental health concerns, violence and exploitation, is driving a syndemic of new HIV and other STBBI cases, with disproportionate representation of women who inject drugs. Methamphetamine use is growing in Manitoba and is known to affect access to care and treatment. These determinants of health and disparities are exacerbated by COVID-19 related public health measures, as resources for testing and contact tracing for Covid-19 have resulted in decreased capacity for service providers to respond to STBBI and distribute harm reduction services. It is unknown how both biological sex differences and gender intersect with living conditions including experiences of violence and injection drug use.

This study seeks to know:

1. How many people in Manitoba (by sex, gender and other identifiers) living with HIV have other sexually transmitted and blood-borne infections (STBBI), before and during Covid-19?
2. Of those living with HIV, whom are most at risk of acquiring other STBBI, and why?
3. What are the barriers and gaps that put people at risk for infection and prevent them from getting appropriate care and treatment before and during Covid-19?
4. What are the resilience factors and promising practices that are associated with successful engagement of people living with HIV and who inject methamphetamine that endured during the Covid-19 pandemic?

Gaining an in-depth understanding of the experiences of women, men and non-binary persons living with HIV who use and don't use drugs can help us identify unique gaps as well as resilience factors that can inform and improve the Manitoba HIV Program, and result in tailored testing and appropriate clinical and program responses.

Principal Investigators:

Dr. Zulma Rueda. University of Manitoba. Email: zulma.rueda@umanitoba.ca
Dr. Yoav Keynan University of Manitoba. Email: yoav.keynan@umanitoba.ca
Ms. Margaret Haworth-Brockman, University of Manitoba. Email: Margaret.Haworth-Brockman@umanitoba.ca

Advisory Committee's Role and Purpose

The Advisory Committee will advise the research team on the following aspects of the project:

- ✓ Research approach and community-based methods
- ✓ Guidance the implementation of research.
- ✓ Promotion of the research study.

For peer review only

- ✓ Advice on community engagement plans.
- ✓ Interpretation of findings.
- ✓ Help to identify and strategize any potential solutions to community issues that may arise.
- ✓ At all times, strictly maintain confidentiality for all aspects of the research project as outlined in the University of Manitoba *Access and Privacy policy* and *Access and Privacy Procedure.*

- ✓ **Input and advice from the Advisory Committee will help to ensure that**:
  - ✓ The project/study is culturally appropriate for the diverse communities involved.
  - ✓ The research team conducts the project in the communities' best interests.
  - ✓ The research results and knowledge will be shared appropriately with communities and stakeholders.
  - ✓ Action plans associated with the research receive community input and are appropriate to community needs and concerns.

All Advisory Committee members will be eligible for compensation for their time involved. We expect there to be about 5-7 meetings over 12 months, with one or two meetings being in the early stages of the project development.

Responsibilities from the research team:

1. Ensure the ethical conduct of the research study.
2. Provide accurate information related to the project.
3. Investigate and answer the suggestions of the research advisory committee.

Disclosures:

- ✓ All member will declare any real or perceived conflict of interest at the outset of any meeting for management in accordance with the University of Manitoba *Conflicts of Interest Policy and Conflict of Interest Procedures.*
- ✓ All research project conducted within the University of Manitoba is governed by the *Responsible Conduct of Research Policy, Responsible Conduct of Research – Code of Research Ethics Policy, Responsible Conduct of Research – Investigation Procedure.*
- ✓ This research is funded by the Canadian Institute of Health Research; therefore, we follow and adhere to the *Tri-Agency Framework: Responsible Conduct of Research, including the framework and policies of Chapter 9: Research Involving the First Nations, Inuit and Métis Peoples of Canada.*

Term

This research is initially funded for one year.

Memberships

The Committee will be composed by the following members, in addition to the 3 principal investigators. Our meetings will be opened in a Good Way with the help of an Elder.

The number and composition of members comprises of:

1. Five or six community members – we are actively recruiting advisors now. We are inviting members who include:

For peer review only

a. Person who lives with HIV
b. Person who has current or former experience with injection drug use
c. Women or female who have experienced sexual assault, sexual violence, experiencing violence or sexual exploitation
d. Person who is experiencing homelessness or who are unsheltered
e. Person who identify themselves as 2SLGBTQQIA+
f. Person who belong to Indigenous population or racialized communities

2. Elder Margaret Lavallee, Elder in Residence, Ongomiizwin
3. Julianne Sanguins. Adjunct Scientist at the Manitoba Centre for Health Policy, and Assistant Professor at the Department of Community Health Sciences, University of Manitoba.
4. Tara Faye Myran, Metis Community member.
5. Ann Favel, Person with lived experience. Member of Sisters of Fire
6. Jaqueline Flett, Person with lived experience. Member of Sisters of Fire
7. Marj Schenkels, Person with lived experience. Member of Sisters of Fire
8. Nikki Daniels. Community member with lived experience.
9. Jody Jollimore. Executive Director at CATIE
10. Margaret Bryan. Manitoba Harm Reduction Network
11. Jacqueline Gahagan. Associate Vice-President, Research, Mount Saint Vincent University. Halifax, Nova Scotia.
12. Adrienne FA Meyers, Associate Director of the JC Wilt Infectious Diseases Research Centre
13. Neora Pick, Clinical Professor in the Infectious Diseases division of the Department of Medicine at the University of British Columbia, and Medical Director at the Oak Tree Clinic.
14. Veda Koncan, Projects Manager, Manitoba Harm Reduction Network
15. Chantal Daniels, Elder Support, Indigenous Institute of Health & Healing, Ongomiizwin

Meeting frequency

Meetings will be called by the Project Manager.
The committee will meet for five or seven 2-hour meetings either in person in Winnipeg or via teleconference/web-based technology for members residing outside Winnipeg.

Sitting fees

Research team will provide $20 CAD/per hour honorariums for people who are eligible for honorarium
Research team will cover costs for transportation for people residing in Winnipeg

Contact:

Dr. Zulma Rueda. University of Manitoba. Email: zulma.rueda@umanitoba.ca
Dr. Yoav Keynan University of Manitoba. Email: yoav.keynan@umanitoba.ca
Ms. Margaret Haworth-Brockman, University of Manitoba. Email: Margaret.Haworth-Brockman@umanitoba.ca

For peer review only

Cheryl Sobie. Research project coordinator. University of Manitoba. Email: Cheryl.Sobie@umanitoba.ca

For peer review only

Peer Terms of Reference

Purpose:

We want to engage people with lived experience as peers in our project and work collaboratively to ensure all aspects of the research are shaped and guided by the perspectives of peers with lived experience. Peer engagement will inform the entire research process, from conducting research interviews, to skill sharing, workshops, and developing educational materials to share the results of our research. We recognize the unique knowledge and expertise of community members with lived experience, and we seek your guidance and input for our research study.

Background Information:

We are peers, clinicians and researchers who want to understand infections transmitted by sex or blood in people living with HIV, who may use drugs, as well as why people living with HIV are not getting the care they need. We are interested in knowing which factors, barriers and gaps prevent people from accessing care. We will also look for resilience factors that keep people linked to care.

We are interested in better understanding the impact of the COVID-19 pandemic and how it may have affected people newly diagnosed with HIV (between 2018-2021) in accessing health care and other resources. We want to know things such as:

- What was it like navigating the health care system during the pandemic?
- What are ways we can make things better for people to be linked to health care?
- What things are working well for people that keep them going to get health support and resources before and during the COVID-19 pandemic?

Project Time Period:

July 2022 – March 2023

Peer Roles available:

1. Peer Research Advisory Committee Member
2. Peer Support Worker
3. Peer Group Team Member

Role (1) Peer Research Advisory Committee Member (see Research Advisory Committee Terms of Reference Document). Key items to note:

How does this work?
You will join our committee which includes peers like you and some researchers. Together you will advise the research team about the best ways to:

- involve other people in the research study (using posters, or word of mouth, or in certain places);

For peer review only

- pay or compensate them for their time (for example, taxi coupons, bus passes, cash, or pre-paid VISAs);
- questions that are appropriate in interviews;
- how to understand what we find from the results;
- how to share the results of the study with the community;
- other education that the community or health care providers need to know. There may be other things you provide advice on.

Meeting frequency:

The Research Advisory Committee will meet for five or seven 2-hour meetings either in person in Winnipeg or via teleconference/web-based technology for members residing outside Winnipeg.

Compensation:

The research team will provide $20 CAD/per hour honorarium for peer research advisory committee members. Research team will cover costs for transportation (bus fare, taxi) for people residing in Winnipeg to attend the meeting.

<u>Role (2) Peer Support Worker:</u>

How does this work?

- Provide peer support for participants (people living with HIV) during research interview
- You will sit in the interview with participant and research team member to provide support as needed
- When we are scheduling interviews, we will ask participants if they would like a peer support worker at their interview
- Must sign confidentiality agreement and maintain confidentiality at all times
- We plan to interview 20-30 people; we are unsure at this point how many will require support
- The interview will take place in community settings such as Nine Circles, HSC HIV Clinic or another community location as determined by the participant and research team member

Time Required:

- 2-3 hours per interview session
- We anticipate interviews will take place between the end of August - October 2022

<u>Role (3) Peer Research Team Member</u>

How does this work?

- The purpose of the peer research team is to bring together community members with lived experience to work together to conduct research (interviews, data analysis, review findings), develop ideas for how we will share the results of our research, review comments from the advisory committee, and provide guidance and direction on the research for recruitment, community engagement, etc.
- The peer team is separate from the advisory board; however, a peer group member may also sit on the advisory board.
- The work of the peer team will include items such as:
    - Develop research skills and conduct research interviews, support data analysis and review findings of research
    - Discuss issues raised by the advisory board
    - Develop ideas and collaborate to prepare educational material for the community based on the results and findings of the research
    - To learn from each other either by skill sharing (i.e., doing computer courses provided by university, learning new skills related to grant writing/proposal development, learning technologies such as Zoom, etc.); we want to hear from YOU what you want to learn about
    - To advocate and share with the public what it is that is needed in Manitoba to break the stigma of HIV and drug use
- The peer team will consist of ten community members with lived experience
- Members will be selected by members that want to participate. If too many people apply, we will narrow out the group by selecting someone from each of the criteria in line with the research

Meetings:

<u>Meeting Frequency & Location</u>
The peer team will meet every two to three weeks for two hours. We will meet in person wherever possible at the University of Manitoba Bannatyne campus, or outside at a park. Should things change depending on the COVID-19 situation and safety guidelines we may have to meet online using Zoom or Teams.

<u>Comfort & Safety</u>
We will create a safe space for all group members to participate in, which includes psychological safety and cultural safety. Members can participate to the maximum comfortable amount.

We reject any form of discrimination or racism. We are committed for a safe and inclusive environment for everyone to participate. We do not judge each other and will respect each other at all times. We are committed to making your participation as accessible as possible. If there is anything you need from us to support you, please let us know.

<u>Activities</u>

For peer review only

Meetings will focus on relationship building, skill sharing, and project discussions. Once the data collection is completed, we will then turn our attention to creating educational materials to share the results of the research.

In addition to the research and skill sharing activities we want to incorporate fun activities or cultural activities such as beading, drum circles, bringing an Elder, playing games, sharing food together, music, etc.

Supplies

At each meeting:

- We will provide light lunches or snacks and drinks
- Cover any training and education costs
- Provide supplies for making educational materials
- Bring materials required for any activities as discussed

Compensation:

The peer group role will be compensated with an honorarium in the amount of $20/hour cash and bus tickets for transportation will be provided. Childcare will be compensated as needed $10 per hour. No money other than honorarium's will be received by peer members.

- ✓ **Confidentiality**- all team members will respect the confidentiality of other team members and any items discussed within group meetings.
- ✓ All members will declare any real or perceived conflict of interest at the outset of any meeting for management in accordance with the University of Manitoba *Conflicts of Interest Policy and Conflict of Interest Procedures.*
- ✓ All research project conducted within the University of Manitoba is governed by the *Responsible Conduct of Research Policy, Responsible Conduct of Research – Code of Research Ethics Policy, Responsible Conduct of Research – Investigation Procedure.*
- ✓ This research is funded by the Canadian Institute of Health Research; therefore, we follow and adhere to the *Tri-Agency Framework: Responsible Conduct of Research, including the framework and policies of Chapter 9: Research Involving the First Nations, Inuit, and Métis Peoples of Canada.*

For peer review only

Covid-19 Pandemic Concealing a Syndemic of Concern: Sex, Gender, Methamphetamine and Sexually Transmitted and Blood Borne Infections in People Living with HIV in Manitoba

Participant Interview Question Guide

To be completed by facilitator	Facilitator Name		
Participant ID:			
Participating Organization:			
Location:			
Interview date:			
Interview time:	Start	End	Duration
Confirmation of consent signed:			

Thank you for agreeing to speak with me today. My name is [INSERT] and my pronouns are INSERT] and this is my colleague [introduce themselves]. My role with this project is as a [Research Assistant, Research Associate, Research Coordinator]. Is there anything you need to do to prepare yourself before we get started? We anticipate the interview and short surveys will take between 1-2 hours to complete. We can take a break during the question portion of this interview if you need to, and we will take a short break after the interview before we begin the surveys. Please let me know at any time if you need to step away for a few minutes.

[NOTE: This next section only applies to people who will not have booked an interview appointment and are a walk-up participant]

Before I explain the purpose and what we will do today I want to check in with you about support we can offer you today and I have three questions I would like to ask you:

1. ***Would you be interested in having a peer support to sit with you for your interview? The purpose of a peer support is only to provide emotional support and they will maintain confidentiality of anything we discuss today.***
2. ***Would you be interested in having cultural support? There is an Elder available who you can speak with at any time if you need a break during the interview, at the break between the interview and surveys or at the end of the interview.***

For peer review only - http://bmjopen.bmj.com/site/about/guidelines.xhtml

Covid-19 Pandemic Concealing a Syndemic of Concern: Sex, Gender, Methamphetamine and Sexually Transmitted and Blood Borne Infections in People Living with HIV in Manitoba

3. ***Are you interested in speaking with a psychological support person at the break or after your interview today? They are available anytime if you need to stop the interview to debrief or check in with them.***

Before we start the formal interview questions, I would like to remind you that we are here to speak about your experiences in finding and receiving HIV and STBBI-related health care and services. Our aim is to improve HIV and other services for everyone. We are interviewing people with many stories and, perhaps, some questions may not relate to your life experiences. We appreciate your responses since many of these questions are personal. There are some questions about substance use that may or may not apply to you. The reason we are asking these questions is because our research has shown that in Manitoba, the main way people get HIV is through injection drug use but that is by no means the only way people get HIV. We understand that some people may feel shame or stigma about having HIV or using drugs, which is why we have tried to create a safe and comfortable space to listen to your experiences. It is completely up to you if you want to answer the questions or not.

You have been asked to participate in this interview as you:

1) *Are 18 years or older*
2) *Have received a diagnosis for HIV between 2018-2021*
3) *Are a resident of Manitoba*

Do all of these statements apply to you? ***Y / N***

[If no, relay that they are not eligible to participate in the study. Thank them for their time, and end interview].

For the purpose of our discussion, STBBI services may include testing, care and counselling for HIV, Hepatitis C, syphilis, other sexually transmitted infections like Chlamydia, or gonorrhoea.

We take your information seriously which is why we also want to remind you that everything you share today will be kept confidential. This means that we will not disclose any information you share with us about drug use, engaging in sex work or anything else that could be perceived as "criminal" with authorities. The only reason we would ever have to break confidentiality is if there is a direct risk/danger to your health and safety or that of someone who is under 18. We would like to record this interview to make sure the information is accurate. This recording will be kept in a secure location and destroyed after this study is over. This recording will not be shared.

For your participation today, we will give you a $50 honorarium in the form of cash and bus fare or money to cover a taxi ride to attend the interview. Additionally, we have several items that you can choose from as a gift and a form of appreciation for the valuable insight you have shared with us today.

For peer review only - http://bmjopen.bmj.com/site/about/guidelines.xhtml

Covid-19 Pandemic Concealing a Syndemic of Concern: Sex, Gender, Methamphetamine and Sexually Transmitted and Blood Borne Infections in People Living with HIV in Manitoba

I would like to start with some questions about how things generally are for you, but before we start are there any questions you want to ask?

1. **Could you please tell me about yourself?**
    a. PROBES
        i. How are things going for you right now?
        ii. How are you feeling coming in today?

2. **What does a typical week (day) look like for you?**
    a. PROBES
        i. Daily routine
        ii. Work/volunteer
        iii. Medical programs/services
        iv. Time with friends/family
        v. Time with caring for friends/family

3. **How are things going with your health right now?**
    a. PROBES- ask about:
        i. Physical health
        ii. Mental health
        iii. Emotional health
        iv. Spiritual health
        v. Financial health

Now I would like for us to talk about the HIV care and other health services that you receive.

4. **What was your experience when you were first diagnosed with HIV?**
    a. PROBES
        i. Any available support or counselling?
        ii. Were you given enough information about your options? (i.e. explained about your rights, how to access to care, follow up, linkage to doctor, treatment options?)
        iii. Did you learn about how HIV is transmitted? And how to prevent transmission?
        iv. Did you receive information about importance of treatment?

For peer review only - http://bmjopen.bmj.com/site/about/guidelines.xhtml

Covid-19 Pandemic Concealing a Syndemic of Concern: Sex, Gender, Methamphetamine and Sexually Transmitted and Blood Borne Infections in People Living with HIV in Manitoba

5. **What health services do you currently receive for HIV health care?**
    a. PROBES,
        i. Attend clinic appointments, see a NP, take medication, regular blood testing, etc. (Linked to Care)
        ii. Alternative medicine such as traditional or cultural medicine
        iii. Anything else?
    b. **If not linked to care move to question 7**

6. **What do you like about the HIV health care services you receive?**
    a. PROBES
        i. Relationship with doctor/ nurse/ social worker
        ii. Accessibility of location to receive services
        iii. Welcoming environment at service provider location
        iv. Any other reason?

7. **What problems have you encountered when accessing HIV care?**
    a. PROBES
        I. Stigma, discrimination from service providers
            i. Drug use, unhoused, gender identity
            ii. If yes, please tell me more about that. Where does this discrimination occur?
        II. Transportation, accessibility challenges
        III. Lack of childcare to get to attend appointments or children are not able to accompany to appointment
        IV. Income, lack of money to pay for medication
        V. Other challenges are more important to deal with
        VI. Lack of access to appointments, lengthy wait times to receive care, etc.
        VII. Mental health challenges
        VIII. Any other things you don't like?

8. **Could you please walk me through a time when you needed HIV care but you could not get it?**
    a. PROBES
        i. Ask participant to share a specific experience related to a barrier
        ii. If you were diagnosed before the pandemic (2018-2019), did you experience similar or different challenges before or during the COVID-19 pandemic (2020-current)?

For peer review only - http://bmjopen.bmj.com/site/about/guidelines.xhtml

Covid-19 Pandemic Concealing a Syndemic of Concern: Sex, Gender, Methamphetamine and Sexually Transmitted and Blood Borne Infections in People Living with HIV in Manitoba

9. **Has the COVID-19 pandemic impacted the HIV health services you use? If yes, in what ways?**
   a. PROBES
      i. Availability of appointments, shortage of staff at clinics, services not accessible, staff changes, etc.
      ii. Increased fear/risk due to COVID-19 in health care settings
      iii. Transportation issues
      iv. Money, financial challenges
      v. Use of substances
      vi. Mental health challenges/and or lack of support for mental health

10. **How safe do you feel in or around health care settings?**
    a. PROBES
       i. In the neighborhood around the clinic/hospital
       ii. Around security personnel such as police, hospital security guards
       iii. Fear of Child & Family Services, removal of children, or fear of other government agencies/actors
       iv. Lack of privacy to discuss health status (i.e., ER visit)
       v. Not safe due to perceived gender identity, race/ethnicity
       vi. Getting to your appointment- location of clinic, transportation

11. **What are things that help you to get the health care you need?**
    a. PROBES
       i. Positive relationship with doctor/health professionals
       ii. Welcoming and supportive environment at health centres, health centres specific to my needs (i.e. gender specific, LGBTQ+)
       iii. Availability/hours
       iv. Location within the city
       v. Support (personal or peer) to attend health appointments

12. **What changes do you think would make it easier for you (and other people living with HIV) to access care?**
    a. PROBES
       i. Transportation support, financial support
       ii. Peer support (i.e., someone to accompany you to appointments)
       iii. Better accessibility (e.g., different sites for treatments, availability of appointments, options for care)
       iv. Access to counselling and/or mental health support
       v. Addressing stigma
       vi. Different medication with less side effects
       vii. Access to material support (housing, food, etc.)
       viii. Bundling more services in one appointment- seeing multiple professional, etc. primary care physician, gynaecologist, mental health professional,

For peer review only - http://bmjopen.bmj.com/site/about/guidelines.xhtml

Covid-19 Pandemic Concealing a Syndemic of Concern: Sex, Gender, Methamphetamine and Sexually Transmitted and Blood Borne Infections in People Living with HIV in Manitoba

ix. Addressing multiple health concerns at each appointment- either related to HIV or other health issue

I have a few more questions before we end the interview, they are related to substances you may use. These questions may not apply to you, and we want to assure you that there is no judgment based on your use of substances. These questions will help us find ways to better support people who are living with HIV who also may use substances. I also want to remind you that you do not have to answer any questions you do not feel comfortable with, and that all of your answers are confidential.

13. Do you have any experience with using substance?
IF YES, ask the following questions. If no, move to QUESTION 18

14. How old were you when you started using substances?
- a. PROBE:
  - i. What substance(s) did you start with?

15. How would you describe your substance use when you started?
- a. PROBES:
  - i. Heavy use (one or more times per day)
  - ii. Moderate use (few times per week)
  - iii. Light use (once a week or a few times a month)
  - iv. Very light (less than once a month)

16. What type of substances do you currently use?
- a. PROBES
  - i. If crystal meth- do you inject or smoke or both?
  - ii. If opioids- inject or smoke, or pill?
- b. **If YES to injection ask question 18, if NO skip question 18**

17. How would you describe your current substance use?
- a. PROBES
  - i. Several times per day (one or more)
  - ii. Few times a week
  - iii. Once a week or a few times a month
  - i. Less than once a month

18. How do you access the substances you use?
- a. PROBE
  - i. Do you get it yourself or does someone else get them for you?)

This next series of questions we are interested in better understanding the practices you use to inject drugs.

For peer review only - http://bmjopen.bmj.com/site/about/guidelines.xhtml

Covid-19 Pandemic Concealing a Syndemic of Concern: Sex, Gender, Methamphetamine and Sexually Transmitted and Blood Borne Infections in People Living with HIV in Manitoba

19. If you use substances that you inject, how often do you:

a. **Use a new needle, spoon, sterile water or filter?**
   i. All of the time, some of the time, rarely or never?
   ii. If only some of the time or never- what are the barriers to accessing safe injection supplies?

b. **Inject with a needle or syringe used by somebody else?**
   i. All of the time, some of the time, rarely, or never?

c. **Clean and disinfect the injection site?**
   i. All of the time, some of the time, rarely, or never?

d. **Prepare using a spoon, water, or filter used by somebody else?**
   i. All of the time, some of the time, rarely, or never?

e. **Have a safe place to use?**
   i. All of the time, some of the time, rarely, or never?
   ii. If yes, what makes this place safe?
   iii. If no, what makes this place unsafe?

20. Do you know where to find harm reductions supplies?

a. PROBE
   i. If yes, where do you get them from?
      1. What supplies do you get?
      2. How often do you get harm reduction supplies?

21. Have you experienced any violence related to your substance use?

a. PROBE
   i. If yes, and you feel comfortable, can you please tell me what happened?

22. What would make using substances safer for you?

a. PROBE
   i. Better access to harm reduction supplies
   ii. Safe injection site
   iii. Decriminalization of drug use
   iv. Safe person to use drugs with

23. Does using substances affect your ability to access HIV care & services?

a. PROBES
   i. If yes, in what ways?

For peer review only - http://bmjopen.bmj.com/site/about/guidelines.xhtml

Covid-19 Pandemic Concealing a Syndemic of Concern: Sex, Gender, Methamphetamine and Sexually Transmitted and Blood Borne Infections in People Living with HIV in Manitoba

24. How did the COVID-19 pandemic impact your substance use?

a. PROBE

i. What changes in your pattern of use- availability, safety of substance, harm reduction supplies?

ii. Changes to type of substance used or ways of using- did you use more of one substance or start injecting instead of smoking?

25. What do you think is the best way [how do you want] to learn about prevention and treatment of STBBI's and harm reduction practices?

26. Is there anything else you want to tell us today?

That is the end of the interview portion of our time together today.

Thank you very much for speaking with me. I really appreciate you taking the time today to do this. I know some of these questions were very personal and might have brought up some emotions for you. If you need support after our chat, I can provide you a list of resources ***[give participant mental health support resource document].*** *[Insert name of counsellor or Elder] is available to speak with you now if you would like that support. You can also speak to [Participating Organization] and they can direct you to any other supports you can access.*

Now that we have completed the interview portion of the interview, we will take a short break ***[discuss with the participant what they need to do- offer Elder support, food, break to walk around, smudge ceremony, stretch/move their body, etc.].*** *When we come back together, I will ask you to please fill out three short surveys. This should take another 30-45 minutes to complete. After that if there are things, you want to add to this discussion, we will make sure you have enough time to do that.*

For peer review only

For peer review only - http://bmjopen.bmj.com/site/about/guidelines.xhtml

<u>Participant Survey: Demographics & Life Circumstances</u>

The following short questionnaire asks questions about you, such as your age, identity, where you live, your experiences with substance use, your sexual practices, and other life circumstances you may have encountered. This information is very helpful as it provides a better understanding of your experiences as a person living with HIV. We understand that these questions are very personal and intimate, and we appreciate your responses. **There is no judgement about anything you share with us**. Every person has different life experiences and situations that affect their health and well-being. Some of these questions may not apply to you, **please only answer what is relevant to your life**.

Please **<u>circle the answer(s) that applies to you, or write your answer in the space provided</u>. If there are questions you do not feel comfortable answering, please select "Prefer not to say".** Please ask your interviewer if you would like them to better explain any of the questions.

Thank you for taking part in our survey!

PART A: Demographics

1. What is your date of birth?   Day: ______/Month: ______/Year: _______

2. How old are you?

   ____

3. What gender identity best describes you?
   a. Woman
   b. Man
   c. Transgender woman
   d. Transgender man
   e. Non-binary (agender, genderfluid, pangender, genderqueer, gender nonconforming, etc.)
   f. Two-Spirit
   g. Other
      Which? ______________________
   h. Prefer not to say

For peer review only
For peer review only - http://bmjopen.bmj.com/site/about/guidelines.xhtml

4. What sex were you assigned at birth? (as shown on your original birth certificate)
   a. Female
   b. Male
   c. Intersex
   d. Prefer not to say

5. What sexual orientation best describes you?
(Please select all that apply to you)

   a. Lesbian
   b. Gay
   c. Bisexual
   d. Asexual
   e. Heterosexual/Straight
   f. Pansexual
   g. Other
      Which?____________________

Prefer not to say

6. What cultural background best describes you?
(Please select all that apply to you)
   a. Indigenous
      i. First Nations
      ii. Métis
      iii. Inuk
      iv. Other ____________________
      v. Unspecified ____________________
   b. African
   c. White/European
   d. East/Southeast Asian
   e. South Asian
   f. South East Asian
   g. Hispanic or Latinx
   h. Middle Eastern
   i. Other (please specify) ______________________
   j. Prefer not to say

For peer review only - http://bmjopen.bmj.com/site/about/guidelines.xhtml

7. What is your marital status?

a. Single
b. Married
c. Divorced
d. Common-law
e. Widowed
f. Other
Which________________________________
g. Prefer not to say

8. What languages do you speak? **(Please select all that apply to you)**

Languages	Yes	No	Which (please specify) *
English			
French			
Cree			
Dakota			
Dene			
Inuktitut			
Michif			
Ojibway			
Oji-Cree			
Filipino			
Spanish			
German			
Ukrainian			
Punjabi			
Other (please specify)			
Prefer not to say			

*It applies just for “Other” option answers

For peer review only - http://bmjopen.bmj.com/site/about/guidelines.xhtml

9. What religion best describes you? **(Please select all that apply to you)**

a. Traditional Indigenous
b. Christianity
   i. Catholicism
   ii. Witness of Jehovah
   iii. Evangelical
   iv. Protestant
   v. Orthodox
c. Buddhism
d. Hindu
e. Jewish
f. Muslim
g. Sikh
h. Other Religion________________________
i. No religious affiliation
j. Prefer not to say

10. What is the highest level of education you have completed?

a. K-12
   Which was the last grade? ______________
b. Certificate, diploma, vocational course from an educational institution
c. Bachelor's degree
d. Master's degree
e. Doctorate
f. Other, which _________________________
g. Prefer not to say

11. Where do you reside? (City/Town/Rural Community/Reserve)

a. _____________________________________

For peer review only

For peer review only - http://bmjopen.bmj.com/site/about/guidelines.xhtml

12. The following question are about **difficulties you may have doing certain activities**. Please tell us only about problems that **have lasted or are expected to last for six months or more**. Mark your response with an **X** for each difficulty.

Difficulties	No	Sometimes	Often	Always	Prefer not to say
Seeing					
Hearing					
Walking, using stairs, using hands or finger or doing other physical activities					
Learning, remembering, or concentrating					
Any emotional, psychological, or mental health conditions? (Anxiety, bipolar disorder, substance use, anorexia, etc)					

PART B. Income and Housing

Now we will ask you some questions related to your income and housing. These answers will allow us to better understand the resources that people living with HIV may need to support their health and well-being.

13. Are you currently employed?

a. Yes
b. No
c. Prefer not to say

14. What is your main source or source(s) of income?

____

15. Which of the following is your approximate yearly income?

a. less than 10,000 CAD/year
b. 10,000 – 19,999CAD/year
c. 20,000 - 29,999 CAD/year
d. 30,000 - 39,999 CAD/year
e. 40,000 - 49,999 CAD/year
f. More than 50,000/year
g. Prefer not to say

For peer review only - http://bmjopen.bmj.com/site/about/guidelines.xhtml

16. **Please** mark with an **X** any items that you currently **have** **or** **do not** have enough income to cover. **(Please select all that apply to you)**

Items	Have Enough	Do not have Enough	Prefer not to say	Does not apply
Housing (rent, hydro, water)				
Transportation				
Food				
Clothing				
Childcare				
Pet Care				
Healthcare				
Other (please specify)_______________				

17. What is your current living situation? **(Please select all that apply to you)**

Living situation	Yes	No	Which (please specify) *
Living alone (house/apartment)			
Living with partner (married, common-law or relationship)			
Living with children			
Living with roommates			
Living with extended family members			
Experiencing housing instability (insecure housing, shelter, transitional housing, houseless)			
Other (please specify)			
Prefer not to say			

*It applies just for "Other" option answers

Questions 17, 18, and 19 only apply to people who are experiencing housing instability.

18. How long have you experienced housing instability?

________ ____________ ___________

Days Months Years

For peer review only - http://bmjopen.bmj.com/site/about/guidelines.xhtml

19. If you are experiencing housing instability, which of the following are you experiencing? **(Please select all that apply to you)**

Living situation	Yes	No	Which (please specify) *
Insecure housing (month to month, threats by landlord to leave, unsure how you will pay rent next month)			
Couch surfing			
Sleeping rough (sleeping in the streets)			
Staying at a shelter			
Short-term housing program			
24/7 space			
Other (please specify)			
Prefer not to say			

*It applies just for "Other" option answers

20. If you are experiencing housing instability, did you experience housing instability before or after being diagnosed with HIV?

a. Before I was diagnosed
b. After I was diagnosed
c. Before and after I was diagnosed
d. Other
   Which?____________________
e. Prefer not to say

PART C: Criminal Justice System

The next few questions we want to ask you are related to your experiences with the criminal justice system. We are asking these questions as recent HIV infections have shown a number of infections in people who were incarcerated. These questions will help us to also understand how policing and surveillance may be affecting your life and health. We do not judge any of your answers and we are not affiliated in any way with police or the justice system.

For peer review only - http://bmjopen.bmj.com/site/about/guidelines.xhtml

21. Have you ever been incarcerated? (prison, jail, correctional facility)

	Initial date (from)	Final date (to)
Yes	Month: ________/Year: ________	Month: ________/Year:________
	Month: ________/Year: ________	Month: ________/Year:________
	Month: ________/Year: ________	Month: ________/Year:________
No	(Skip to Part D)	
Prefer not to say		

For peer review only

22. Did you receive an HIV positive diagnosis while incarcerated?
   a. Yes
   b. No **(Skip to question 23)**
   c. Prefer not to say

23. **If you did receive a positive diagnosis while incarcerated**, who told you about your diagnosis? **(Please select all that apply to you)**
   a. Physician
   b. Nurse
   c. Administration staff
   d. Prison Guard
   e. Other____________________
   f. Prefer not to say

24. Did you experience discrimination while incarcerated because of your HIV diagnosis?
   a. Yes
   b. No **(Skip to question 25)**
   c. Prefer not to say

For peer review only - http://bmjopen.bmj.com/site/about/guidelines.xhtml

25. If you did experience discrimination because of your HIV diagnosis, who did you experience discrimination from? **(Please select all that apply to you)**

	Yes	No	Who (please specify) *
Other incarcerated people			
Prison guards			
Healthcare staff			
Administration staff			
Child and Family Services (CFS)			
Other (please specify)			
Prefer not to say			

*It applies just for "Other" option answers

26. Have your experiences with the criminal justice system affected your ability to access HIV care and other health care services after you were released?

a. Yes
b. No
c. Prefer not to say

27. Please select with an **X** the services you had access to while incarcerated

Services	Had Access	Did not have access
Primary health care services (regular physicals, doctor appointments)		
HIV related care (medication, regular blood testing, specialist appointments)		
STBBI testing		
Harm Reduction Supplies (condoms, syringes, pipes)		
Child and Family Services (CFS)		
Mental health services and counselling		
Ceremony		

*Sexually Transmitted and Blood Borne Infections

For peer review only - http://bmjopen.bmj.com/site/about/guidelines.xhtml

28. Are you currently under correctional supervision (e.g., bail conditions, probation, or parole)?

	Start date (from)	Final date (to)
Yes	Month: ________/Year: ________	Month: ________/Year:________
No	**(Skip to Part D)**	
Prefer not to say		

PART D: Sexual Practices

Now we would like to ask you some questions about your sexual practices. Please answer what you are comfortable with. We are asking these questions as people can acquire HIV through sexual activity but that is not the only way. The reason we are asking these questions is to help us better understand the sexual practices of people living with HIV which can be useful when informing safe sex education.

29. Are you currently sexually active?

a. Yes
b. No
c. Prefer not to say

30. In the 12 months have you had sex with **(Please select all that apply to you)**

	Yes	No	Which (please specify) *
Women			
Men and women			
Men			
Non-binary person			
Two-spirit person			
Other (please specify)			
Prefer not to say			

*It applies just for "Other" option answers

31. In the past 12 months how many sexual partners have you had?

a. ________________
b. Prefer not to say

For peer review only - http://bmjopen.bmj.com/site/about/guidelines.xhtml

32. When engaging in sexual activity, do you use any of the following safe sex protections?

Protections	All of the time	Some of the time	Never
Condoms			
Dental dams			
Pre-exposure prophylaxis (Prep)			
Post exposure prophylaxis (Pep)			
Regular STBBI testing			
Other (please specify)________________			

33. How easy was it to get safe-sex protections before and during the COVID-19 pandemic?
a. Easier before COVID-19 pandemic
b. No difference before and during COVID-19 pandemic
c. Easier during COVID-19 pandemic
d. Prefer not to say

34. Do you use a form of contraception? (Birth control, condoms)
a. Yes
b. No **(Skip to questions 36)**
c. Prefer not to say

35. Mark with an **X** your use of the following forms of contraception:

Contraception	Yes	No	Which (please specify)
Short-acting hormonal methods (oral birth control)			
Long-acting reversable contraceptive (IUD)			
Barrier Methods (condom, sponge, cervical cap)			
Natural rhythm methods			
Sterilization (tubal ligation or vasectomy)			
Other (please specify)			
Prefer not to say			

For peer review only - http://bmjopen.bmj.com/site/about/guidelines.xhtml

36. How often do you get blood tests for sexually transmitted infections and blood born infections (Hep C, Syphilis, Gonorrhea)?
   a. Before engaging in sexual activity with a new partner
   b. Once a month
   c. Every few months
   d. Once a year
   e. When my doctor/nurse practitioner recommends
   f. Never
   g. Other
      When (please specify)?________________________________
   h. Prefer not to say

PART E: Substance Use

We would now like to ask you about your substance use. These questions may or may not apply to you, and we want to assure you that there is no judgment based on your use of substances. We are asking these questions to understand how to better support people who are living with HIV who also may use substances

37. Do you currently use substances? (alcohol, marijuana, crystal meth, crack, cocaine)
   a. Yes
   b. No
   c. Prefer not to say

38. Please fill in this chart. For every substance please select with an (x) the quantity and length of consumption

Substance	Consumption			Quantity Consumed					Time of consumption			
	Never	Past	Present	Quantity	Daily	Weekly	Monthly	Yearly	Length	Days	Months	Years
Tobacco				# of cigarettes								
Alcohol				# of drinks								
Inhaled Substances				# of inhalations								
Smoked Substances				# of times smoking								

For peer review only - http://bmjopen.bmj.com/site/about/guidelines.xhtml

Injected Substances				# of injections								

39. How old were you when you started using substances?

____
Age

40. What substance did you start with?

____
Name(s) of substance

41. Please describe your current and past substance use by using an **X** in every line

	Heavy use (one or more times per day)	Moderate use (few times per week)	Light use	Very light use (once a week or a few times a month)	Not using
Current substance use					
Substance use when you started					

42. Does substance use impact your sexual practices in any of the following ways, select with an **X** if yes or no:

**Impacts**	**Yes**	**No**	**Prefer not to say**
I am **more likely** to engage in sex with multiple partners when using substances			
I am **less likely** to engage in sex with multiple partners when using substances			
I am **less likely** to use protection when engaging in sexual activity when using substances			
I am **more likely** to use protection when engaging in sexual activity when using substances			
Other, which			

For peer review only - http://bmjopen.bmj.com/site/about/guidelines.xhtml

43. If you use substances that you inject, **how often do you**:

Practices	All of the time	Some of the time	Not very often	Never
Use a new needle				
inject with a needle or syringe used by somebody else				
Bleach or clean needles used by somebody else				
Prepare using a spoon, water, or filter used by somebody else				
Have someone else inject your substances				
Have a safe place to inject				
Know where to find harm reduction supplies				

PART F: Experiences with Violence

For the last portion of this survey, we want to better understand your experiences with violence. This section may or may not apply to you. The reason we are asking these questions is because during the COVID-19 pandemic there was a reported increase in experiences of violence, in particular intimate partner violence and domestic violence. There is also a higher rate of women who have been diagnosed with HIV in Manitoba over the past few years – and women, transgender, non-binary people, as well as those who identify as Indigenous and 2SLGBTQQIA+ are more likely to experience all forms of violence.

For peer review only - http://bmjopen.bmj.com/site/about/guidelines.xhtml

44. Throughout your life, have you ever experienced any of the following types of violence? **(Please select all that apply to you)**

Types of Violence	Any point in your life	Past 3 years	Currently
Physical violence			
Sexual violence			
Stalking, harassment			
Emotional abuse			
Intimate Partner Violence (violence perpetrated by a partner in a romantic or dating relationship)			
Domestic Violence (violence among people in a domestic situation- can include any type of family, or roommate)			
Cyberstalking/cyberbullying			
Child abuse			
Psychological / mental violence			
Other, which			
Prefer not to say			

45. If you are currently experiencing Intimate Partner Violence, mark with an **X** the ways it impacts you:

**Impacts**	**Yes**	**No**	**Prefer not to say**
I am not able to attend HIV clinic appointments or HIV related care because of my partner (Partner controls my time and schedule, money for health care costs, etc.)			
I am afraid of my partner finding out about when I am going to doctor appointments or taking medication			
My health care is not impacted, and I am able to attend all my appointments			
Other, which			

46. Have you received any support or health care services to support you with healing from experiences of violence (trauma counselling, psychological support, traditional healing)?

a. Yes
b. No **(Skip to question 48)**
c. Prefer not to say

For peer review only - http://bmjopen.bmj.com/site/about/guidelines.xhtml

47. If you have received support following your experiences with violence, which of the following have you received? **(Please select all that apply to you)**

Supports	Yes	No	Which (please specify) *
Counselling (Trauma counselling, Domestic Violence counselling)			
Psychological Support			
Support groups			
Traditional healing			
Domestic Violence shelter			
Women's centre			
Other (please specify)			
Prefer not to say			

*It applies just for "Other" option answers

48. Has anyone shared your HIV status without your consent?

a. Yes
b. No
c. Prefer not to say

That brings us to the end of the survey. Thank you again for taking the time to fill out this survey. We really appreciate your time and willingness to share your experiences with us. If you have any questions or would like to talk more with the interviewer, or support person, that can happen now. We have also created a resource sheet to access support or counselling if you would like a copy.

For peer review only - http://bmjopen.bmj.com/site/about/guidelines.xhtml

Service Provider Interview Question Guide

To be completed by facilitator	***Facilitator Name***		
Participant ID:			
Participating Organization:			
Location:			
Interview date:			
Interview time:	Start	End	Duration
Confirmation of consent signed:			

Thank you for agreeing to speak with me today. My name is [INSERT] and my pronouns are INSERT]. My role with this project is as a [Research Assistant, Research Associate].

Before we start the formal interview questions, I would like to remind you that we are here to speak about your experiences as a service provider [clinic program providers and/or social service employee] to provide additional perspectives on institutions, structures and policies related to HIV Care in Manitoba. The interview should take approximately 45 to 60 minutes.

Everything you share with me will be kept confidential. I also want to remind you that that our conversation will be recorded so your words are captured as accurately as possible, but the recording will be kept securely and destroyed once the study is over.

You have been asked to participate in this interview as you:

1) *Are a clinic service provider or social service employee who works with and/or provides services to people living with HIV or other STBBI's in Manitoba [and/or people who inject drugs]*

Does this statement apply to you?
Yes
No

For peer review only - http://bmjopen.bmj.com/site/about/guidelines.xhtml

[If no, relay that they are not eligible to participate in the study. Thank them for their time, and end interview].

For the purpose of our discussion, STBBI Services may include:

- *HIV testing / care*
- *Hepatitis C testing / care*
- *Syphilis testing / care*
- *Other sexually transmitted infection (STI) testing /care*
- *Obtaining safe sex products (condoms, dental dams, pre/post exposure prophylaxis*
- *Information about STBBI testing/care*
- *Counselling related to STBBIs*
- *Community services or supports related to STBBIs*

Do you have any questions for me before we begin?

1. **Could you please describe your role?**
    a. PROBES
        i. Type of organization (Clinic, CHC, Community Organization)
        ii. Position Title
        iii. Main job duties
        iv. Schedule

2. **What services does your organization provide?**
    a. PROBES
        i. Women's health, mental health, addictions?

3. **Please tell me about what services are working well for your clients/patients living with HIV?**
    a. PROBES
        i. Specific supports or programs
        ii. Accessibility
        iii. Campaigns
        iv. New services to deal with COVID-19

4. **What barriers to accessing or remaining in care have you identified for people living with HIV?**
    a. PROBES
        i. Attitudes to some clients
        ii. Limited hours or sites
        iii. Shortages of supplies or staff
        iv. Person's gender identity
        v. Stigma towards infectious diseases, substance use, race, etc.

For peer review only - http://bmjopen.bmj.com/site/about/guidelines.xhtml

5. **What barriers have you identified for PLHIV who are actively using substances that may prevent them from accessing care?**
    a. PROBES
        i. Attitudes of staff
        ii. Administrative regulations
        iii. Stigma associated with substance use, race, gender
        iv. Behaviour challenges related to substance use

6. **What major changes have you noticed in your clients/patients due to the COVID-19 pandemic?**
    a. PROBES
        i. Increased drug use
        ii. Increased experiences of violence
        iii. Mental health challenges
        iv. Housing instability
        v. Income instability
        vi. Incarceration
        vii. Increase in sex work
        viii. Suicidal ideation
        ix. Anything else?

7. **What are some examples of success factors that you have seen clients employ during the COVID-19 pandemic to ensure they continue to get the care they need?**

8. **How has the COVID-19 pandemic affected the services in your organization?**
    a. PROBES
        i. Changes of hours
        ii. Not enough staff to meet client needs
        iii. Virtual platform challenges
        iv. Increased needs of clients related to drug use
        v. Strain on health care system & referral services
        vi. Impact on staff to deliver services
            1. Burnout
            2. Getting sick themselves
            3. Not enough training for new staff

9. **How has the COVID-19 pandemic affected you personally as a service provider?**
    a. PROBES
        I. Mental health challenges
        II. Burnout
        III. Secondary trauma
        IV. Increased work load
        V. Covid-19 reinfections

For peer review only - http://bmjopen.bmj.com/site/about/guidelines.xhtml

10. **How do you support people whose needs fall outside of the services you offer?**
    a. PROBE
        i. Collaborate with other health providers or community agencies to connect people to these services
        ii. Relationships with other providers in the community

11. **What strategies do you currently use to support people living with HIV who use substances?**
    a. PROBES
        i. What additional training, resources, education do you need?

12. **What is your understanding of harm reduction and how do you apply this in your work?**
    a. PROBES
        i. Harm reduction- knowledge, attitudes
        ii. What else do you want to learn about harm reduction?

13. **What strategies do you use to support your clients with prevention of STBBI's?**
    a. PROBES:
        i. PrEP, STBBI testing, counselling/mental health support
        ii. Education/sharing knowledge about STBBI's- how they are transmitted, how are the treated?

14. **What do you personally do or what does your organization do to create a safe space/environment for clients?**
    a. PROBES
        i. Environment- accessibility, meeting clients where they are at, safety
        ii. Training- anti-racism, anti-oppression
        iii. Policies- sexual harassment, violence, covid safety, harm reduction
        iv. Practices- cultural safety, trauma informed care

15. **Can you tell me what you think about the policies in Manitoba related to HIV?**
    a. PROBES
        i. Specific policies working against ability to get, receive or remain engaged in care
        ii. Policies currently working well to support PLHIV
        iii. Policies you would create to support you in your work as a service provider

16. **What are your suggestions for policy change or program enhancements- that would both support PLHIV and service providers delivering care?**
    a. **PROBES**
        i. Additional options for care, additional services
        ii. Referrals to other services

For peer review only - http://bmjopen.bmj.com/site/about/guidelines.xhtml

iii. Bundling services in one appointment or location (wrap around care)

17. What additional resources are needed to better support people living with HIV in Manitoba?

a. **PROBES**
   i. Prevention- i.e. PREP
   ii. Additional options for care
   iii. Knowledge about STBBI's- how they are transmitted, how are the treated?

18. What do you think is the best way [how do you want] to learn about prevention and treatment of STBBI's and harm reduction practices?

19. We are asking everyone involved in this study to share their gender. If you are comfortable, can you please tell us how you identify?

That is the end of our interview today. *Before we go, I wanted to know if you wanted to share anything else with me? Or if you think we should have asked you about something else?*

Thank you very much for speaking with me. *We really appreciate your time and efforts and the valuable information you have shared with us today.*

For peer review only - http://bmjopen.bmj.com/site/about/guidelines.xhtml

BMJ Open

Social and structural barriers and facilitators to HIV health care and harm reduction services for people experiencing syndemics in Manitoba: Study protocol

Journal:	*BMJ Open*
Manuscript ID	bmjopen-2022-067813.R2
Article Type:	Protocol
Date Submitted by the Author:	08-Jun-2023
Complete List of Authors:	Rueda, Zulma; University of Manitoba Faculty of Health Sciences, Department of Medical Microbiology and Infectious Diseases<br>Haworth-Brockman, Margaret; National Collaborating Centre for Infectious Diseases; University of Manitoba, Department of Community Health Sciences<br>Sobie, Cheryl; University of Manitoba Faculty of Health Sciences, Department of Medical Microbiology and Infectious Diseases<br>Villacis, Enrique; University of Manitoba Faculty of Health Sciences, Department of Medical Microbiology and Infectious Diseases<br>Larcombe, Linda; University of Manitoba Faculty of Health Sciences, Department of Internal Medicine<br>Maier, Katharina; The University of Winnipeg, Criminal Justice<br>Deering, Kathleen; The University of British Columbia, Department of Medicine; Centre for Gender & Sexual Health Equity<br>Sanguins, Julianne; Manitoba Metis Federation; University of Manitoba, Department of Community Health Sciences<br>Templeton, Kimberly; Manitoba HIV Program; Nine Circles Community Health Centre<br>MacKenzie, Lauren; Manitoba HIV Program; University of Manitoba Faculty of Health Sciences, Department of Internal Medicine<br>Ireland, Laurie; Nine Circles Community Health Centre; Manitoba HIV Program<br>Kasper, Ken; University of Manitoba, Internal Medicine; Manitoba HIV Program<br>Payne, Michael; Nine Circles Community Health Centre<br>Bullard, Jared; University of Manitoba, Medical Microbiology and Infectious Diseases; Cadham Provincial Laboratory<br>Krusi, Andrea; The University of British Columbia, Department of Medicine; Centre for Gender & Sexual Health Equity<br>Pick, Neora; Oak Tree Clinic; The University of British Columbia, Division of Infectious Disease<br>Myran, Tara; University of Winnipeg, Indigenous Development<br>Meyers, Adrienne; Indigenous Services Canada, Laboratory Integration, Office of Population and Public Health<br>Keynan, Yoav; University of Manitoba Faculty of Health Sciences, Internal Medicine; National Collaborating Centre for Infectious Diseases
**Primary Subject Heading**:	HIV/AIDS

Secondary Subject Heading:	Epidemiology, Qualitative research
Keywords:	COVID-19, HIV & AIDS < INFECTIOUS DISEASES, QUALITATIVE RESEARCH, Sexual and Gender Minorities, Substance misuse < PSYCHIATRY, Health Services Accessibility

SCHOLARONE™
Manuscripts

BMJ Open Submission

Revised Manuscript – Clean version June 7, 2023

Title: Social and structural barriers and facilitators to HIV health care and harm reduction services for people experiencing syndemics in Manitoba: Study protocol

Authors:

Zulma Vanessa Rueda M.D., Ph.D.[1*ǂ], Margaret Haworth-Brockman M.Sc.[2,3ǂ], Cheryl Sobie M.A.[1], Enrique Villacis M.A.[1], Linda Larcombe Ph.D.[4], Katharina Maier Ph.D.[5], Kathleen Deering Ph.D.[6,7], Julianne Sanguins Ph.D.[3], Kimberly Templeton M.Sc.[9,10], Lauren MacKenzie M.D., MPH. [4,9], Laurie Ireland M.D.[9,10], Ken Kasper M.D.[4,9], Michael Payne B.A.[9,10], Jared Bullard M.D.[1,11], Andrea Krusi Ph.D.[6,7], Neora Pick M.D.[12,13], Tara Myran B.A.[14], Adrienne Meyers M.Sc. Ph.D.[15], Yoav Keynan M.D., P.h.D.[1,2,3,4,9ǂ]

[1] Department of Medical Microbiology and Infectious Diseases, University of Manitoba Rady Faculty of Health Sciences, Winnipeg, MB, R3E 0J9, Canada

[2] National Collaborating Centre for Infectious Diseases, Rady Faculty of Health Sciences, University of Manitoba, Winnipeg, MB, R3E 0T5, Canada

[3] Department of Community Health Sciences, University of Manitoba, Rady Faculty of Health Sciences, Winnipeg, MB, R3E 0J9, Canada

[4] Department of Internal Medicine, University of Manitoba Rady Faculty of Health Sciences, Winnipeg, MB, R3E 0J9, Canada

[5]Criminal Justice, The University of Winnipeg, Winnipeg, Canada

For peer review only

For peer review only - http://bmjopen.bmj.com/site/about/guidelines.xhtml

[6]Division of Social Medicine, Department of Medicine, Faculty of Medicine, The University of British Columbia, Vancouver, Canada

[7]Centre for Gender & Sexual Health Equity, Vancouver, Canada

[9]The Manitoba HIV Program, Winnipeg, Canada

[10] Nine Circles Community Health Centre, Winnipeg, Canada

[11] Cadham Provincial Laboratory, Winnipeg, Canada

[12] Oak Tree Clinic, BC Women's Hospital, Vancouver, Canada

[13] Division of Infectious Disease, University of British Columbia, Vancouver, Canada

[14] Indigenous Development, University of Winnipeg, Winnipeg, Canada

[15] Laboratory Integration, Office of Population and Public Health, Indigenous Service Canada, Winnipeg, Manitoba

[Ł] Principal investigators

***Correspondence**: Zulma Vanessa Rueda. Email: zulma.rueda@umanitoba.ca

For peer review only

Abstract

Introduction:

In Manitoba, Canada, there has been an increase in the number of people newly diagnosed with HIV and those not returning for regular HIV care. The Covid-19 pandemic resulted in increased sex and gender disparities in disease risk and mortalities, decreased harm reduction services and reduced access to health care. These health crises intersect with increased drug use and drug poisoning deaths, houselessness, and other structural and social factors most acutely among historically underserved groups. We aim to explore the social and structural barriers and facilitators to HIV care and harm reduction services experienced by people living with HIV (PLHIV) in Manitoba.

Methods and analysis:

Our study draws upon participatory action research (PAR) design. Guiding the methodological design are the lived experiences of PLHIV. In-depth semi-structured face-to-face interviews and quantitative questionnaires will be conducted with two groups: (1) Persons ≥18 years old living or newly diagnosed with HIV, and (2) service providers who work with PLHIV. Data collection will include sex, gender, sociodemographic information, income and housing, experiences with the criminal justice system, sexual practices, substance use practices and harm reduction access, experiences with violence and support, HIV care journey (since diagnosis until present), childhood trauma and a decision-making questionnaire. Data will be analyzed intersectionally, employing grounded theory for thematic analysis, sex- and gender-based analysis, and social determinants of health and syndemic framework to understand the experiences of PLHIV in Manitoba.

For peer review only - http://bmjopen.bmj.com/site/about/guidelines.xhtml

Ethics and dissemination:

We received approval from the University of Manitoba Health Ethics Research Board (HS25572;H2022:218), First Nations Health and Social Secretariat of Manitoba, Nine Circles Community Health Centre, Shared Health Manitoba (SH2022:194), and 7th Street Health Access Centre. Findings will be disseminated using community-focused knowledge translation strategies identified by participants, peers, community members, and organizations, and reported in conferences, peer-reviewed journals, and a website (www.alltogether4ideas.org).

Keywords: HIV care, HIV barriers and facilitators, syndemic, participatory action research, Covid-19, gender.

Strengths and Limitations

- A diverse research advisory and peer research team, and comprehensive data collection tools ensure we accurately capture people's insights into HIV care and harm reduction services.
- The intersectional lens would provide a better understanding of the current social and structural health inequities experienced by people living with HIV.
- The sample may not represent all circumstances since data collection will occur in two metropolitan areas and with individuals who can dedicate time to the study.

For peer review only - http://bmjopen.bmj.com/site/about/guidelines.xhtml

INTRODUCTION

Approximately 36.9 million people live with the human immunodeficiency virus (HIV) [1,2]. Despite global and local initiatives to address HIV [2–5], recent findings suggest a resurgence of diagnoses among North and South American and European countries [1]. In Canada, HIV diagnoses have steadily increased among women and people who inject drugs (PWID) [6,7]. In Manitoba, Canada, there is an overrepresentation of females, PWID, Indigenous peoples, and people experiencing houselessness among people newly diagnosed with HIV [8]. Heterosexual sex and injection drug use have become the most common mode of HIV transmission since 2018, and methamphetamine is the main injected drug [8]. In Manitoba, there is also an emerging pattern of people living with HIV (PLHIV) not returning for regular care and treatment. Manitoba also has not reached any of the UNAIDS' and Canada's 95-95-95 goal (i.e., 95% of all PLHIV know their status, 95% of those diagnosed receive antiretroviral treatment, and 95% of those on treatment achieve viral suppression) [9,10].

The care continuum for PLHIV encompasses several interconnected components (e.g., diagnosis, linkage and engagement to care) [11,12]. Linkage to care has been associated with positive health outcomes and reduced HIV mortality [12,13]. In a systematic qualitative review, Tso and colleagues [12] found that interventions focused on increasing community participation and literacy, creating community outreach mobile teams, integrating HIV-specific services in primary care, and providing substance use services improved linkage to care [12]. On the other hand, lack of information, anxiety, fear, and stigma after an HIV diagnosis were barriers to linkage to care [12]. Moreover, Hall and researchers [14] found that the leading reasons for people continuing HIV care were the use of community health workers and lay health workers, incorporating technology with health, support from friends and family, access to intensive case

For peer review only - http://bmjopen.bmj.com/site/about/guidelines.xhtml

management, and positive interpersonal relationships with service providers [14]. Personal and community stigma and discrimination, fear of HIV disclosure, and service provider shortages were among the leading barriers to PLHIV staying in care [14].

Previous findings have also highlighted how HIV experiences are shaped by biological sex and gender identities [15–18]. Ostrach and Singer [18] emphasize the importance of biological, social, and political factors that place women at an increased risk of acquiring HIV. The authors suggest integrating sex- and gender-based analyses in HIV research to accurately capture the impact of intersectional identities on PLHIV [18]. Argento et al. found that most women (i.e., cis and transwomen) living with HIV, who participate in sex work, and may or may not use methamphetamines had experienced physical or sexual abuse during childhood and adolescence, and those who initiated methamphetamines use were more likely to have experienced childhood abuse [15]. Their study emphasizes the need to consider childhood trauma in understanding health and social circumstances later in life [15]. The Canadian prairie provinces (Alberta, Saskatchewan, and Manitoba) report higher violence against women than the rest of Canada [19]. Findings in these provinces highlight the harm violence has on women's lives [20] and emphasize the importance of considering women's contexts and intersecting identities (e.g., cultural background, gender, disability) when developing person-centred interventions [20–23].

The Covid-19 pandemic has dramatically affected the health of many individuals, especially among people already burdened by social and structural health inequities [24]. The pandemic also exacerbated mental health symptoms in many groups [25]. Data from Manitoba shows that older adolescents and young adults self-reported increased stress/anxiety and depression, alcohol consumption and substance use, and conflict with family members and

For peer review only

For peer review only - http://bmjopen.bmj.com/site/about/guidelines.xhtml

intimate partner[26], with those self-identifying as women experiencing higher mental health burden [27,28], financial hardship, and interpersonal conflicts [26]. For HIV care, the reduction or complete closure of HIV treatment centres and services in some jurisdictions placed the cascade of care for PLHIV at risk of breaking down [24]. Among PWID, Covid-19 restrictions limited safe spaces for substance use, disrupted drug supplies, and restricted the availability of medical, community, and traditional resources [29].

Harm reduction encompasses strategies to reduce the health, social, and economic factors that harm PWID and their networks [30]. The Public Health Agency of Canada conducted an online survey among people self-identifying as PWID in the past six months to understand how sexually transmitted and blood borne infections (STBBIs) and harm reduction services changed during Covid-19 [31]. The survey reported 52% of respondents increased their personal use of methamphetamine, and more than half increased their use of alcohol and other drugs [31]. As well, 50% of PLHIV had challenges getting to a provider or clinic since the onset of Covid-19 [31]. More than half of the respondents had difficulty getting STBBI-related services, needle and syringe distribution programs, and naloxone training [31]. While these results highlight concerning trends, data were not reported by sex and gender, hindering an intersectional analysis. Also, the study's online modality may not have reached PWID who may also be coping with unstable housing, poverty, and other structural factors, likely underestimating the disruption of services. Locally, the province of Manitoba reported record fatalities from toxic drug overdoses [32], and PWID described harm reduction services limitations due to Covid-19 [29]. However, more research is needed to understand specific service limitations and how these limitations interact with other health crises and social inequities.

For peer review only - http://bmjopen.bmj.com/site/about/guidelines.xhtml

Taken together, these trends suggest that an intersectional lens is needed to understand how social, structural and programmatic factors affect HIV care and harm reduction services among PLHIV, including in the context of Covid-19 disruptions. This paper reports a study protocol that aims to address the question: What are the social and structural factors that women, men and non-binary people living with HIV in Manitoba are experiencing that prevent or enable HIV care and harm reduction services, before and during Covid-19 pandemic?

STUDY OBJECTIVES AND OUTCOMES

This study explores the social and structural factors that affect the access, linkage and retention in HIV health care and harm reduction services among women, men, and non-binary persons living with HIV (linked and non-linked to care), before and during Covid-19.

It is important to note that this study is one component of a more extensive mixed-methods project aimed at understanding the epidemiology of HIV and syndemics experienced by PLHIV in Manitoba.

Outcomes

1) To gain an in-depth understanding of the social and structural barriers and facilitators faced by women, men and non-binary persons living with HIV, affecting access, linkage and retention in HIV health care and access harm reduction services, before and during Covid-19 pandemic.
2) To understand how the Covid-19 pandemic has affected access, linkage and retention in HIV health care and harm reduction services for women, men and non-binary persons living with HIV.

For peer review only - http://bmjopen.bmj.com/site/about/guidelines.xhtml

METHODS AND ANALYSIS

Conceptual framework

The syndemic framework proposed by Singer [33] focused on understanding the interconnectedness of mutually reinforcing biomedical crises within their socioeconomic contexts. The syndemic theory "involves the adverse interaction between diseases and health conditions of all types (eg, infections [HIV and STBBI], chronic non-communicable diseases, mental health [issues], behavioural conditions, [substance use], toxic exposure, and malnutrition) and are most likely to emerge under conditions of health inequality caused by poverty, stigmatization, stress, [trauma], or structural violence" [33,34].

Imperative to syndemic theory is the recognition that disparate social conditions not only enable health crises but maintain and reinforce them [33]. Social and structural health inequities that disproportionately affect seldom-heard and marginalized populations in Manitoba require understanding of health as a social construct, rather than a biological process as defined by the World Health Organization Conceptual Framework for Action on the Social Determinants of Health (CSDH) [35]. The framework describes how social, economic, and political values and policies create a socioeconomic position, for people in societies, influencing opportunities for income, education, depending on race/ethnicity, gender, and other factors [35]. These structural determinants of health inequities interact with intermediary determinants of health such as material circumstances, and psychosocial, biological (including sex) and behavioural factors [35]. Using health as a social construct helps to push understanding of inequities beyond a focus on individual health and behaviors, framing health as a social construct dependent on the interplay of numerous interacting systems and circumstances.

For peer review only

For peer review only - http://bmjopen.bmj.com/site/about/guidelines.xhtml

This syndemic theory is particularly valuable in the context of communicable diseases and seldom-heard and marginalized peoples' health, as it brings interacting diseases and inequalities together, and it emphasizes the political, social, historical, and economic factors that have negatively affected their health outcomes [34,36]. Figure 1 summarizes our approach for this study.

Figure 1. Structural and social determinants [35] adapted to the main focus of our study.

A comprehensive framework that includes structural and social factors, is needed in Manitoba to understand the experiences of PLHIV that prevent and enable access, linkage and retention in HIV care and harm reduction services. Our methodology is intersectional by considering how sex and gender, cultural background, socioeconomic factors, (including housing), trauma and violence, substance use, empowerment and decision making, and other factors intersect [37] to affect individuals' experiences of HIV care and harm reduction services before and during Covid-19 pandemic.

Patient and Public Involvement

This study is grounded in principles of social justice, equity, and the understanding that PLHIV are experts whose voices should be central to the research design and process. Accurate findings are achievable, robust, and meaningful only if driven by people with lived experiences. Therefore, this study utilizes community engagement grounded in participatory action research (PAR; Figure 2).

For peer review only - http://bmjopen.bmj.com/site/about/guidelines.xhtml

Figure 2. Participatory Engagement Process

Legend: The participatory engagement process and how Participatory Action Research (PAR) will be utilized to achieve the outcomes related to this study [38–40]. *Research Objective: This study explores the social, structural and programmatic factors that affect the access, linkage and retention to HIV care and harm reduction services among women, men, and non-binary persons living with HIV (linked and non-linked to care), before and during Covid-19.

This project is a collaborative multi-disciplinary study connecting peers, community-based researchers, clinicians, and academic researchers across Canada. To ensure this project remains grounded in the lived experience of PLHIV and PWID, we partnered with several community-based organizations, convened a diverse Research Advisory Committee (RAC), and created a Peer Research Team (PRT) of people with lived experience.

Before the project commencement, the principal investigators held more than ten consultations with community members and peers, including PWID, PLHIV, and Indigenous peers impacted by the "60s scoop" (i.e., children were systematically removed from their homes and placed into the child welfare system; [41,42]). During these consultations, the community members' personal stories illustrated the complex intersections of sex, gender, stigma, substance use, colonial and structural violence, and other factors affecting care. These consultations provided insights into the interconnectedness of ongoing health crises and helped focus our research questions.

Several study co-investigators are the lead facilitators of HIV care and prevention in Manitoba, including The Manitoba HIV Program, which provides information, specialized care, and support to approximately 1500 PLHIV in Manitoba. Nine Circles Community Health Centre, The Manitoba Harm Reduction Network, Main Street Project, and Meeting the Moment are all

For peer review only - http://bmjopen.bmj.com/site/about/guidelines.xhtml

key organizations and initiatives supporting underserved groups and guiding this research process.

After initial consultations, we assembled a RAC (Supplementary material 1) which includes an Indigenous Elder and Indigenous leaders; community members with lived experience in substance use, houselessness, and intergenerational trauma; and academic and scientific researchers of HIV, STBBI, and sex and gender-based analyses. The RAC guides the research team in research design and community-based engagement methods, data collection tools, culturally safe approaches to recruitment and data collection, review of preliminary findings, and knowledge translation strategies. The RAC's continued involvement ensures that the project is culturally safe for the diverse communities involved, the community's best interests are considered, and the results and findings are shared appropriately with stakeholders.

Additionally, we convened a PRT of community members with lived experience (Supplementary material 2). The purpose of the PRT's is to involve community members as co-researchers during the design of the recruitment strategy and data collection tools, data collection, data analysis, findings, and knowledge translation. Our approach to peer engagement focuses on building reciprocal relationships and sharing skills with peer members for each step in the research process, respectfully and safely. PRT meetings are held 2-3 times per month for two and a half hours. Peers are paid for their time and provided with transportation, childcare support, and skill-building activities.

Indigenous peoples make up 18% of the population of Manitoba (compared to 4.9% of the total population in Canada) [43], yet are overrepresented in new HIV diagnoses in Manitoba [8]. While not all participants will self-identify as Indigenous, we have received guidance from peers, RAC members and experts in community-based research to ensure our project remains

For peer review only - http://bmjopen.bmj.com/site/about/guidelines.xhtml

culturally safe for all. Thus, a critical part of our research process centres around reciprocity. Reciprocity is a critical component of Indigenous culture that should be applied as an overarching stance rather than a particular interaction [44]. Meaningful collaboration with peers, sharing skills (e.g., workshops, training sessions) and co-learning are components of our commitment to reciprocity. The RAC emphasized the need for reciprocity during data collection (with PLHIV) suggesting connecting participants with care (if they consent), offering participants other supports (e.g., peer and cultural supports, trauma counselling), honorarium, transportation, and childcare support. Beyond the data collection, members of the PRT, community members and leaders of community-based organizations are working with the research team to share the preliminary and final findings, mobilize and advocate for improvements and changes, and to develop actionable strategies from the findings. These strategies may include training courses to build capacity on related subjects (e.g., prevention and treatment of STBBIS and HIV), community-based dissemination strategies, and advocacy strategies for increased resources. Likewise, an Indigenous Cultural Advisor joined the team to provide a culturally safe support to participants, peers, and the research team. Even though the Cultural Advisor will not be able to advise on indigenous cultural safety for each distinct Indigenous group, she has extensive experience working in community organizations and can provide referrals to culturally safe resources. The Cultural Advisor also educates the research team about the history of Indigenous People's in Canada, Indigenous values, traditions, ways of living, and culturally safe research approaches.

Participants and Settings

Inclusion criteria for PLHIV include (Group 1):

1. Resident of Manitoba

For peer review only - http://bmjopen.bmj.com/site/about/guidelines.xhtml

2. 18 years or older
3. Diagnosed with HIV

Inclusion criteria for service providers (Group 2):

1. Resident of Manitoba
2. Provide medical, social, or other services to PLHIV

The target sample size for this prospective study will be 15-25 PLHIV and 20-30 service providers who work with PLHIV.

Recruitment Process

Purposive sampling will be used due to the distinct eligibility requirements of the participant groups.

Group 1 Recruitment Process

Service providers from the Manitoba HIV Program locations (Health Sciences Centre HIV, Nine Circles Community Health Centre, and 7th Street Health Access) will be asked to support the recruitment of PLHIV. Service providers in these locations provide HIV-related support services and have established and trusted relationships with PLHIV. Recruitment materials will be shared with clinicians and service providers to discuss the study with potential participants. If participants are interested, they can contact the research team to discuss the study in more detail and confirm participation.

To support recruiting participants not linked to care, the research team will engage with community organizations supporting PLHIV who may use substances and may be experiencing unstable housing and mental health challenges. There has been a history of poor research relationships with PLHIV that have created experiences of mistreatment, exploitation, and

For peer review only - http://bmjopen.bmj.com/site/about/guidelines.xhtml

disconnect between researchers and communities [45]. Therefore, spending time in community locations potential participants frequent is critical to enable meetings, gain trust, and build rapport. Site observations at community organizations will create opportunities for connections and facilitate informal conversations with potential participants about the research. In line with the latest Manitoba HIV Program Report [8], the purposive sample in group 1 will ensure that at least half of the participants self-identify as women, followed by men and non-binary persons. Similarly, we will aim to include the experiences of Indigenous, PWID, and those with unstable housing experiences to accurately represent the people newly diagnosed in Manitoba [8].

Group 2 Recruitment Process

A list of organizations that support and provide services to PLHIV will be compiled to recruit service providers. The RAC and community partners will also be asked to suggest key informants. As with Group 1, a purposive sample will be used to identify a representative sample of participants who provide various services and identify as women, men, or non-binary. Likewise, the perspectives of Black, Indigenous, and People of Color (BIPOC) service providers will be considered for sampling.

Data Collection

We will collect quantitative and qualitative data focused on understanding HIV care and harm reduction services, social, structural and programmatic factors (Table 1).

Table 1. Data collection, sources, tools and variables collected in this study.

Source	Data type	Tool used	Variables
	Qualitative data	Interview Guide (supplementary material 3)	Personal description (Q1-3), experience during HIV diagnoses (Q4), services used for HIV care (Q5), barriers and facilitators to HIV care (Q6-8, Q11), impacts of Covid-19

For peer review only - http://bmjopen.bmj.com/site/about/guidelines.xhtml

People living with HIV			(Q9), safety in health care settings (Q10), changes to HIV care (Q12), substance use patterns, types of substances, and drug injection practices (Q13-19), harm reduction services (Q20, Q22), substance use and violence (Q21), substance use and HIV care (Q23), Covid-19 and substance use (Q24), best ways to learn about prevention and treatment of STBBI's and harm reduction practices (Q25).
	Quantitative data	Sociodemographic and life circumstances survey (supplementary material 4)	Age (Questions[Q] 1-2), sex at birth, gender identity, sexual orientation, cultural background, marital status, language, religion, education level, place of residence, disabilities, income and housing (Q13-19), experiences with the criminal justice system, including discrimination and stigma because of HIV diagnosis (Q21-28), sexual and testing practices (Q29-36), substance use patterns, quantity, types of substances, and drug injection practices (Q37-43), experiences with violence including supports received for healing, and involuntary disclosing of HIV status (Q44-48).
		Childhood Trauma Questionnaire[46]	Emotional abuse, physical abuse, sexual abuse, emotional neglect, and physical neglect.
		Empowr-[47]	Self-efficacy, perceived power, optimism about and control over the future, and community optimism
Service providers	Qualitative data	Interview Guide (supplementary material 5)	Sex and gender, role, organization, and services provided (Q1-3), facilitators and barriers to HIV care in PLHIV (Q3-4), barriers specifically for PLHIV who use substances (Q5), the impact of Covid-19 on clients, organization, and personal (Q6-9), types of supports provided for clients (Q10-11), understanding of harm reduction (Q12), prevention of sexually transmitted and blood borne infections (Q13, Q18), creating safe environments (Q14), HIV policies in Manitoba (Q15-17).

The research team will greet potential participants who contact the research team by email or phone. Research members will follow a phone or email script to discuss inclusion,

For peer review only - http://bmjopen.bmj.com/site/about/guidelines.xhtml

scheduling, accessibility, psychological, and emotional support for the data collection process. Psychological or emotional support available for this study will include mental health counselling, cultural counselling, and peer support. Resource(s) will only be arranged and offered upon request, to ensure participant confidentiality. Participants will be provided information and access to health care related to HIV, other STBBIs, counselling, or primary health through referrals to community organizations. All the field research teams have training in trauma-informed care, harm reduction, mental health first aid, and non-violent crisis intervention. This safety process ensures that participants can receive support, as some of the themes explored may elicit emotional responses.

For PLHIV, data will be collected by the research associate using a combination of face-to-face in-depth semi-structured qualitative interviews and three surveys. The qualitative component will use an interview guide with 26 open-ended questions (Supplementary Material 3). The interview guide used for this study has been revised several times by the RAC, the PRT, and key stakeholders (e.g., service providers). Questions focus on experiences with acquiring and receiving an HIV diagnosis, connecting and receiving HIV care, discrimination and stigma, substance use, past trauma and violence, changes in life due to Covid-19, and knowledge and experiences with harm reduction services (Table 1). In addition, PLHIV will be invited to suggest changes to HIV care and broader health and social care in Manitoba. During the screening process, participants will be offered a PRT member as a co-interviewer and a cultural advisor accompanying the research associate. PRT members will be trained to conduct interviews and facilitate surveys. Many PRT members already have experience in community-based research, so they will be familiar with the data collection tools. At the suggestion of the RAC, we will schedule a break after the interviews to offer various activities (e.g., meditation

For peer review only - http://bmjopen.bmj.com/site/about/guidelines.xhtml

and stretching exercises) and food (e.g., granola bars and juice boxes) that create a welcoming and comfortable environment for participants.

Three surveys will be conducted after the break, including a Participant Demographic and Life Circumstances Survey (Supplementary Material 4), Childhood Trauma Questionnaire (Short-Form) [46], and the Empowr- Making Decisions Survey [47]. The research team developed the Participant Demographics and Life Circumstances survey in consultation with key stakeholders and people with lived/living experiences. It enquires about PROGRESS (place of residence, race/ethnicity/culture/language, occupation, gender/sex, religion, education, socioeconomic status, and social capital) factors. Employing PROGRESS indicators provides an equity lens in highlighting health disparities [48]. These questions will allow describing the structural factors influencing participants' health. This survey also includes questions about substance use, harm reduction knowledge and practices, experiences with the criminal justice system, mental health challenges, and experiences of violence and trauma services (Table 1). The RAC and PRT extensively reviewed this survey to ensure readability and accuracy to capture the various structural factors PLHIV experience in Manitoba. The second questionnaire, the Childhood Trauma Questionnaire- Short [46], is a self-reported inventory that employs Likert scale responses to assess different types of childhood mistreatment and understand the role of historical trauma on life circumstances (Table 1). Finally, the Empowr- Making Decisions Survey [47] (revised shortened version) employs a Likert scale to understand factors related to resilience and decision-making (Table 1). These comprehensive measures will provide a holistic description of the interrelated experiences of HIV care, Covid-19, substance use, PROGRESS indicators, mental health factors, trauma, perceived personal resilience and decision-making of PLHIV in Manitoba. All interviews will take place in a private room to ensure participant

For peer review only - http://bmjopen.bmj.com/site/about/guidelines.xhtml

confidentiality and researchers' safety. Participants will receive 50 CAD cash for their time at the end of the interview. Any transportation costs and childcare services will be reimbursed. Interviews may be broken to several sessions based on participants circumstances and preference.

For service providers, semi-structured interviews will involve 19 open-ended questions (Supplementary Material 5 and Table 1). The interview guide used for service providers has been revised and edited several times by the RAC and key stakeholders. Questions focus on structural and social factors affecting HIV services and the lives of PLHIV. Service providers will also be asked to provide recommendations to improve HIV care. All interviews will occur over Zoom or Microsoft Teams following the University of Manitoba Virtual Platform Guidelines. Confidentiality and security in videoconferencing software will be ensured by only allowing authenticated users to join the meetings and saving meeting recordings in either a University of Manitoba OneDrive or locally to a University of Manitoba device with disk encryption enabled.

The research team will explain the study and obtain consent from all participants before the data collection. If written consent is not possible, research team members will collect verbal consent recorded at the beginning of the interview. Participants will take a copy of the consent form for their records. Sessions are expected to last between one to two and a half hours. Recordings will be encrypted and stored in a password-protected computer. In addition to recordings, research team members may take notes directly on the interview guide or in notebooks. After each interview, research team members will participate in debriefing sessions to increase study rigour.

This study is part of a broader research that was describing the epidemiology of HIV in Manitoba (Manitoba HIV Program Report [8] and manuscripts in preparation). We collected

For peer review only - http://bmjopen.bmj.com/site/about/guidelines.xhtml

from clinical charts sociodemographic and clinical information from 517 people newly diagnosed with HIV between 2018-2021. Data included >90 variables about age, sex, gender, race/ethnicity, rural/urban, substance use (drugs and route), other infectious diseases and non-communicable diseases/conditions and treatments, primary care information, HIV- and Covid-19 related information, and follow-ups.

Data Analysis

All qualitative and quantitative data will be disaggregated by sex and gender.

All qualitative data will be transcribed verbatim using Otter.ai and checked by the research team to ensure accuracy. Transcripts will be entered into NVivo© (version 12) and analyzed using grounded theory methods and sex- and gender-based analysis. Grounded theory provides an iterative framework in which data is collected and analyzed simultaneously by immersing the research members in the data through open coding, code analysis, and further exploration of new themes [49]. Three independent research team members with experience in qualitative analysis will conduct exploratory coding on an initial subset of the transcripts to establish a codebook for each group. Two independent coders will then establish intercoder reliability to increase rigour. Emerging codes will be grouped into larger categories, and themes will be developed to describe the data within a larger framework. During data analysis, peer debriefing will be used with PRT members to share views (e.g., processes, participant engagement) and ideas about codes and themes since this process reduces researcher bias [50]. Negative case analysis will also reduce researcher bias throughout the data analysis process. Finally, an audit trail of decisions through the data collection and analysis will be kept, increasing reproducibility.

For peer review only

For peer review only - http://bmjopen.bmj.com/site/about/guidelines.xhtml

Quantitative data from the three questionnaires will be reported using descriptive statistics. These surveys will complement qualitative data by providing information regarding social and structural inequity factors and provide a broader perspective of the experiences of PLHIV in Manitoba.

Following the syndemic model [33,34], the data collected from clinical charts will provide information about concomitant conditions and how they interact. We will analyze: Demographic characteristics: sex at birth, gender, sexual orientation, race/ethnicity, and age. Living circumstances: houselessness, rural/urban area. Self-reported main HIV exposure factors: heterosexual sex, gay, bisexual, and other men who have sex with men, injection drug/needle use, perinatal acquisition, recipient of blood/blood products, other (which one), and unknown/no identified risk. Clinical diseases and other conditions: STBBI, other comorbidity/condition reported at HIV entry into care, including other infectious diseases non-STBBI, other chronic condition, mental health issues. Disease-disease and social condition-disease interactions include: houselessness (yes/no), injection drug use (yes/no), STBBI at entry into HIV care (yes/no), sex (female/male, there was no intersex reported) and gender (women, men and non-binary), coexisting mental health condition(yes/no). Main outcomes: linkage to HIV care (linked/non/linked) defined as the person who attends an appointment with an HIV clinician within three months of HIV diagnosis, or the person started on antiretroviral treatment within three months of HIV diagnosis, retention in HIV care (yes/no) defined as the person interacting with the HIV care during the follow-up, and undetectable viral load (yes/no) defined as the person has the viral load <200 copies/ml. With this analysis we want to: 1) uncover whether females and women report distinct combinations of syndemic conditions, 2) examine associations between demographic and living status and syndemic class, 3) assess between-group

For peer review only - http://bmjopen.bmj.com/site/about/guidelines.xhtml

differences in linkage to HIV care, retention in HIV care, and undetectable viral load, and 4) test syndemic conditions as a predictor of non-linkage to HIV care, no retention in HIV care, and detectable viral load. We will perform a latent class analysis, followed by a logistic regression if the outcomes are higher than 10%, or Poisson regression if the outcomes are less than 10%.

The quantitative and qualitative data collected prospectively and reported in this paper, will provide information about how people experience their conditions (social, structural and cultural factors unique to each person), and how HIV care and harm reduction services connect to people.

ETHICS AND DISSEMINATION

This project received ethics approval from the University of Manitoba Health Ethics Research Board (HS25572;H2022:218), First Nations Health and Social Secretariat of Manitoba, Nine Circles Community Health Centre, Shared Health Manitoba (SH2022:194), and 7th Street Health Access Centre. As some participants will self-identify as First Nations, Métis or Indigenous, the project will follow Indigenous data sovereignty principles. Among these are the First Nation principles of OCAP®, Manitoba Métis principles of OCAS, the Principles of Ethical Métis Research, and CARE Principles for Indigenous Data Governance, under the leadership of the RAC.

Findings will be disseminated through community-led knowledge translation strategies identified by participants, peers, community members, and community organizations; conference presentations, peer-reviewed journal articles, social media, workshops, and a dedicated webpage (www.alltogether4ideas.org).

DISCUSSION

For peer review only - http://bmjopen.bmj.com/site/about/guidelines.xhtml

Staggering inequities affect the lives of many groups across Canada [51]. To understand health inequities in Manitoba, it is crucial to contextualize the structural conditions that shape health outcomes. This contextualization would benefit from using social determinants of health and a syndemic framework in which health crises such as HIV, Covid-19, and substance use interact with each other and with inequitable social conditions to exacerbate adverse health outcomes in underserved groups [33–35]. In Manitoba, PWID, Indigenous peoples, and people experiencing houselessness are overrepresented in new HIV diagnoses [8]. Similarly, the onset of the Covid-19 pandemic accentuated pre-existing socioeconomic and structural factors, mental health concerns, violence, stigma and discrimination, increased sex and gender disparities, and reduced the resources for STBBI and harm reduction services [29,31]. Complementing qualitative and quantitative data collection with PLHIV and service providers can provide an in-depth understanding of barriers and facilitators to HIV care in Manitoba. Using in-depth semi-structured interviews and questionnaires will enable a broader approach to understanding and addressing Manitoba's various health crises and social inequities. This innovative study uses the World Health Organization Conceptual Framework for Action on the Social Determinants of Health and syndemic frameworks to understand the interrelated experiences of HIV care, Covid-19, substance use and harm reduction, trauma, violence, and other social and structural factors.

This project is driven by participatory engagement processes that centre on the knowledge and expertise of community members, organizations, and people with lived and living experiences to improve the lives, health, and well-being of PLHIV in Manitoba and beyond. Our sample is restricted to people living in metropolitan areas that account for approximately 80% of PLHIV (i.e., Winnipeg and Brandon) in Manitoba and individuals who can dedicate the time necessary for the data collection. However, our purposive recruitment

For peer review only - http://bmjopen.bmj.com/site/about/guidelines.xhtml

strategy to interview people with various backgrounds will ensure we have a representative sample and gain a more comprehensive understanding of the factors that affect the experiences of PLHIV.

Our project will shed light on the growing segment of people missing from the 95-95-95 UNAIDS and Canada's goal [9,10]. Thus, these results will help to advocate for policy and systemic changes to improve the health and well-being of PLHIV in Manitoba.

Acknowledgments: We are mainly thankful of Peer Research Team members Clara Dan, Joel Baliddawa, Lisa Patrick, Nikki Daniels, Robert Russell, Rebecca Murdock, Freda Woodhouse, Ann Favel, Nadia Mancheese, and Marj Schenkels, for their invaluable insights. We are also thankful to members of the Research Advisory committee including Dr. Jacqueline Gahagan, Elder Margaret Lavallee, Veda Koncan, Margaret Bryan, and Jody Jollimore. We thank Heather Pashe (Indigenous Cultural Advisor), some members of Sisters of Fire, Manitoba Harm Reduction Network, Main Street Project, Meeting the Moment, Nine Circles Community Health Centre, Street Connections, 7th Street Health Access Centre and Canadian AIDS Treatment Information Exchange (CATIE) for their ongoing guidance in this project. We are appreciative of all the social workers, nurses, physicians, health education facilitators and service providers in making this study possible. Lastly, thanks to Ms. Mariana Echeverri, communications coordinator of this project, Ms. Echeverri has designed the website, recruitment materials, and all communicational pieces regarding this research.

Author Contributions: The study concept and design were conceived by ZVR, MHB, and YK with input from CS, EV, LL, KM, KD, JS, KT, LM, LI, KK, MP, JB, AK, NP, TM, AM. EV will schedule, collect consent, interview, and complete surveys with participants. CS will

For peer review only - http://bmjopen.bmj.com/site/about/guidelines.xhtml

schedule, collect consent, interview, and complete surveys with service providers. EV, CS and ZVR prepared the first draft of the protocol manuscript. LL, KM, KD, JS, KT, LM, LI, KK, MP, JB, AK, NP, TM, AM, YK, members of the Research Advisory Committee and the Peer Research Team reviewed, edited and revised it critically for important intellectual content.

Competing Interest Statement: There are no competing interests to report.

Funding: This work was supported by the Canadian Institute for Health Research (Grant number EG5-179453), The Manitoba Medical Service Foundation (grant number: 2021-13), The James Farley Memorial Fund. This research was also supported, in part, by the Canada Research Chairs Program for ZVR (Award # 950-232963). Production of this manuscript has been made possible in part through a financial contribution of the Public Health Agency of Canada to the National Collaborating Centre for Infectious Diseases. The views expressed herein do not necessarily represent the views of the Agency.

Data Availability Statement: Data for this study is not available yet since this is a protocol paper. The study interview guides and questionnaires are available in the supplementary materials section.

For peer review only

References

1 Govender RD, Hashim MJ, Khan MA, *et al.* Global Epidemiology of HIV/AIDS: A Resurgence in North America and Europe. *J Epidemiol Glob Health* 2021;**11**:296–301. doi:10.2991/jegh.k.210621.001

2 Coovadia HM, Hadingham J. HIV/AIDS: global trends, global funds and delivery bottlenecks. *Glob Health* 2005;**1**:13. doi:10.1186/1744-8603-1-13

3 Biesma RG, Brugha R, Harmer A, *et al.* The effects of global health initiatives on country health systems: a review of the evidence from HIV/AIDS control. *Health Policy Plan* 2009;**24**:239–52. doi:10.1093/heapol/czp025

4 Hanefeld J. How have Global Health Initiatives impacted on health equity? *Promot Educ* 2008;**15**:19–23. doi:10.1177/1025382307088094

5 Shannon K, Crago A-L, Baral SD, *et al.* The global response and unmet actions for HIV and sex workers. *Lancet Lond Engl* 2018;**392**:698–710. doi:10.1016/S0140-6736(18)31439-9

6 Haddad N, Robert A, Weeks A, *et al.* HIV in Canada—Surveillance Report, 2018. *Can Commun Dis Rep* 2019;**45**:304–12. doi:10.14745/ccdr.v45i12a01

7 Duncan KC, Reading C, Borwein AM, *et al.* HIV Incidence and Prevalence Among Aboriginal Peoples in Canada. *AIDS Behav* 2011;**15**:214–27. doi:10.1007/s10461-010-9792-y

8 Manitoba HIV Program. Manitoba HIV Program Report 2018-2021. 2022.https://mbhiv.ca/wp-content/uploads/2022/11/Report-MB-HIV-Program-FINAL-Nov-30-2022-Reduced-size.pdf

9 UNAIDS. Understanding Fast-Track Accelerating Action To End the Aids Epidemic by 2030. 2015.https://www.unaids.org/sites/default/files/media_asset/201506_JC2743_Understanding_FastTrack_en.pdf (accessed 16 Aug 2022).

10 Public Health Agency of Canada. Canada takes action by endorsing global declaration on Undetectable = Untransmittable (U=U). 2022.https://www.canada.ca/en/public-health/news/2022/07/canada-takes-action-by-endorsing-global-declaration-on-undetectable--untransmittable-uu.html (accessed 9 Mar 2023).

11 Center for Disease Control and Prevention (CDC). Understanding the HIV Care Continuum. 2017.https://www.cdc.gov/hiv/pdf/library/factsheets/cdc-hiv-care-continuum.pdf (accessed 9 Aug 2022).

12 Tso LS, Best J, Beanland R, *et al.* Facilitators and barriers in HIV linkage to care interventions: a qualitative evidence review. *AIDS Lond Engl* 2016;**30**:1639–53. doi:10.1097/QAD.0000000000001101

For peer review only - http://bmjopen.bmj.com/site/about/guidelines.xhtml

13 Dombrowski J. Linkage to Care. Natl. HIV Curric. 2020.https://www.hiv.uw.edu/go/screening-diagnosis/linkage-care/core-concept/all (accessed 10 Aug 2022).

14 Hall BJ, Sou K-L, Beanland R, *et al.* Barriers and Facilitators to Interventions Improving Retention in HIV Care: A Qualitative Evidence Meta-Synthesis. *AIDS Behav* 2017;**21**:1755–67. doi:10.1007/s10461-016-1537-0

15 Argento E, Strathdee SA, Goldenberg S, *et al.* Violence, trauma and living with HIV: Longitudinal predictors of initiating crystal methamphetamine injection among sex workers. *Drug Alcohol Depend* 2017;**175**:198–204. doi:10.1016/j.drugalcdep.2017.02.014

16 Scully EP. Sex Differences in HIV Infection. *Curr HIV/AIDS Rep* 2018;**15**:136–46. doi:10.1007/s11904-018-0383-2

17 Everett BG. Sexual Orientation Disparities in Sexually Transmitted Infections: Examining the Intersection Between Sexual Identity and Sexual Behavior. *Arch Sex Behav* 2013;**42**:225–36. doi:10.1007/s10508-012-9902-1

18 Ostrach B, Singer M. At special risk: Biopolitical vulnerability and HIV/STI syndemics among women. *Health Sociol Rev* 2012;**21**:258–71. doi:10.5172/hesr.2012.21.3.258

19 Statistics Canada. Family Violence in Canada: A Statistical Profile. 2011.https://www150.statcan.gc.ca/n1/pub/85-224-x/85-224-x2010000-eng.pdf

20 Tutty LM, Nixon KL. #MeToo on the Canadian Prairies: Raising Awareness of Sexual Assaults and Mental Health in Women Abused by Intimate Partners. *Violence Women* 2022;**28**:1398–419. doi:10.1177/10778012211032699

21 Tutty LM, Radtke HL, Ateah CA, *et al.* The Complexities of Intimate Partner Violence: Mental Health, Disabilities, and Child Abuse History for White, Indigenous, and Other Visible Minority Canadian Women. *J Interpers Violence* 2021;**36**:1208–32. doi:10.1177/0886260517741210

22 Tutty LM, Radtke HL, Thurston WEB, *et al.* The Mental Health and Well-Being of Canadian Indigenous and Non-Indigenous Women Abused by Intimate Partners. *Violence Women* 2020;**26**:1574–97. doi:10.1177/1077801219884123

23 Nixon KL, Tutty LM, Radtke HL, *et al.* Protective Strategies of Mothers Abused by Intimate Partners: Rethinking the Deficit Model. *Violence Women* 2017;**23**:1271–92. doi:10.1177/1077801216658978

24 Chenneville T, Gabbidon K, Hanson P, *et al.* The Impact of COVID-19 on HIV Treatment and Research: A Call to Action. *Int J Environ Res Public Health* 2020;**17**:E4548. doi:10.3390/ijerph17124548

For peer review only - http://bmjopen.bmj.com/site/about/guidelines.xhtml

25 Tsamakis K, Tsiptsios D, Ouranidis A, *et al.* COVID-19 and its consequences on mental health (Review). *Exp Ther Med* 2021;**21**:244. doi:10.3892/etm.2021.9675

26 Salmon S, Taillieu TL, Fortier J, *et al.* Pandemic-related experiences, mental health symptoms, substance use, and relationship conflict among older adolescents and young adults from Manitoba, Canada. *Psychiatry Res* 2022;**311**:114495. doi:10.1016/j.psychres.2022.114495

27 Almeida M, Shrestha AD, Stojanac D, *et al.* The impact of the COVID-19 pandemic on women's mental health. *Arch Womens Ment Health* 2020;**23**:741–9. doi:10.1007/s00737-020-01092-2

28 Castellini G, Tarchi L, Cassioli E, *et al.* Attachment Style and Childhood Traumatic Experiences Moderate the Impact of Initial and Prolonged COVID-19 Pandemic: Mental Health Longitudinal Trajectories in a Sample of Italian Women. *Int J Ment Health Addict* Published Online First: 17 March 2022. doi:10.1007/s11469-022-00798-x

29 Manitoba Harm Reduction Network, The National Collaborating Centre for Infectious Diseases. Report of an Evaluation of Manitoba Harm Reduction Network Services in the Context of the COVID-19 Pandemic. 2022.https://nccid.ca/wp-content/uploads/sites/2/2022/02/MHRN-report_2022_Final.pdf

30 European Monitoring Centre for Drugs and Drug Addiction, editor. Harm reduction: evidence, impacts and challenges. 2010.https://www.emcdda.europa.eu/system/files/publications/555/EMCDDA-monograph10-harm_reduction_final_205049.pdf

31 Public Health Agency of Canada. Survey of the impact of COVID-19 on access to STBBI-related health services including harm reduction services among people who use drugs or alcohol in Canada. 2021.https://health-infobase.canada.ca/datalab/covid-19-stbbi-services.html (accessed 22 Jul 2022).

32 Epidemiology and Surveillance Unit, Government of Manitoba. Substance Related Harms: Epidemiologist's Summary. Ment. Health Community Wellness. 2022.https://www.gov.mb.ca/mh/index.html (accessed 9 Mar 2023).

33 Singer M. A dose of drugs, a touch of violence, a case of AIDS: Conceptualizing the Sava Syndemic. *Free Inq Creat Sociol* 2000;**28**:13–24.

34 Singer M, Bulled N, Ostrach B, Mendenhall E. Syndemics and the biosocial conception of health. Lancet. 2017 Mar 4;389(10072):941-950. doi: 10.1016/S0140-6736(17)30003-X. PMID: 28271845.

For peer review only - http://bmjopen.bmj.com/site/about/guidelines.xhtml

35 World Health Organization. A conceptual framework for action on the social determinants of health. World Health Organization 2010. https://apps.who.int/iris/handle/10665/44489 (accessed 24 Mar 2023).

36 Andermann A. Outbreaks in the age of syndemics: New insights for improving Indigenous health. *Can Commun Dis Rep* 2017;**43**:125–32. doi:10.14745/ccdr.v43i06a02

37 Else-Quest NM, Hyde JS. Intersectionality in Quantitative Psychological Research: II. Methods and Techniques. *Psychol Women Q* 2016;**40**:319–36. doi:10.1177/0361684316647953

38 Reason P, Bradbury H, editors. *The Sage handbook of action research: participative inquiry and practice*. 2nd ed. London ; Thousand Oaks, Calif: : SAGE Publications 2008.

39 Allen W. Participatory action research provides for multiple benefits - Learning for Sustainability. Learn. Sustain. 2016.https://learningforsustainability.net/post/par/ (accessed 25 Jul 2022).

40 Organizing engagement. Participatory Action Research and Evaluation. Organ. Engagem. 2019.https://organizingengagement.org/models/participatory-action-research-and-evaluation/ (accessed 10 Aug 2022).

41 Johnston P. *Native children and the child welfare system*. Toronto [Ont.]: : Canadian Council on Social Development in association with James Lorimer & Co. 1983. https://www.deslibris.ca/ID/413616 (accessed 4 Apr 2023).

42 National Collaborating Centre for Aboriginal Health. Indigenous Children and the Child Welfare System in Canada. 2017.https://www.nccih.ca/docs/health/FS-ChildWelfareCanada-EN.pdf

43 Statistics Canada. Focus on Geography Series, 2016 Census. 2017.https://www12.statcan.gc.ca/census-recensement/2016/as-sa/fogs-spg/Facts-PR-Eng.cfm?TOPIC=9&LANG=Eng&GK=PR&GC=46 (accessed 27 Jul 2022).

44 McGregor HE, Marker M. Reciprocity in Indigenous Educational Research: Beyond Compensation, Towards Decolonizing. *Anthropol Educ Q* 2018;**49**:318–28. doi:10.1111/aeq.12249

45 Logie C. *Working with Excluded Populations in HIV: Hard to Reach or Out of Sight?* Cham: : Springer International Publishing 2021. doi:10.1007/978-3-030-77048-8

46 Bernstein D, Stein J, Newcomb M, *et al.* Development and validation of a brief screening version of the Childhood Trauma Questionnaire. 2003;**27**:169–90. doi://doi.org/10.1016/S0145-2134(02)00541-0

For peer review only

For peer review only - http://bmjopen.bmj.com/site/about/guidelines.xhtml

47 Rogers ES, Ralph RO, Salzer MS. Validating the empowerment scale with a multisite sample of consumers of mental health services. *Psychiatr Serv Wash DC* 2010;**61**:933–6. doi:10.1176/ps.2010.61.9.933

48 O'Neill J, Tabish H, Welch V, *et al.* Applying an equity lens to interventions: using PROGRESS ensures consideration of socially stratifying factors to illuminate inequities in health. *J Clin Epidemiol* 2014;**67**:56–64. doi:10.1016/j.jclinepi.2013.08.005

49 Charmaz K. *Constructing Grounded Theory A Practical Guide Through Qualitative Analysis*. SAGE Publications 2006.

50 Heilman J, Figg C, Wenrick M, *et al.* Implications and Benefits of a Long-Term Peer Debriefing Experience on Teacher Researchers. *Brock Educ J Educ Res Pract* 2010;**19**:20–35. doi:10.26522/brocked.v19i1.118

51 Public Health Agency of Canada. Key health inequalities in Canada: a national portrait. 2018.http://epe.lac-bac.gc.ca/100/201/301/weekly_acquisitions_list-ef/2019/19-06/publications.gc.ca/collections/collection_2019/aspc-phac/HP35-109-2018-1-eng.pdf (accessed 17 Aug 2022).

For peer review only

For peer review only - http://bmjopen.bmj.com/site/about/guidelines.xhtml

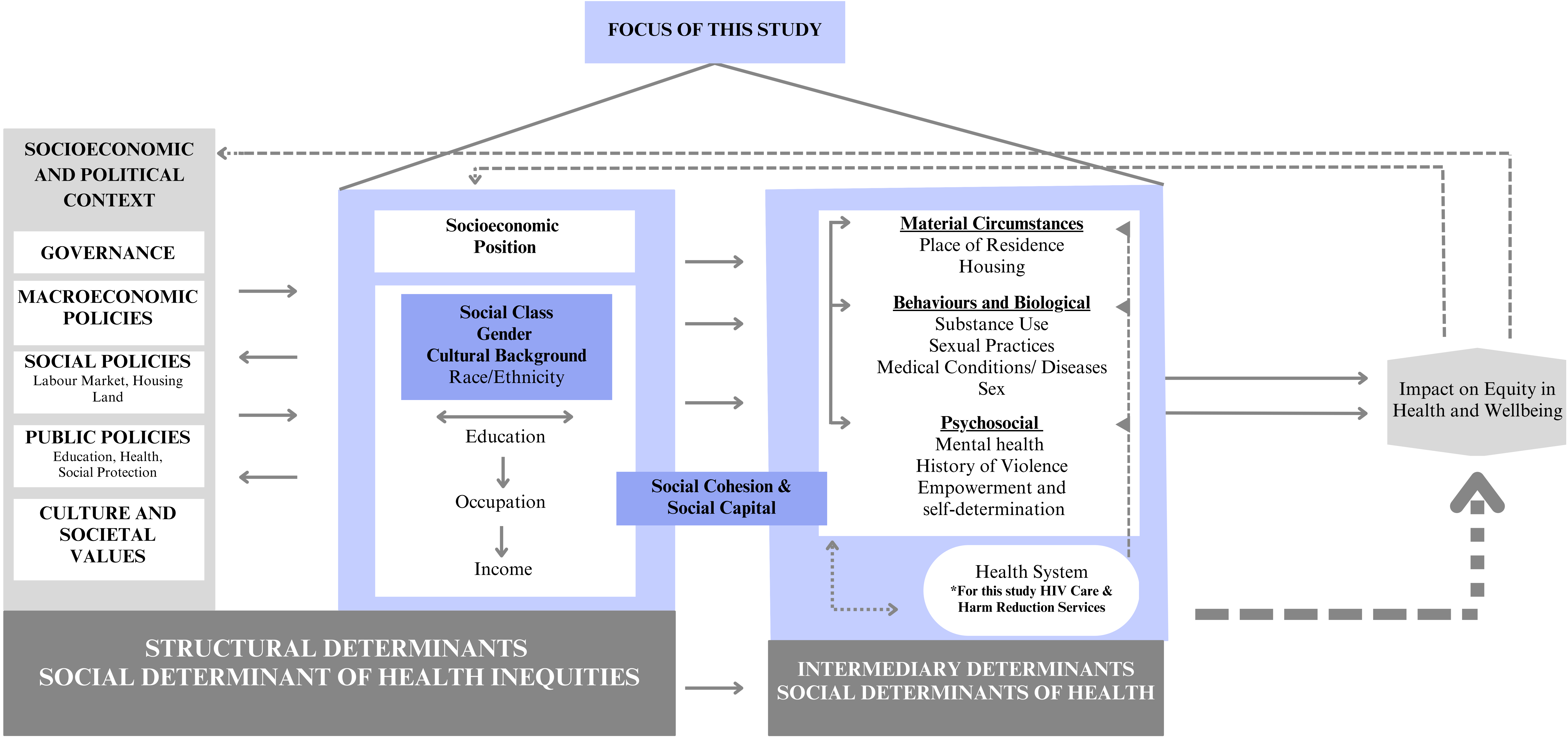
FOCUS OF THIS STUDY
SOCIOECONOMIC AND POLITICAL CONTEXT
GOVERNANCE
MACROECONOMIC POLICIES
SOCIAL POLICIES
Labour Market, Housing Land
PUBLIC POLICIES
Education, Health, Social Protection
CULTURE AND SOCIETAL VALUES
Socioeconomic Position
Social Class
Gender
Cultural Background
Race/Ethnicity
Education
Occupation
Income
Social Cohesion & Social Capital
Material Circumstances
Place of Residence
Housing
Behaviours and Biological
Substance Use
Sexual Practices
Medical Conditions/ Diseases
Sex
Psychosocial
Mental health
History of Violence
Empowerment and self-determination
Health System
*For this study HIV Care & Harm Reduction Services
Impact on Equity in Health and Wellbeing
STRUCTURAL DETERMINANTS
SOCIAL DETERMINANT OF HEALTH INEQUITIES
INTERMEDIARY DETERMINANTS
SOCIAL DETERMINANTS OF HEALTH

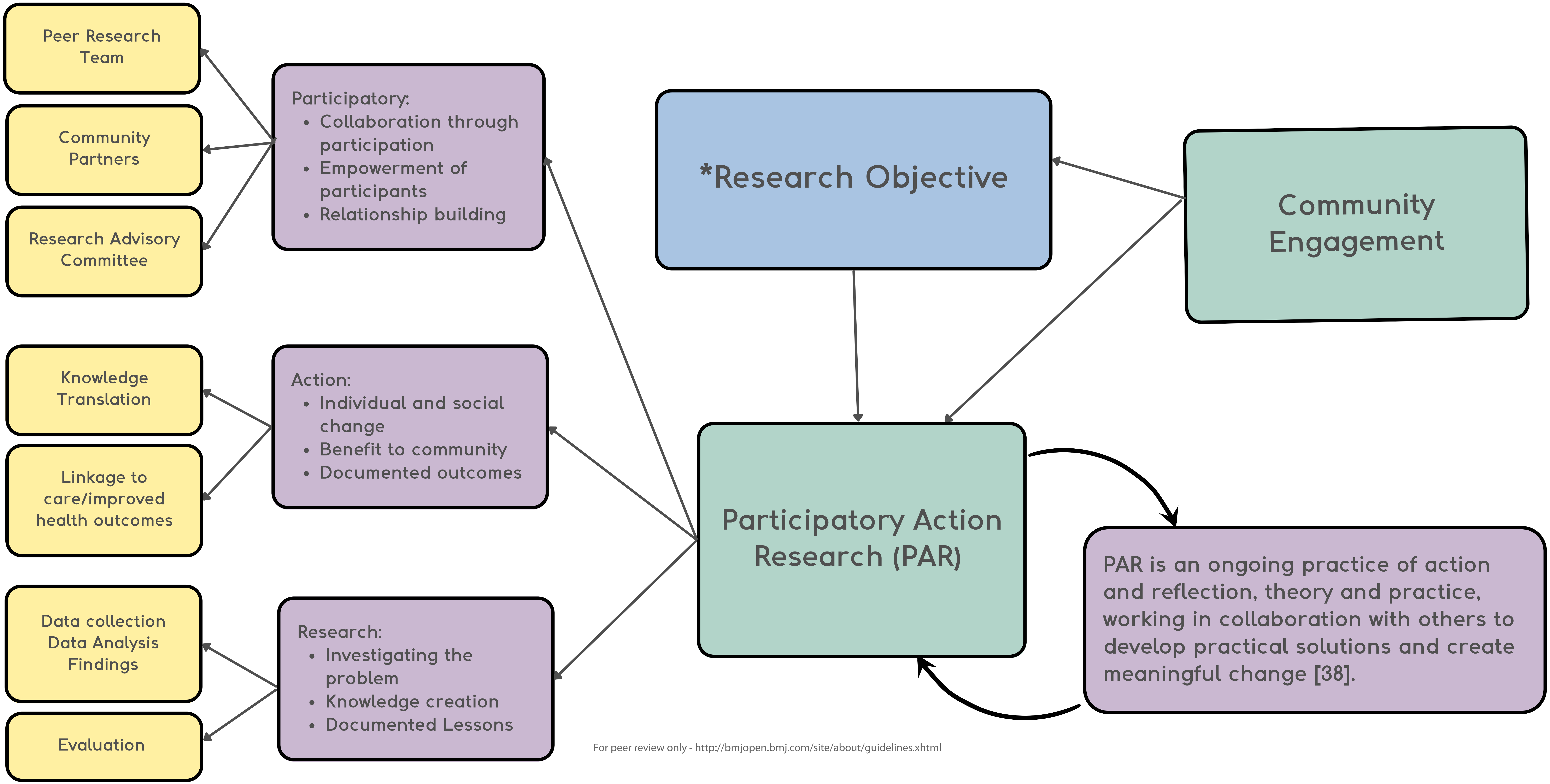
Peer Research Team
Community Partners
Research Advisory Committee
Participatory:
• Collaboration through participation
• Empowerment of participants
• Relationship building
*Research Objective
Community Engagement
Knowledge Translation
Linkage to care/improved health outcomes
Action:
• Individual and social change
• Benefit to community
• Documented outcomes
Participatory Action Research (PAR)
PAR is an ongoing practice of action and reflection, theory and practice, working in collaboration with others to develop practical solutions and create meaningful change [38].
Data collection Data Analysis Findings
Evaluation
Research:
• Investigating the problem
• Knowledge creation
• Documented Lessons

Terms of Reference for the Research Advisory Committee

Background:

In Manitoba, injection drug use is the most common mode of HIV transmission since 2018. Those who inject drugs are more likely to test positive for HIV, syphilis and Hepatitis C, often at the same time. This is called a "syndemic" (many epidemics at the same time). For the last three years, syphilis infections have become rampant in Manitoba. If not found and treated, syphilis can cause devastating infection, including transmission to newborns. In addition to HIV and syphilis there are 11 other infections transmitted by sexual activity or blood products (called STBBI).

For some people, a convergence of socio-economic factors, mental health concerns, violence and exploitation, is driving a syndemic of new HIV and other STBBI cases, with disproportionate representation of women who inject drugs. Methamphetamine use is growing in Manitoba and is known to affect access to care and treatment. These determinants of health and disparities are exacerbated by COVID-19 related public health measures, as resources for testing and contact tracing for Covid-19 have resulted in decreased capacity for service providers to respond to STBBI and distribute harm reduction services. It is unknown how both biological sex differences and gender intersect with living conditions including experiences of violence and injection drug use.

This study seeks to know:

1. How many people in Manitoba (by sex, gender and other identifiers) living with HIV have other sexually transmitted and blood-borne infections (STBBI), before and during Covid-19?
2. Of those living with HIV, whom are most at risk of acquiring other STBBI, and why?
3. What are the barriers and gaps that put people at risk for infection and prevent them from getting appropriate care and treatment before and during Covid-19?
4. What are the resilience factors and promising practices that are associated with successful engagement of people living with HIV and who inject methamphetamine that endured during the Covid-19 pandemic?

Gaining an in-depth understanding of the experiences of women, men and non-binary persons living with HIV who use and don't use drugs can help us identify unique gaps as well as resilience factors that can inform and improve the Manitoba HIV Program, and result in tailored testing and appropriate clinical and program responses.

Principal Investigators:

Dr. Zulma Rueda. University of Manitoba. Email: zulma.rueda@umanitoba.ca
Dr. Yoav Keynan University of Manitoba. Email: yoav.keynan@umanitoba.ca
Ms. Margaret Haworth-Brockman, University of Manitoba. Email: Margaret.Haworth-Brockman@umanitoba.ca

Advisory Committee's Role and Purpose

The Advisory Committee will advise the research team on the following aspects of the project:

- ✓ Research approach and community-based methods
- ✓ Guidance the implementation of research.
- ✓ Promotion of the research study.

For peer review only

- ✓ Advice on community engagement plans.
- ✓ Interpretation of findings.
- ✓ Help to identify and strategize any potential solutions to community issues that may arise.
- ✓ At all times, strictly maintain confidentiality for all aspects of the research project as outlined in the University of Manitoba *Access and Privacy policy* and *Access and Privacy Procedure.*

- ✓ **<u>Input and advice from the Advisory Committee will help to ensure that</u>**:
  - ✓ The project/study is culturally appropriate for the diverse communities involved.
  - ✓ The research team conducts the project in the communities' best interests.
  - ✓ The research results and knowledge will be shared appropriately with communities and stakeholders.
  - ✓ Action plans associated with the research receive community input and are appropriate to community needs and concerns.

All Advisory Committee members will be eligible for compensation for their time involved. We expect there to be about 5-7 meetings over 12 months, with one or two meetings being in the early stages of the project development.

<u>Responsibilities from the research team:</u>
1. Ensure the ethical conduct of the research study.
2. Provide accurate information related to the project.
3. Investigate and answer the suggestions of the research advisory committee.

<u>Disclosures:</u>
- ✓ All member will declare any real or perceived conflict of interest at the outset of any meeting for management in accordance with the University of Manitoba *Conflicts of Interest Policy and Conflict of Interest Procedures.*
- ✓ All research project conducted within the University of Manitoba is governed by the *Responsible Conduct of Research Policy, Responsible Conduct of Research – Code of Research Ethics Policy, Responsible Conduct of Research – Investigation Procedure.*
- ✓ This research is funded by the Canadian Institute of Health Research; therefore, we follow and adhere to the *Tri-Agency Framework: Responsible Conduct of Research, including the framework and policies of Chapter 9: Research Involving the First Nations, Inuit and Métis Peoples of Canada.*

<u>Term</u>
This research is initially funded for one year.

<u>Memberships</u>
The Committee will be composed by the following members, in addition to the 3 principal investigators. Our meetings will be opened in a Good Way with the help of an Elder.

The number and composition of members comprises of:
1. Five or six community members – we are actively recruiting advisors now. We are inviting members who include:

For peer review only

a. Person who lives with HIV
b. Person who has current or former experience with injection drug use
c. Women or female who have experienced sexual assault, sexual violence, experiencing violence or sexual exploitation
d. Person who is experiencing homelessness or who are unsheltered
e. Person who identify themselves as 2SLGBTQQIA+
f. Person who belong to Indigenous population or racialized communities

2. Elder Margaret Lavallee, Elder in Residence, Ongomiizwin
3. Julianne Sanguins. Adjunct Scientist at the Manitoba Centre for Health Policy, and Assistant Professor at the Department of Community Health Sciences, University of Manitoba.
4. Tara Faye Myran, Metis Community member.
5. Ann Favel, Person with lived experience. Member of Sisters of Fire
6. Jaqueline Flett, Person with lived experience. Member of Sisters of Fire
7. Marj Schenkels, Person with lived experience. Member of Sisters of Fire
8. Nikki Daniels. Community member with lived experience.
9. Jody Jollimore. Executive Director at CATIE
10. Margaret Bryan. Manitoba Harm Reduction Network
11. Jacqueline Gahagan. Associate Vice-President, Research, Mount Saint Vincent University. Halifax, Nova Scotia.
12. Adrienne FA Meyers, Associate Director of the JC Wilt Infectious Diseases Research Centre
13. Neora Pick, Clinical Professor in the Infectious Diseases division of the Department of Medicine at the University of British Columbia, and Medical Director at the Oak Tree Clinic.
14. Veda Koncan, Projects Manager, Manitoba Harm Reduction Network
15. Chantal Daniels, Elder Support, Indigenous Institute of Health & Healing, Ongomiizwin

For peer review only

Meeting frequency

Meetings will be called by the Project Manager.
The committee will meet for five or seven 2-hour meetings either in person in Winnipeg or via teleconference/web-based technology for members residing outside Winnipeg.

Sitting fees

Research team will provide $20 CAD/per hour honorariums for people who are eligible for honorarium
Research team will cover costs for transportation for people residing in Winnipeg

Contact:

Dr. Zulma Rueda. University of Manitoba. Email: zulma.rueda@umanitoba.ca
Dr. Yoav Keynan University of Manitoba. Email: yoav.keynan@umanitoba.ca
Ms. Margaret Haworth-Brockman, University of Manitoba. Email: Margaret.Haworth-Brockman@umanitoba.ca

Cheryl Sobie. Research project coordinator. University of Manitoba. Email: Cheryl.Sobie@umanitoba.ca

For peer review only

Peer Terms of Reference

Purpose:

We want to engage people with lived experience as peers in our project and work collaboratively to ensure all aspects of the research are shaped and guided by the perspectives of peers with lived experience. Peer engagement will inform the entire research process, from conducting research interviews, to skill sharing, workshops, and developing educational materials to share the results of our research. We recognize the unique knowledge and expertise of community members with lived experience, and we seek your guidance and input for our research study.

Background Information:

We are peers, clinicians and researchers who want to understand infections transmitted by sex or blood in people living with HIV, who may use drugs, as well as why people living with HIV are not getting the care they need. We are interested in knowing which factors, barriers and gaps prevent people from accessing care. We will also look for resilience factors that keep people linked to care.

We are interested in better understanding the impact of the COVID-19 pandemic and how it may have affected people newly diagnosed with HIV (between 2018-2021) in accessing health care and other resources. We want to know things such as:

- What was it like navigating the health care system during the pandemic?
- What are ways we can make things better for people to be linked to health care?
- What things are working well for people that keep them going to get health support and resources before and during the COVID-19 pandemic?

Project Time Period:

July 2022 – March 2023

Peer Roles available:

1. Peer Research Advisory Committee Member
2. Peer Support Worker
3. Peer Group Team Member

Role (1) Peer Research Advisory Committee Member (see Research Advisory Committee Terms of Reference Document). Key items to note:

How does this work?
You will join our committee which includes peers like you and some researchers. Together you will advise the research team about the best ways to:

- involve other people in the research study (using posters, or word of mouth, or in certain places);

For peer review only

- pay or compensate them for their time (for example, taxi coupons, bus passes, cash, or pre-paid VISAs);
- questions that are appropriate in interviews;
- how to understand what we find from the results;
- how to share the results of the study with the community;
- other education that the community or health care providers need to know. There may be other things you provide advice on.

Meeting frequency:

The Research Advisory Committee will meet for five or seven 2-hour meetings either in person in Winnipeg or via teleconference/web-based technology for members residing outside Winnipeg.

Compensation:

The research team will provide $20 CAD/per hour honorarium for peer research advisory committee members. Research team will cover costs for transportation (bus fare, taxi) for people residing in Winnipeg to attend the meeting.

Role (2) Peer Support Worker:

How does this work?

- Provide peer support for participants (people living with HIV) during research interview
- You will sit in the interview with participant and research team member to provide support as needed
- When we are scheduling interviews, we will ask participants if they would like a peer support worker at their interview
- Must sign confidentiality agreement and maintain confidentiality at all times
- We plan to interview 20-30 people; we are unsure at this point how many will require support
- The interview will take place in community settings such as Nine Circles, HSC HIV Clinic or another community location as determined by the participant and research team member

Time Required:

- 2-3 hours per interview session
- We anticipate interviews will take place between the end of August - October 2022

Role (3) Peer Research Team Member

How does this work?

For peer review only

- The purpose of the peer research team is to bring together community members with lived experience to work together to conduct research (interviews, data analysis, review findings), develop ideas for how we will share the results of our research, review comments from the advisory committee, and provide guidance and direction on the research for recruitment, community engagement, etc.
- The peer team is separate from the advisory board; however, a peer group member may also sit on the advisory board.
- The work of the peer team will include items such as:
    - Develop research skills and conduct research interviews, support data analysis and review findings of research
    - Discuss issues raised by the advisory board
    - Develop ideas and collaborate to prepare educational material for the community based on the results and findings of the research
    - To learn from each other either by skill sharing (i.e., doing computer courses provided by university, learning new skills related to grant writing/proposal development, learning technologies such as Zoom, etc.); we want to hear from YOU what you want to learn about
    - To advocate and share with the public what it is that is needed in Manitoba to break the stigma of HIV and drug use
- The peer team will consist of ten community members with lived experience
- Members will be selected by members that want to participate. If too many people apply, we will narrow out the group by selecting someone from each of the criteria in line with the research

Meetings:

<u>Meeting Frequency & Location</u>

The peer team will meet every two to three weeks for two hours. We will meet in person wherever possible at the University of Manitoba Bannatyne campus, or outside at a park. Should things change depending on the COVID-19 situation and safety guidelines we may have to meet online using Zoom or Teams.

<u>Comfort & Safety</u>

We will create a safe space for all group members to participate in, which includes psychological safety and cultural safety. Members can participate to the maximum comfortable amount.

We reject any form of discrimination or racism. We are committed for a safe and inclusive environment for everyone to participate. We do not judge each other and will respect each other at all times. We are committed to making your participation as accessible as possible. If there is anything you need from us to support you, please let us know.

<u>Activities</u>

For peer review only

Meetings will focus on relationship building, skill sharing, and project discussions. Once the data collection is completed, we will then turn our attention to creating educational materials to share the results of the research.

In addition to the research and skill sharing activities we want to incorporate fun activities or cultural activities such as beading, drum circles, bringing an Elder, playing games, sharing food together, music, etc.

Supplies

At each meeting:

- We will provide light lunches or snacks and drinks
- Cover any training and education costs
- Provide supplies for making educational materials
- Bring materials required for any activities as discussed

Compensation:

The peer group role will be compensated with an honorarium in the amount of $20/hour cash and bus tickets for transportation will be provided. Childcare will be compensated as needed $10 per hour. No money other than honorarium's will be received by peer members.

- ✓ **Confidentiality**- all team members will respect the confidentiality of other team members and any items discussed within group meetings.
- ✓ All members will declare any real or perceived conflict of interest at the outset of any meeting for management in accordance with the University of Manitoba *Conflicts of Interest Policy and Conflict of Interest Procedures.*
- ✓ All research project conducted within the University of Manitoba is governed by the *Responsible Conduct of Research Policy, Responsible Conduct of Research – Code of Research Ethics Policy, Responsible Conduct of Research – Investigation Procedure.*
- ✓ This research is funded by the Canadian Institute of Health Research; therefore, we follow and adhere to the *Tri-Agency Framework: Responsible Conduct of Research, including the framework and policies of Chapter 9: Research Involving the First Nations, Inuit, and Métis Peoples of Canada.*

For peer review only

Covid-19 Pandemic Concealing a Syndemic of Concern: Sex, Gender, Methamphetamine and Sexually Transmitted and Blood Borne Infections in People Living with HIV in Manitoba

Participant Interview Question Guide

To be completed by facilitator	***Facilitator Name***		
Participant ID:			
Participating Organization:			
Location:			
Interview date:			
Interview time:	Start	End	Duration
Confirmation of consent signed:			

For peer review only

Thank you for agreeing to speak with me today. My name is [INSERT] and my pronouns are INSERT] and this is my colleague [introduce themselves]. My role with this project is as a [Research Assistant, Research Associate, Research Coordinator]. Is there anything you need to do to prepare yourself before we get started? We anticipate the interview and short surveys will take between 1-2 hours to complete. We can take a break during the question portion of this interview if you need to, and we will take a short break after the interview before we begin the surveys. Please let me know at any time if you need to step away for a few minutes.

[NOTE: This next section only applies to people who will not have booked an interview appointment and are a walk-up participant]

Before I explain the purpose and what we will do today I want to check in with you about support we can offer you today and I have three questions I would like to ask you:

1. ***Would you be interested in having a peer support to sit with you for your interview? The purpose of a peer support is only to provide emotional support and they will maintain confidentiality of anything we discuss today.***
2. ***Would you be interested in having cultural support? There is an Elder available who you can speak with at any time if you need a break during the interview, at the break between the interview and surveys or at the end of the interview.***

For peer review only - http://bmjopen.bmj.com/site/about/guidelines.xhtml

3. ***Are you interested in speaking with a psychological support person at the break or after your interview today? They are available anytime if you need to stop the interview to debrief or check in with them.***

Before we start the formal interview questions, I would like to remind you that we are here to speak about your experiences in finding and receiving HIV and STBBI-related health care and services. Our aim is to improve HIV and other services for everyone. We are interviewing people with many stories and, perhaps, some questions may not relate to your life experiences. We appreciate your responses since many of these questions are personal. There are some questions about substance use that may or may not apply to you. The reason we are asking these questions is because our research has shown that in Manitoba, the main way people get HIV is through injection drug use but that is by no means the only way people get HIV. We understand that some people may feel shame or stigma about having HIV or using drugs, which is why we have tried to create a safe and comfortable space to listen to your experiences. It is completely up to you if you want to answer the questions or not.

You have been asked to participate in this interview as you:

1) *Are 18 years or older*
2) *Have received a diagnosis for HIV between 2018-2021*
3) *Are a resident of Manitoba*

Do all of these statements apply to you? ***Y / N***

[If no, relay that they are not eligible to participate in the study. Thank them for their time, and end interview].

For the purpose of our discussion, STBBI services may include testing, care and counselling for HIV, Hepatitis C, syphilis, other sexually transmitted infections like Chlamydia, or gonorrhoea.

We take your information seriously which is why we also want to remind you that everything you share today will be kept confidential. This means that we will not disclose any information you share with us about drug use, engaging in sex work or anything else that could be perceived as "criminal" with authorities. The only reason we would ever have to break confidentiality is if there is a direct risk/danger to your health and safety or that of someone who is under 18. We would like to record this interview to make sure the information is accurate. This recording will be kept in a secure location and destroyed after this study is over. This recording will not be shared.

For your participation today, we will give you a $50 honorarium in the form of cash and bus fare or money to cover a taxi ride to attend the interview. Additionally, we have several items that you can choose from as a gift and a form of appreciation for the valuable insight you have shared with us today.

For peer review only - http://bmjopen.bmj.com/site/about/guidelines.xhtml

Covid-19 Pandemic Concealing a Syndemic of Concern: Sex, Gender, Methamphetamine and Sexually Transmitted and Blood Borne Infections in People Living with HIV in Manitoba

I would like to start with some questions about how things generally are for you, but before we start are there any questions you want to ask?

1. **Could you please tell me about yourself?**
    a. PROBES
        i. How are things going for you right now?
        ii. How are you feeling coming in today?

2. **What does a typical week (day) look like for you?**
    a. PROBES
        i. Daily routine
        ii. Work/volunteer
        iii. Medical programs/services
        iv. Time with friends/family
        v. Time with caring for friends/family

3. **How are things going with your health right now?**
    a. PROBES- ask about:
        i. Physical health
        ii. Mental health
        iii. Emotional health
        iv. Spiritual health
        v. Financial health

Now I would like for us to talk about the HIV care and other health services that you receive.

4. **What was your experience when you were first diagnosed with HIV?**
    a. PROBES
        i. Any available support or counselling?
        ii. Were you given enough information about your options? (i.e. explained about your rights, how to access to care, follow up, linkage to doctor, treatment options?)
        iii. Did you learn about how HIV is transmitted? And how to prevent transmission?
        iv. Did you receive information about importance of treatment?

For peer review only - http://bmjopen.bmj.com/site/about/guidelines.xhtml

Covid-19 Pandemic Concealing a Syndemic of Concern: Sex, Gender, Methamphetamine and Sexually Transmitted and Blood Borne Infections in People Living with HIV in Manitoba

5. **What health services do you currently receive for HIV health care?**
   a. PROBES,
      i. Attend clinic appointments, see a NP, take medication, regular blood testing, etc. (Linked to Care)
      ii. Alternative medicine such as traditional or cultural medicine
      iii. Anything else?
   b. **If not linked to care move to question 7**

6. **What do you like about the HIV health care services you receive?**
   a. PROBES
      i. Relationship with doctor/ nurse/ social worker
      ii. Accessibility of location to receive services
      iii. Welcoming environment at service provider location
      iv. Any other reason?

7. **What problems have you encountered when accessing HIV care?**
   a. PROBES
      I. Stigma, discrimination from service providers
         i. Drug use, unhoused, gender identity
         ii. If yes, please tell me more about that. Where does this discrimination occur?
      II. Transportation, accessibility challenges
      III. Lack of childcare to get to attend appointments or children are not able to accompany to appointment
      IV. Income, lack of money to pay for medication
      V. Other challenges are more important to deal with
      VI. Lack of access to appointments, lengthy wait times to receive care, etc.
      VII. Mental health challenges
      VIII. Any other things you don't like?

8. **Could you please walk me through a time when you needed HIV care but you could not get it?**
   a. PROBES
      i. Ask participant to share a specific experience related to a barrier
      ii. If you were diagnosed before the pandemic (2018-2019), did you experience similar or different challenges before or during the COVID-19 pandemic (2020-current)?

For peer review only - http://bmjopen.bmj.com/site/about/guidelines.xhtml

9. Has the COVID-19 pandemic impacted the HIV health services you use? If yes, in what ways?

   a. PROBES

      i. Availability of appointments, shortage of staff at clinics, services not accessible, staff changes, etc.
      ii. Increased fear/risk due to COVID-19 in health care settings
      iii. Transportation issues
      iv. Money, financial challenges
      v. Use of substances
      vi. Mental health challenges/and or lack of support for mental health

10. How safe do you feel in or around health care settings?

   a. PROBES

      i. In the neighborhood around the clinic/hospital
      ii. Around security personnel such as police, hospital security guards
      iii. Fear of Child & Family Services, removal of children, or fear of other government agencies/actors
      iv. Lack of privacy to discuss health status (i.e., ER visit)
      v. <u>Not safe</u> due to perceived gender identity, race/ethnicity
      vi. Getting to your appointment- location of clinic, transportation

11. What are things that help you to get the health care you need?

   a. PROBES

      i. Positive relationship with doctor/health professionals
      ii. Welcoming and supportive environment at health centres, health centres specific to my needs (i.e. gender specific, LGBTQ+)
      iii. Availability/hours
      iv. Location within the city
      v. Support (personal or peer) to attend health appointments

12. What changes do you think would make it easier for you (and other people living with HIV) to access care?

   a. PROBES

      i. Transportation support, financial support
      ii. Peer support (i.e., someone to accompany you to appointments)
      iii. Better accessibility (e.g., different sites for treatments, availability of appointments, options for care)
      iv. Access to counselling and/or mental health support
      v. Addressing stigma
      vi. Different medication with less side effects
      vii. Access to material support (housing, food, etc.)
      viii. Bundling more services in one appointment- seeing multiple professional, etc. primary care physician, gynaecologist, mental health professional,

For peer review only - http://bmjopen.bmj.com/site/about/guidelines.xhtml

ix. Addressing multiple health concerns at each appointment- either related to HIV or other health issue

I have a few more questions before we end the interview, they are related to substances you may use. These questions may not apply to you, and we want to assure you that there is no judgment based on your use of substances. These questions will help us find ways to better support people who are living with HIV who also may use substances. I also want to remind you that you do not have to answer any questions you do not feel comfortable with, and that all of your answers are confidential.

13. Do you have any experience with using substance?
IF YES, ask the following questions. If no, move to QUESTION 18

14. How old were you when you started using substances?
- a. PROBE:
  - i. What substance(s) did you start with?

15. How would you describe your substance use when you started?
- a. PROBES:
  - i. Heavy use (one or more times per day)
  - ii. Moderate use (few times per week)
  - iii. Light use (once a week or a few times a month)
  - iv. Very light (less than once a month)

16. What type of substances do you currently use?
- a. PROBES
  - i. If crystal meth- do you inject or smoke or both?
  - ii. If opioids- inject or smoke, or pill?
- b. **If YES to injection ask question 18, if NO skip question 18**

17. How would you describe your current substance use?
- a. PROBES
  - i. Several times per day (one or more)
  - ii. Few times a week
  - iii. Once a week or a few times a month
  - i. Less than once a month

18. How do you access the substances you use?
- a. PROBE
  - i. Do you get it yourself or does someone else get them for you?)

This next series of questions we are interested in better understanding the practices you use to inject drugs.

For peer review only - http://bmjopen.bmj.com/site/about/guidelines.xhtml

Covid-19 Pandemic Concealing a Syndemic of Concern: Sex, Gender, Methamphetamine and Sexually Transmitted and Blood Borne Infections in People Living with HIV in Manitoba

19. If you use substances that you inject, how often do you:

a. **Use a new needle, spoon, sterile water or filter?**
   i. All of the time, some of the time, rarely or never?
   ii. If only some of the time or never- what are the barriers to accessing safe injection supplies?

b. **Inject with a needle or syringe used by somebody else?**
   i. All of the time, some of the time, rarely, or never?

c. **Clean and disinfect the injection site?**
   i. All of the time, some of the time, rarely, or never?

d. **Prepare using a spoon, water, or filter used by somebody else?**
   i. All of the time, some of the time, rarely, or never?

e. **Have a safe place to use?**
   i. All of the time, some of the time, rarely, or never?
   ii. If yes, what makes this place safe?
   iii. If no, what makes this place unsafe?

20. Do you know where to find harm reductions supplies?

a. PROBE
   i. If yes, where do you get them from?
      1. What supplies do you get?
      2. How often do you get harm reduction supplies?

21. Have you experienced any violence related to your substance use?

a. PROBE
   i. If yes, and you feel comfortable, can you please tell me what happened?

22. What would make using substances safer for you?

a. PROBE
   i. Better access to harm reduction supplies
   ii. Safe injection site
   iii. Decriminalization of drug use
   iv. Safe person to use drugs with

23. Does using substances affect your ability to access HIV care & services?

a. PROBES
   i. If yes, in what ways?

For peer review only - http://bmjopen.bmj.com/site/about/guidelines.xhtml

Covid-19 Pandemic Concealing a Syndemic of Concern: Sex, Gender, Methamphetamine and Sexually Transmitted and Blood Borne Infections in People Living with HIV in Manitoba

24. How did the COVID-19 pandemic impact your substance use?

a. PROBE
    i. What changes in your pattern of use- availability, safety of substance, harm reduction supplies?
    ii. Changes to type of substance used or ways of using- did you use more of one substance or start injecting instead of smoking?

25. What do you think is the best way [how do you want] to learn about prevention and treatment of STBBI's and harm reduction practices?

26. Is there anything else you want to tell us today?

That is the end of the interview portion of our time together today.

Thank you very much for speaking with me. I really appreciate you taking the time today to do this. I know some of these questions were very personal and might have brought up some emotions for you. If you need support after our chat, I can provide you a list of resources ***[give participant mental health support resource document].*** *[Insert name of counsellor or Elder] is available to speak with you now if you would like that support. You can also speak to [Participating Organization] and they can direct you to any other supports you can access.*

Now that we have completed the interview portion of the interview, we will take a short break ***[discuss with the participant what they need to do- offer Elder support, food, break to walk around, smudge ceremony, stretch/move their body, etc.].*** *When we come back together, I will ask you to please fill out three short surveys. This should take another 30-45 minutes to complete. After that if there are things, you want to add to this discussion, we will make sure you have enough time to do that.*

For peer review only - http://bmjopen.bmj.com/site/about/guidelines.xhtml

Participant Survey: Demographics & Life Circumstances

The following short questionnaire asks questions about you, such as your age, identity, where you live, your experiences with substance use, your sexual practices, and other life circumstances you may have encountered. This information is very helpful as it provides a better understanding of your experiences as a person living with HIV. We understand that these questions are very personal and intimate, and we appreciate your responses. **There is no judgement about anything you share with us**. Every person has different life experiences and situations that affect their health and well-being. Some of these questions may not apply to you, **please only answer what is relevant to your life**.

Please **circle the answer(s) that applies to you, or write your answer in the space provided. If there are questions you do not feel comfortable answering, please select "Prefer not to say".** Please ask your interviewer if you would like them to better explain any of the questions.

Thank you for taking part in our survey!

PART A: Demographics

1. What is your date of birth? Day: ______/Month: ______/Year: _______

2. How old are you?

____

3. What gender identity best describes you?

a. Woman
b. Man
c. Transgender woman
d. Transgender man
e. Non-binary (agender, genderfluid, pangender, genderqueer, gender nonconforming, etc.)
f. Two-Spirit
g. Other
Which? ______________________
h. Prefer not to say

For peer review only - http://bmjopen.bmj.com/site/about/guidelines.xhtml

4. What sex were you assigned at birth? (as shown on your original birth certificate)
   a. Female
   b. Male
   c. Intersex
   d. Prefer not to say

5. What sexual orientation best describes you?
(Please select all that apply to you)

   a. Lesbian
   b. Gay
   c. Bisexual
   d. Asexual
   e. Heterosexual/Straight
   f. Pansexual
   g. Other
      Which?____________________
Prefer not to say

6. What cultural background best describes you?
(Please select all that apply to you)
   a. Indigenous
      i. First Nations
      ii. Métis
      iii. Inuk
      iv. Other ____________________
      v. Unspecified ____________________
   b. African
   c. White/European
   d. East/Southeast Asian
   e. South Asian
   f. South East Asian
   g. Hispanic or Latinx
   h. Middle Eastern
   i. Other (please specify) ______________________
   j. Prefer not to say

For peer review only - http://bmjopen.bmj.com/site/about/guidelines.xhtml

7. What is your marital status?

a. Single
b. Married
c. Divorced
d. Common-law
e. Widowed
f. Other
Which________________________________
g. Prefer not to say

8. What languages do you speak? **(Please select all that apply to you)**

Languages	Yes	No	Which (please specify) *
English			
French			
Cree			
Dakota			
Dene			
Inuktitut			
Michif			
Ojibway			
Oji-Cree			
Filipino			
Spanish			
German			
Ukrainian			
Punjabi			
Other (please specify)			
Prefer not to say			

*It applies just for “Other” option answers

For peer review only - http://bmjopen.bmj.com/site/about/guidelines.xhtml

9. What <u>religion</u> best describes you? **(Please select all that apply to you)**

a. Traditional Indigenous
b. Christianity
    i. Catholicism
    ii. Witness of Jehovah
    iii. Evangelical
    iv. Protestant
    v. Orthodox
c. Buddhism
d. Hindu
e. Jewish
f. Muslim
g. Sikh
h. Other Religion_________________________
i. No religious affiliation
j. Prefer not to say

10. What is <u>the highest level of education</u> you have <u>completed</u>?

a. K-12
    Which was the last grade? ______________
b. Certificate, diploma, vocational course from an educational institution
c. Bachelor's degree
d. Master's degree
e. Doctorate
f. Other, which _________________________
g. Prefer not to say

11. Where do you reside? (City/Town/Rural Community/Reserve)

a. _____________________________________

For peer review only

For peer review only - http://bmjopen.bmj.com/site/about/guidelines.xhtml

12. The following question are about **difficulties you may have doing certain activities**. Please tell us only about problems that **have lasted or are expected to last for six months or more**. Mark your response with an **X** for each difficulty.

Difficulties	No	Sometimes	Often	Always	Prefer not to say
Seeing					
Hearing					
Walking, using stairs, using hands or finger or doing other physical activities					
Learning, remembering, or concentrating					
Any emotional, psychological, or mental health conditions? (Anxiety, bipolar disorder, substance use, anorexia, etc)					

For peer review only

PART B. Income and Housing

Now we will ask you some questions related to your income and housing. These answers will allow us to better understand the resources that people living with HIV may need to support their health and well-being.

13. Are you currently employed?

a. Yes
b. No
c. Prefer not to say

14. What is your main source or source(s) of income?

____

15. Which of the following is your approximate yearly income?

a. less than 10,000 CAD/year
b. 10,000 – 19,999CAD/year
c. 20,000 - 29,999 CAD/year
d. 30,000 - 39,999 CAD/year
e. 40,000 - 49,999 CAD/year
f. More than 50,000/year
g. Prefer not to say

For peer review only - http://bmjopen.bmj.com/site/about/guidelines.xhtml

16. **Please** mark with an **X** any items that you currently **have** **or** **do not** have enough income to cover. **(Please select all that apply to you)**

Items	Have Enough	Do not have Enough	Prefer not to say	Does not apply
Housing (rent, hydro, water)				
Transportation				
Food				
Clothing				
Childcare				
Pet Care				
Healthcare				
Other (please specify)_______________				

17. What is your current living situation? **(Please select all that apply to you)**

Living situation	Yes	No	Which (please specify) *
Living alone (house/apartment)			
Living with partner (married, common-law or relationship)			
Living with children			
Living with roommates			
Living with extended family members			
Experiencing housing instability (insecure housing, shelter, transitional housing, houseless)			
Other (please specify)			
Prefer not to say			

*It applies just for "Other" option answers

Questions 17, 18, and 19 only apply to people who are experiencing housing instability.

18. How long have you experienced housing instability?

________ Days ________ Months ________ Years

For peer review only - http://bmjopen.bmj.com/site/about/guidelines.xhtml

19. **If you are experiencing housing instability,** which of the following are you experiencing? **(Please select all that apply to you)**

Living situation	Yes	No	Which (please specify) *
Insecure housing (month to month, threats by landlord to leave, unsure how you will pay rent next month)			
Couch surfing			
Sleeping rough (sleeping in the streets)			
Staying at a shelter			
Short-term housing program			
24/7 space			
Other (please specify)			
Prefer not to say			

*It applies just for "Other" option answers

20. **If you are experiencing housing instability,** did you experience housing instability before or after being diagnosed with HIV?
    a. Before I was diagnosed
    b. After I was diagnosed
    c. Before and after I was diagnosed
    d. Other
       Which?______________________
    e. Prefer not to say

PART C: Criminal Justice System

The next few questions we want to ask you are related to your experiences with the criminal justice system. We are asking these questions as recent HIV infections have shown a number of infections in people who were incarcerated. These questions will help us to also understand how policing and surveillance may be affecting your life and health. We do not judge any of your answers and we are not affiliated in any way with police or the justice system.

For peer review only - http://bmjopen.bmj.com/site/about/guidelines.xhtml

21. Have you ever been incarcerated? (prison, jail, correctional facility)

	Initial date (from)	Final date (to)
Yes	Month: ________/Year: ________	Month: ________/Year:________
	Month: ________/Year: ________	Month: ________/Year:________
	Month: ________/Year: ________	Month: ________/Year:________
No	(Skip to Part D)	
Prefer not to say		

22. Did you receive an HIV positive diagnosis while incarcerated?

a. Yes
b. No **(Skip to question 23)**
c. Prefer not to say

23. If you did receive a positive diagnosis while incarcerated, who told you about your diagnosis? **(Please select all that apply to you)**

a. Physician
b. Nurse
c. Administration staff
d. Prison Guard
e. Other___________________
f. Prefer not to say

24. Did you experience discrimination while incarcerated because of your HIV diagnosis?

a. Yes
b. No **(Skip to question 25)**
c. Prefer not to say

For peer review only - http://bmjopen.bmj.com/site/about/guidelines.xhtml

25. If you did experience discrimination because of your HIV diagnosis, who did you experience discrimination from? **(Please select all that apply to you)**

	Yes	No	Who (please specify) *
Other incarcerated people			
Prison guards			
Healthcare staff			
Administration staff			
Child and Family Services (CFS)			
Other (please specify)			
Prefer not to say			

*It applies just for "Other" option answers

26. Have your experiences with the criminal justice system affected your ability to access HIV care and other health care services after you were released?

a. Yes
b. No
c. Prefer not to say

27. Please select with an **X** the services you had access to while incarcerated

Services	Had Access	Did not have access
Primary health care services (regular physicals, doctor appointments)		
HIV related care (medication, regular blood testing, specialist appointments)		
STBBI testing		
Harm Reduction Supplies (condoms, syringes, pipes)		
Child and Family Services (CFS)		
Mental health services and counselling		
Ceremony		

*Sexually Transmitted and Blood Borne Infections

For peer review only - http://bmjopen.bmj.com/site/about/guidelines.xhtml

28. Are you currently under correctional supervision (e.g., bail conditions, probation, or parole)?

	Start date (from)	Final date (to)
Yes	Month: ________/Year: ________	Month: ________/Year:________
No	**(Skip to Part D)**	
Prefer not to say		

PART D: Sexual Practices

Now we would like to ask you some questions about your sexual practices. Please answer what you are comfortable with. We are asking these questions as people can acquire HIV through sexual activity but that is not the only way. The reason we are asking these questions is to help us better understand the sexual practices of people living with HIV which can be useful when informing safe sex education.

29. Are you currently sexually active?

a. Yes
b. No
c. Prefer not to say

30. In the 12 months have you had sex with **(Please select all that apply to you)**

	Yes	No	Which (please specify) *
Women			
Men and women			
Men			
Non-binary person			
Two-spirit person			
Other (please specify)			
Prefer not to say			

*It applies just for "Other" option answers

31. In the past 12 months how many sexual partners have you had?

a. ________________
b. Prefer not to say

For peer review only - http://bmjopen.bmj.com/site/about/guidelines.xhtml

32. When engaging in sexual activity, do you use any of the following safe sex protections?

Protections	All of the time	Some of the time	Never
Condoms			
Dental dams			
Pre-exposure prophylaxis (Prep)			
Post exposure prophylaxis (Pep)			
Regular STBBI testing			
Other (please specify)________________			

33. How easy was it to get safe-sex protections before and during the COVID-19 pandemic?

a. Easier before COVID-19 pandemic
b. No difference before and during COVID-19 pandemic
c. Easier during COVID-19 pandemic
d. Prefer not to say

34. Do you use a form of contraception? (Birth control, condoms)

a. Yes
b. No **(Skip to questions 36)**
c. Prefer not to say

35. Mark with an **X** your use of the following forms of contraception:

Contraception	Yes	No	Which (please specify)
Short-acting hormonal methods (oral birth control)			
Long-acting reversable contraceptive (IUD)			
Barrier Methods (condom, sponge, cervical cap)			
Natural rhythm methods			
Sterilization (tubal ligation or vasectomy)			
Other (please specify)			
Prefer not to say			

For peer review only - http://bmjopen.bmj.com/site/about/guidelines.xhtml

36. How often do you get blood tests for sexually transmitted infections and blood born infections (Hep C, Syphilis, Gonorrhea)?

a. Before engaging in sexual activity with a new partner
b. Once a month
c. Every few months
d. Once a year
e. When my doctor/nurse practitioner recommends
f. Never
g. Other
When (please specify)?________________________________
h. Prefer not to say

PART E: Substance Use

We would now like to ask you about your substance use. These questions may or may not apply to you, and we want to assure you that there is no judgment based on your use of substances. We are asking these questions to understand how to better support people who are living with HIV who also may use substances

37. Do you currently use substances? (alcohol, marijuana, crystal meth, crack, cocaine)

a. Yes
b. No
c. Prefer not to say

38. Please fill in this chart. For every substance please select with an (x) the quantity and length of consumption

**Substance**	**Consumption**			**Quantity Consumed**					**Time of consumption**			
	**Never**	**Past**	**Present**	**Quantity**	**Daily**	**Weekly**	**Monthly**	**Yearly**	**Length**	**Days**	**Months**	**Years**
Tobacco				# of cigarettes								
Alcohol				# of drinks								
Inhaled Substances				# of inhalations								
Smoked Substances				# of times smoking								

For peer review only - http://bmjopen.bmj.com/site/about/guidelines.xhtml

Injected Substances				# of injections								

39. How old were you when you started using substances?

____
Age

40. What substance did you start with?

____
Name(s) of substance

41. Please describe your current and past substance use by using an **X** in every line

	Heavy use (one or more times per day)	Moderate use (few times per week)	Light use	Very light use (once a week or a few times a month)	Not using
Current substance use					
Substance use when you started					

42. Does substance use impact your sexual practices in any of the following ways, select with an **X** if yes or no:

**Impacts**	**Yes**	**No**	**Prefer not to say**
I am **more likely** to engage in sex with multiple partners when using substances			
I am **less likely** to engage in sex with multiple partners when using substances			
I am **less likely** to use protection when engaging in sexual activity when using substances			
I am **more likely** to use protection when engaging in sexual activity when using substances			
Other, which			

For peer review only - http://bmjopen.bmj.com/site/about/guidelines.xhtml

43. If you use substances that you inject, **how often do you**:

Practices	All of the time	Some of the time	Not very often	Never
Use a new needle				
inject with a needle or syringe used by somebody else				
Bleach or clean needles used by somebody else				
Prepare using a spoon, water, or filter used by somebody else				
Have someone else inject your substances				
Have a safe place to inject				
Know where to find harm reduction supplies				

PART F: Experiences with Violence

For the last portion of this survey, we want to better understand your experiences with violence. This section may or may not apply to you. The reason we are asking these questions is because during the COVID-19 pandemic there was a reported increase in experiences of violence, in particular intimate partner violence and domestic violence. There is also a higher rate of women who have been diagnosed with HIV in Manitoba over the past few years – and women, transgender, non-binary people, as well as those who identify as Indigenous and 2SLGBTQQIA+ are more likely to experience all forms of violence.

For peer review only - http://bmjopen.bmj.com/site/about/guidelines.xhtml

44. Throughout your life, have you ever experienced any of the following types of violence? **(Please select all that apply to you)**

Types of Violence	Any point in your life	Past 3 years	Currently
Physical violence			
Sexual violence			
Stalking, harassment			
Emotional abuse			
Intimate Partner Violence (violence perpetrated by a partner in a romantic or dating relationship)			
Domestic Violence (violence among people in a domestic situation- can include any type of family, or roommate)			
Cyberstalking/cyberbullying			
Child abuse			
Psychological / mental violence			
Other, which			
Prefer not to say			

45. **If you are currently experiencing Intimate Partner Violence**, mark with an **X** the ways it impacts you:

**Impacts**	**Yes**	**No**	**Prefer not to say**
I am not able to attend HIV clinic appointments or HIV related care because of my partner (Partner controls my time and schedule, money for health care costs, etc.)			
I am afraid of my partner finding out about when I am going to doctor appointments or taking medication			
My health care is not impacted, and I am able to attend all my appointments			
Other, which			

46. Have you received any support or health care services to support you with healing from experiences of violence (trauma counselling, psychological support, traditional healing)?

a. Yes
b. No **(Skip to question 48)**
c. Prefer not to say

For peer review only - http://bmjopen.bmj.com/site/about/guidelines.xhtml

47. If you have received support following your experiences with violence, which of the following have you received? **(Please select all that apply to you)**

Supports	Yes	No	Which (please specify) *
Counselling (Trauma counselling, Domestic Violence counselling)			
Psychological Support			
Support groups			
Traditional healing			
Domestic Violence shelter			
Women's centre			
Other (please specify)			
Prefer not to say			

*It applies just for "Other" option answers

48. Has anyone shared your HIV status without your consent?

a. Yes
b. No
c. Prefer not to say

That brings us to the end of the survey. Thank you again for taking the time to fill out this survey. We really appreciate your time and willingness to share your experiences with us. If you have any questions or would like to talk more with the interviewer, or support person, that can happen now. We have also created a resource sheet to access support or counselling if you would like a copy.

For peer review only - http://bmjopen.bmj.com/site/about/guidelines.xhtml

Service Provider Interview Question Guide

To be completed by facilitator	***Facilitator Name***		
Participant ID:			
Participating Organization:			
Location:			
Interview date:			
Interview time:	Start	End	Duration
Confirmation of consent signed:			

Thank you for agreeing to speak with me today. My name is [INSERT] and my pronouns are INSERT]. My role with this project is as a [Research Assistant, Research Associate].

Before we start the formal interview questions, I would like to remind you that we are here to speak about your experiences as a service provider [clinic program providers and/or social service employee] to provide additional perspectives on institutions, structures and policies related to HIV Care in Manitoba. The interview should take approximately 45 to 60 minutes.

Everything you share with me will be kept confidential. I also want to remind you that that our conversation will be recorded so your words are captured as accurately as possible, but the recording will be kept securely and destroyed once the study is over.

You have been asked to participate in this interview as you:

1) *Are a clinic service provider or social service employee who works with and/or provides services to people living with HIV or other STBBI's in Manitoba [and/or people who inject drugs]*

Does this statement apply to you?
Yes
No

For peer review only - http://bmjopen.bmj.com/site/about/guidelines.xhtml

For peer review only

[If no, relay that they are not eligible to participate in the study. Thank them for their time, and end interview].

For the purpose of our discussion, STBBI Services may include:

- *HIV testing / care*
- *Hepatitis C testing / care*
- *Syphilis testing / care*
- *Other sexually transmitted infection (STI) testing /care*
- *Obtaining safe sex products (condoms, dental dams, pre/post exposure prophylaxis*
- *Information about STBBI testing/care*
- *Counselling related to STBBIs*
- *Community services or supports related to STBBIs*

Do you have any questions for me before we begin?

1. **Could you please describe your role?**
    a. PROBES
        i. Type of organization (Clinic, CHC, Community Organization)
        ii. Position Title
        iii. Main job duties
        iv. Schedule

2. **What services does your organization provide?**
    a. PROBES
        i. Women's health, mental health, addictions?

3. **Please tell me about what services are working well for your clients/patients living with HIV?**
    a. PROBES
        i. Specific supports or programs
        ii. Accessibility
        iii. Campaigns
        iv. New services to deal with COVID-19

4. **What barriers to accessing or remaining in care have you identified for people living with HIV?**
    a. PROBES
        i. Attitudes to some clients
        ii. Limited hours or sites
        iii. Shortages of supplies or staff
        iv. Person's gender identity
        v. Stigma towards infectious diseases, substance use, race, etc.

For peer review only - http://bmjopen.bmj.com/site/about/guidelines.xhtml

5. **What barriers have you identified for PLHIV who are actively using substances that may prevent them from accessing care?**
    a. PROBES
        i. Attitudes of staff
        ii. Administrative regulations
        iii. Stigma associated with substance use, race, gender
        iv. Behaviour challenges related to substance use

6. **What major changes have you noticed in your clients/patients due to the COVID-19 pandemic?**
    a. PROBES
        i. Increased drug use
        ii. Increased experiences of violence
        iii. Mental health challenges
        iv. Housing instability
        v. Income instability
        vi. Incarceration
        vii. Increase in sex work
        viii. Suicidal ideation
        ix. Anything else?

7. **What are some examples of success factors that you have seen clients employ during the COVID-19 pandemic to ensure they continue to get the care they need?**

8. **How has the COVID-19 pandemic affected the services in your organization?**
    a. PROBES
        i. Changes of hours
        ii. Not enough staff to meet client needs
        iii. Virtual platform challenges
        iv. Increased needs of clients related to drug use
        v. Strain on health care system & referral services
        vi. Impact on staff to deliver services
            1. Burnout
            2. Getting sick themselves
            3. Not enough training for new staff

9. **How has the COVID-19 pandemic affected you personally as a service provider?**
    a. PROBES
        I. Mental health challenges
        II. Burnout
        III. Secondary trauma
        IV. Increased work load
        V. Covid-19 reinfections

For peer review only - http://bmjopen.bmj.com/site/about/guidelines.xhtml

10. How do you support people whose needs fall outside of the services you offer?
   a. PROBE
      i. Collaborate with other health providers or community agencies to connect people to these services
      ii. Relationships with other providers in the community

11. What strategies do you currently use to support people living with HIV who use substances?
   a. PROBES
      i. What additional training, resources, education do you need?

12. What is your understanding of harm reduction and how do you apply this in your work?
   a. PROBES
      i. Harm reduction- knowledge, attitudes
      ii. What else do you want to learn about harm reduction?

13. What strategies do you use to support your clients with prevention of STBBI's?
   a. PROBES:
      i. PrEP, STBBI testing, counselling/mental health support
      ii. Education/sharing knowledge about STBBI's- how they are transmitted, how are the treated?

14. What do you personally do or what does your organization do to create a safe space/environment for clients?
   a. PROBES
      i. Environment- accessibility, meeting clients where they are at, safety
      ii. Training- anti-racism, anti-oppression
      iii. Policies- sexual harassment, violence, covid safety, harm reduction
      iv. Practices- cultural safety, trauma informed care

15. Can you tell me what you think about the policies in Manitoba related to HIV?
   a. PROBES
      i. Specific policies working against ability to get, receive or remain engaged in care
      ii. Policies currently working well to support PLHIV
      iii. Policies you would create to support you in your work as a service provider

16. What are your suggestions for policy change or program enhancements- that would both support PLHIV and service providers delivering care?
   a. PROBES
      i. Additional options for care, additional services
      ii. Referrals to other services

For peer review only - http://bmjopen.bmj.com/site/about/guidelines.xhtml

iii. Bundling services in one appointment or location (wrap around care)

17. What additional resources are needed to better support people living with HIV in Manitoba?

a. PROBES

i. Prevention- i.e. PREP
ii. Additional options for care
iii. Knowledge about STBBI's- how they are transmitted, how are the treated?

18. What do you think is the best way [how do you want] to learn about prevention and treatment of STBBI's and harm reduction practices?

19. We are asking everyone involved in this study to share their gender. If you are comfortable, can you please tell us how you identify?

That is the end of our interview today. *Before we go, I wanted to know if you wanted to share anything else with me? Or if you think we should have asked you about something else?*

Thank you very much for speaking with me. *We really appreciate your time and efforts and the valuable information you have shared with us today.*

For peer review only - http://bmjopen.bmj.com/site/about/guidelines.xhtml
